# Thank You to Our Peer Reviewers in 2023

**DOI:** 10.1111/hex.70075

**Published:** 2024-11-05

Carolyn Chew‐Graham (Editor‐in‐Chief), Parisa Aslani, Sarah Derrett, Michelle Flood, Clarissa Giebel, Lisa D. Hawke, Andrea Hilton, Kerry Kuluski, and Georgina Warner (Associate Editors)

We would like to thank all the reviewers from around the world who have provided expert advice to ensure publication of the best quality articles in *Health Expectations*. Their feedback is crucial to improve the quality of manuscripts and to clarify concepts, develop theories, and critically analyse and evaluate specific policies and practices. The names of those who reviewed the manuscripts are listed below.

Our warmest gratitude goes to all of them, and especially to our top 10 reviewers.

Marianne Saragosa

Klejda Harasani

Mark Harris

Ashfaq Chauhan

Robin Gauld

Brooke Allemang

Muhammad Muneeb Hassan

Natasha Sheikhan

Susan Rifkin

Nagihan Sim Aygul

**Table:** 

Lena	Aadal
Katri	Aaltonen
Whitley	Aamodt
Bizhan	Aarabi
Ghazal	Aarabi
Bernt Boegvald	Aarli
Emily L.	Aaronson
Neil	Aaronson
Nicole L.	Aaronson
Asrenee	Ab Razak
Maria	Abajo del Rincón
Zuhal	Abasıyanık
Julie	Abayomi
Samira	Abbasgholizadeh Rahimi
Daniel E.	Abbott
Penny	Abbott
Stephen	Abbott
Cynthia	Abbott‐Gaffney
Christina	Abdel Shaheed
Mostafa	Abdelsalam
Abbas	Abdollahi
Azidah	Abdul Kadir
Abdul‐Fatawu	Abdulai
Khatijah Lim	Abdullah
Abdul‐Kareem	Abdul‐Rahman
Ephrem	Abebe
Md. Anwarul	Abedin
Sedigheh	Abedini
Gary	Abel
Julia	Abelson
Maria	Aberg
Alice	Abernathy
Purva	Abhyankar
Gilbert Abotisem	Abiiro
Seye	Abimbola
Clare	Abley
Tineke	Abma
Shahla	Abolhassani
Hanan	Aboumatar
Olufunmilola	Abraham
Charlotte	Abrahamsen
Charlotte	Abrahamsen
Roberto L.	Abreu
Martha	Abshire Saylor
Martina	Absinta
Ahmad Ihsan	Abu Bakar
Eman	Abukmail
Hammoda	Abu‐Odah
Pinar	Acar‐Ozen
Ceren	Acartürk
Taieb	Ach
Lalatendu	Acharya
Subhendu Kumar	Acharya
Helen	Achat
Roel C. A.	Achterbergh
Benjamin W.	Ackermann
Darlene	Acorda
Nicola	Acquarone
Chiara	Acquati
Geraldine	Adam
Philippe C. G.	Adam
Shelin	Adam
Thomas V.	Adamkiewicz
Corey	Adams
Mary	Adams
Nicola	Adams
Samantha	Adams
Sherri	Adams
Yenupini Joyce	Adams
Brynn	Adamson
Elizabeth	Adamson
Metadel	Adane
Karthik	Adapa
Nicola	Adderley
Michael	Addis
Samia	Addis
Emmanuel	Adebayo
Demilade A.	Adedinsewo
Adesola	Adenike Ogunfowokan
Desmennu	Adeyimika
Kamala	Adhikari
Pratik	Adhikary
Obi Peter	Adigwe
Achini	Adikari
Mint H. R.	Aditama
Nicholas Kofi	Adjei
Samuel	Adjorlolo
Douglas	Adler
Novia	Admodisastro
Prajakta	Adsul
Joseph	Adu
Georges	Adunlin
Patient and Public	Advisors
Leila	Aflatoony
Chris B.	Agala
Eirini	Agapidaki
Meera	Agar
Agapi	Agelaki
Janne	Agerholm
Hanne	Agerskov
Ajay	Aggarwal
Monica	Aggarwal
Vishal	Aggarwal
Tamás	Ágh
Branka	Agic
Gudmund	Ågotnes
Heloise Fernandes	Agreli
Martin	Agrest
Stephanie	Agtarap
Gloria	Aguayo
Andrés	Agudelo‐Suárez
Urko	Aguirre
Bruno	Agustini
Eldad	Agyei‐Manu
Charles	Agyemang
Sheikh Iqbal	Ahamed
Amy	Ahern
Malene	Ahern
Tracey	Ahern
Viktor H.	Ahlqvist
Gerd	Ahlström
Jasjit	Ahluwalia
Faraz S.	Ahmad
Kabir	Ahmad
Kashif	Ahmad
Annika	Åhman
Rukhsana	Ahmed
Tashrik	Ahmed
Abubaker	Ahmed
Nehad J.	Ahmed
Nisar	Ahmed
Sadia	Ahmed
Salina	Ahmed
Shenaz	Ahmed
Soojung	Ahn
Alka	Ahuja
Jun	Aida
Rachel	Ainley
Kelly	Ainsworth
Bruce	Aisthorpe
Stephen	Aita
Peter	Aitken
Masao	Aizawa
Howard J.	Aizenstein
Budi	Aji
R.	Ajjan
Puneeta	Ajmera
Shilpi	Ajwani
Neziha	Akalin
Joseph	Akanuwe
Terrah Foster	Akard
Fuad Husain	Akbar
Ayşegül	Akca
Joseph	Aked
Kristina Bakke	Åkerblom
Mia	Akiba
	Akkuş
Elie	Akl
Olufemi	Akodu
Ersin	Akpinar
Suleman	Aktaa
Rıdvan	Aktan
Puren	Aktas
Nuray	Aktay Ayaz
Shahinoor	Akter
Shahriar	Akter
Gokcen	Akyurek
Dania E.	Al Agili
Jameela	Al Salman
Faiza	Al Zadjali
Olamide	Alabi
Maissa	Al‐Adhami
Kaat	Alaerts
Samah	Alageel
Esra	Alagoz
A. H.	Alamoodi
Liane Peña	Alampay
Turki	Alanzi
Sinaa	Alaqeel
Akke	Albada
Alessio	Albanese
Loai	Albarqouni
Loai	Albarqouni
Angela	Alberga
Gwenda	Albers
Jay L.	Alberts
Charlotte	Albury
Abigail	Albutt
Abigail	Albutt
Francesca	Alby
Mirko	Alde'
Hayley	Alderson
Sarah	Alderson
Colleen	Aldous
Vania	Aldrete‐Cortez
Lynley	Aldridge
Anna Christina	Alegiani
Margarita	Alegria
Janeth	Aleman‐Tovar
Razieh	Alemi
Kassahun	Alemu
Karyn	Alexander
Obrey	Alexis
Sara	Alfieri
Fahad	Algarni
Noora	Alhajri
Tarek	Alhamad
Mohammed	Al‐Hanawi
Isahaque	Ali
Askal Ayalew	Ali
Habiba	Ali
Khalid	Ali
Lilas	Ali
Raghib	Ali
Soegianto	Ali
Alaa	Ali Abd‐Alrazaq
Mohammad	Ali Cheraghi
Nasrollah	Alimohammadi
Mohammad	Alizadeh
Hiyam	Al‐Jabr
Hisham	Aljadhey
Maryam	Alkandari
Rama	Alkhalaileh
Suad	Alkhamis
Abdullah	Alkhawaldeh
Sameer A.	Alkubati
Shereen	Allaham
Hamid	Allahverdipour
Saleema	Allana
Anne‐Francoise	Allaz
Alina M.	Allen
Brian	Allen
Caitlin G.	Allen
Chris	Allen
Dawn	Allen
Jacqueline	Allen
Jacqui	Allen
James	Allen
Lisa	Allen
Natalie E.	Allen
Pauline	Allen
Sabine	Allida
Olivia	Alliott
Mary Kathryn	Allison
Rhoda	Allison
Kelly	Allott
Gail	Allsopp
Gail	Allsopp
Megan	Allyse
Vibeke Marie	Almaas
Ghaith	Almahadin
Jaime P.	Almandoz
Aysha	Almas
Nihad A.	Almasri
Nisha	Almeida
Hani	Almoallim
Yasir	Almuzaini
Roy	Aloni
Aquilino	Alonso
Ricardo	Alonso
Pablo	Alonso‐Coello
Amani	Al‐Oraibi
Saleh	Aloraini
Brian	Alper
Ash B.	Alpert
Jordan	Alpert
Emily	Alsentzer
Bushra	Alshammari
Mohammed	Al‐Talib
Hala	Altawil
Rieke	Alten
Mustafa	Altıntaş
Daniel M.	Altmann
Drew	Altschul
Lisa	Altshuler
Jane	Alty
Ruben	Alvarado
Geovanny	Alvarado‐Villa
Willyane de Andrade	Alvarenga
Gail	Alvares
Elizabeth	Alvarez
Elisabete	Alves
Helena Maria Baptista	Alves
Ala	Alwan
Nisreen	Alwan
Ahmed M.	Al‐Wathinani
Haitham	Alzghaibi
Mungunzul	Amarbayan
Domenico D.	Amario
Sugalya	Amatachaya
Imanol	Amayra
Anshula	Ambasta
Leire	Ambrosio
Shazhan	Amed
Alice	Ames
Stefanie G.	Ames
Teresa	Amezcua Aguilar
Alejandro	Amill‐Rosario
Cervantes‐Arriaga	Amin
Maryam	Amini
Ziv	Amir
Sohrab	Amiri
Joshua	Amo‐Adjei
Mary	Amoakoh‐Coleman
Oluwakemi	Amodu
Fábio Ferreira	Amorim
Jason	Amorim
Nicola	Amoroso
Holly	Amos
Michel	Amouyal
Agbessi	Amouzou
Delena	Amsters
Hubert	Amu
Yuanyuan	An
Dana	Anaby
Evdokia	Anagnostou
Anna	Anåker
Vikram	Anand
Kanthee	Anantapong
Kanthee	Anantapong
Kanthee	Anantapong
Joseph	Anaya
Nada	Andelic
Claire	Anderson
David M.	Anderson
Elizabeth S.	Anderson
Helen	Anderson
Jill	Anderson
Kayla N.	Anderson
Melissa N.	Anderson
Natalie Elizabeth	Anderson
Nathaniel	Anderson
Ruth A.	Anderson
Sharon	Anderson
Rebecca	Anderson‐Kittow
Maria L. E.	Andersson
Thomas	Andersson
Viktor	Andersson
Yvonne	Andersson
Shuntaro	Ando
Barbara	Andraka‐Christou
Paula	Andreasen
Frida	Andréasson
Panaite	Andreea‐Cătălina
Lobiuc	Andrei
Elisa	Andrenelli
Nadine	Andrew
Jac J. W.	Andrews
Jane	Andrews
Jean	Andrews
Karl	Andriessen
Maren	Andvik
Lisa	Anemaat
Blake	Angell
Nick	Angelopoulos
Heather	Angier
Ronald	Anguzu
Anthony	Angwin
Sabrina	Anjara
Gulnaz	Anjum
Ann	Anka
Samuel	Ankomah
Lisa	Anna Marie Edmonds
Bethany	Anne Bruno
Anne	Anne Moorhead
Michael	Annear
Md Tarique Jamal	Ansari
Manou	Anselma
Lena	Ansmann
Samantha	Anthony
Kirsti Sarheim	Anthun
Lukasz	Antkowiak
Ailin	Anto
Boivin	Antoine
Grazia	Antonacci
Michele	Antonelli
Chrystalina A.	Antoniades
Marichu	Antonio
Martina	Anto‐Ocrah
Barbara	Antunes
Leonardo Santos	Antunes
Fahim	Anwar
Hıdır	Apak
Elizabeth M.	Aparicio
Kaija	Appelqvist‐Schmidlechner
Ramana	Appireddy
Karine Toupin	April
Deborah	Apthorp
Yves Saint James	Aquino
Mohammad	Arab
Sawako	Arai
Szilvia	Arany
Lia	Araújo
Alicia	Arbaje
Karen	Arblaster
Patrick	Archambault
Sara	Ardila‐Gomez
Ríona Mc	Ardle
Suparb	Aree‐Ue
Margarida	Areia
Hannah	Arem
Roger	Arend
Kristina	Areskoug‐Josefsson
Kristofer	Årestedt
Stavropoulou	Areti
Lilith	Arevshatian
Rob	Argent
Lewis	Ariane
Merita	Arini
Manuel	Armayones
Jo	Armes
Christopher	Armitage
Hannah	Armitt
Megan	Armstrong
Melissa	Armstrong
Natalie	Armstrong
Natalie	Armstrong
Alessandro	Armuzzi
Sidse	Arnfred
Sabine	Arnolds
Cathrine	Arntzen
Kjell Arne	Arntzen
Herbert D.	Aronow
Amit	Arora
Himanshu	Arora
Tuba	Arpaci
Bruce	Arroll
Geneviève	Arsenault‐Lapierre
Gökmen	Arslan
Şeyma	Arslan
Walaszek	Art
David	Arterburn
Niamh	Arthurs
Juliette	Artignan
Anna	Artmann
Majid	Artus
Priti	Arun
	Arundell
Miho	Asano
Tom	Aschman
Zahid	Asghar
Jill	Ashburner
Darren	Ashcroft
Rachelle	Ashcroft
Stephen Andrew	Ashford
Kirsten	Ashley
Laura	Ashley
Emma	Ashworth
Mark	Ashworth
Gladys	Asiedu
Koula	Asimakopoulou
Sorayya	Askari
Deborah A.	Askew
Ayse Tana	Aslan
Fiona	Aspinal
Bronte	Aspin‐Wood
Pim	Assendelft
Tatiane	Assone
Sabrina	Assoumou
Lydia	Aston
Megan	Aston
Ayşe Merve	Ata
Samuel R.	Atcherson
Kathryn A.	Atchison
Kostas	Athanasakis
Helen	Atherton
Roger	Atinga
Annabelle Lin	Atkin
Karl	Atkin
Tiffany	Atkins
Omar F.	Attarabeen
Darush	Attar‐Zadeh
Francesco	Attena
Christiane	Attig
Clifford	Attkisson
Barbara	Atzori
Flora	Au
Isabelle	Aubin‐Auger
Kriss	Aubrey‐Bassler
Diane	Auderset
Carolyn M.	Audet
Vanhaudenhuyse	Audrey
Jeremy	Auerbach
Manon	Auffret
Astraea	Augsberger
Bourne	Auguste
Kurtis	Auguste
Federico	Augustovski
Hanna	Augustsson
Samantha	Ault
Myo Nyein	Aung
Justin	Aunger
Kirsten A.	Auret
Karijn	Aussems
Elizabeth	Austin
Nichole	Austin
Robin R.	Austin
Rosalynn	Austin
Peter C.	Austin
Áine	Aventin
Phoebe	Averill
Leah	Avery
Christina	Avgerinou
Richard	Aviv
Petra	Avramovic
Fazli Rabbi	Awan
Hassan	Awan
Yvonne	Awenat
Anna	Axelin
Alison M. El	Ayadi
Dr. Martin Amogre	Ayanore
Carole	Ayav
Sultan	Ayaz‐Alkaya
Selma	Aybek
Selma	Aybek
Yavuz	Ayhan
Paul	Aylin
Julie	Ayre
Matthew J.	Ayre
Julie	Ayres
Jaya	Aysola
Darshini	Ayton
Wan Mohd	Azam Wan Mohd Yunus
Ana	Azevedo
Nancy	Azevedo
laurent	azoulay
Peter	Azzopardi
Barrett	B.
Sarah	B. Mulkey
Laura	Baams
Leonard	Baatiema
Narges	Babakhani
Opeyemi	Babatunde
Alessandra	Babore
Loraine	Bacchus
Tamara	Backhouse
Catherine	Backman
Desiree	Backman
Maria	Backström
Maha	Badawi
Kerime	Bademli
Julia R.	Badger
Karen	Badger
Amira Saad	Badran
Fathima Azmiya	Badurdeen
Christoph	Baerwald
Dinesh Kumar	Bagga
Stéphanie	Baggio
Razieh	Bagherzadeh
Heather	Bagley
Won‐Myong	Bahk
Fatemeh	Bahramnezhad
Li	Bai
Lin	Bai
S. J.	Baig
Sol	Baik
Kasia	Bail
Jacqueline	Bailey
James	Bailey
Jannine	Bailey
Stacy Cooper	Bailey
Ross	Bailie
Jodie	Bailie
Antoine	Bailliard
Jessica	Baillie
Rebecca	Baines
Matthew	Bair
Kalyana Chakravarthy	Bairapareddy
Janis	Baird
Kathleen	Baird
Kathleen	Baird
Sabrina	Bajwah
Marieke A. R.	Bak
Tamilyn	Bakas
Amanda	Baker
Brittany	Baker
Elizabeth A.	Baker
Felicity A.	Baker
Helen	Baker
John	Baker
Caroline	Baker
Mary J.	Baker‐Ericzen
Nawar Diar	Bakerly
Christos	Bakirtzis
Trine Lise	Bakken
Ioannis	Bakolis
Neşe Burcu	Bal
Michèlle	Bal
Shari	Baldinger
Julie A.	Baldwin
Paola	Balestrieri
Geoff D. C.	Ball
Kylie	Ball
Mel	Ball
Soledad	Ballesteros
Claire	Ballinger
A.	Balogun‐Katung
Vanessa	Balounaïck‐Arowas
Jayson R.	Baman
Clare	Bambra
Claire	Bamford
Adeola	Bamgboje‐Ayodele
Olufikayo O.	Bamidele
Elizabeth	Bancroft
Rebecca	Band
Ananda S.	Bandyopadhyay
Amitava	Banerjee
Samprit	Banerjee
Somalee	Banerjee
Laura	Banfield
Michelle	Banfield
Marco	Bani
Jack	Banks
Davina	Banner
Yair	Bannett
K.	Bannister
Sheng	Bao
Paul	Barach
Stefan	Baral
Ayelet	Baram‐Tsabari
David	Baran
Mariela	Barani
Edwine	Barasa
Claire	Barber
Rosemary	Barber
Skye	Barbic
Verena	Barbieri
Edel Figueiredo	Barbosa‐Stancioli
Marco	Bardus
Beth	Bareham
Liron	Bar‐El
Serena	Barello
Serena	Barello
Alessio	Baricich
Kathryn	Barker
Justine	Barksby
April	Barnado
Jamie C.	Barner
Christopher	Barnes
Larisa	Barnes
Marian	Barnes
Rebecca	Barnes
Sarah	Barnes
Daisy	Barnetson
Katherine Gergen	Barnett
Magali	Barnoux
Jan	Barnsley
Lynn	Bar‐On
Damiano Giuseppe	Barone
Paul	Barr
Megan E.	Barra
Michael	Barras
Alexandra	Barratt
Manuel	Barreiro‐De Acosta
Peter	Barrett
Sean P.	Barrett
Iván	Barrios
Duncan	Barron
Sandra Garrido de	Barros
Emily	Barrow
Arden R.	Barry
Heather E.	Barry
Margaret	Barry
Nicole K.	Bart
Sandra	Barteit
Heike	Bartel
Sara	Bartels
Dennis	Barten
Douglas	Barthold
Rosalie	Bartholomew
Bernadette	Bartlam
Kate	Bartlem
Gillian	Bartlett
Larissa	Bartlett
Ruth	Bartlett
Lisa	Bartolmeo
Chris	Barton
Christian	Barton
Andrea	Barton‐Hulsey
Melanie	Barwick
Yael	Bar‐Zeev
Corey H.	Basch
M. D. Abu	Bashar
Osnat	Bashkin
Alexandra	Basilakos
M.	Baskaran
Deniz	Başkent
Tezcan Ozrazgat	Baslanti
Srijana	Basnet
Peivand	Bastani
Hilde	Bastiaens
Frances	Batchelor
Andrew	Bateman
Brenna	Bath
Ricardo	Batista
Kornelia	Batko
Alan M.	Batt
Katharine	Batt
Michele	Battié
Greta	Bauer
Lisa Ann	Baumann
Jennifer	Baumbusch
Annika	Baumeister
Karine	Baumstarck
Louise	Baur
John Robert	Bautista
Amen A.	Bawazir
Ruth	Baxter
Susan	Baxter
Marc	Bayen
Sabine	Bayen
Nathaniel D.	Bayer
Kerin	Bayliss
Gareth	Baynam
Latifa	Baynouna Al Ketbi
Mustafa	Bayraktar
Ece	Bayram
Jonathan	Bayuo
Sarah	Baz
Carmen	Beato
Angela	Beattie
Angela	Beattie
R. Mark	Beattie
T.	Beatty
Jennifer E. S.	Beauchamp
Nancy D.	Beaulieu
P.	Bebbington
Robert D.	Becher
Ulrike	Bechtold
Eleanor	Beck
Ingela	Beck
Ingela	Beck
Karin	Becke‐Jakob
Frank	Becker
Kimberly D.	Becker
Linda	Beckman
Mohamed	Bedaiwi
Roger	Beech
Emma	Beecham
Rebecca J.	Beeken
Katja	Beesdo‐Baum
Daniel	Beever
Christi	Befort
Rachna	Begh
Amir	Behboudi
Binod K.	Behera
Daiga	Behmane
Roozbeh	Behroozmand
Christoph P.	Beier
Ina	Beintner
Judith	Bek
Charlotte	Bekker
Hilary	Bekker
Mona	Bekkhus
H.	Beks
Saranda	Bekteshi
Tadele Biresaw	Belachew
Emmanuelle	Belanger
Lynda	Bélanger
Sumedh	Bele
Andrei	Beliaev
Jeffrey	Belkora
Diane	Bell
Catherine H.	Bell
Melanie L.	Bell
Robert	Bell
Sigall K.	Bell
Simon J.	Bell
Chyrell D.	Bellamy
S.	Bellass
Alessio	Bellato
Ruth	Belling
Julia	Bello‐Bravo
Nidhal	Belloumi
Zakaria	Belrhiti
Danai	Bem
Bart J. F. van den	Bemt
Xavier	Benarous
Pierina	Benavente
Rami	Benbenishty
Suzanne	Bench
S.	Bendall
Shelly	Ben‐David
Alexis A.	Bender
Arcelio	Benetoli
Enrique Garcia	Bengoechea
Kajsa	Bengtsson
Kristina	Bengtsson Boström
Helen	Benham
Sara	Benham
Vicente A.	Benites‐Zapata
Aprile D.	Benner
Brooke L.	Bennett
Clare	Bennett
Ian	Bennett
Kirsty	Bennett
Sally	Bennett
Tim	Benning
I. M.	Bensenor
Djamel	Bensmail
Madeleine	Benton
Cloe	Benz
Petra	Benzinger
Roberto P.	Benzo
John Jamir	Benzon R. Aruta
Idrissa	Beogo
David	Beran
Lori	Berard
Yana	Berardini
Gunnhild	Berdal
Tamás	Bereczky
Eva‐Maria	Berens
Bryony	Beresford
Bjorn	Berg
Inger Jorid	Berg
Karianne	Berg
Gabriele	Berg‐Beckhoff
Anna	Bergenheim
Sara	Berger
Zackary D.	Berger
Carolina	Bergerum
Johan	Berghmans
Alicia	Bergman
Aileen	Bergström
Jurg	Berhnard
Cady	Berkel
Alexandre	Berkesse
Danielle	Berkovic
Scott A.	Berkowitz
Seth A.	Berkowitz
Didier	Berlo van
Mathieu	Bernard
Charles N.	Bernstein
Meredith Root‐	Bernstein
Clara	Berridge
Clio	Berry
Katherine	Berry
Jay G.	Berry
Anna	Bershteyn
Mona	Berthelsen
Ann‐Sofie	Bertilsson
Lavinia	Bertini
Kathryn	Berzins
Romy	Bes
Stephanie	Beshara
Elodie	Besnier
Megan	Best
Robert	Best
Cornelia	Betsch
Cecily L.	Betz
Anna J.	Beurskens
Elizabeth A.	Beverly
Elizabeth A.	Beverly
Sea Matilda	Bez
Michael	Bezuhly
Diama	Bhadra Vale
Suki	Bhamra
Hemant	Bhargav
M.	Bhargav
Madhavi	Bhargava
Deepak	Bhatt
Debi	Bhattacharya
Siladitya	Bhattacharya
Kallol Kumar	Bhattacharyya
Anish	Bhuva
Alessia	Bianchi
Aida	Bianco
Jennifer	Bibb
Akhtar	Bibi
Stavroula	Bibila
Michele	Biddle
Julie T.	Bidwell
Mariola	Bidzan
Christiane	Bieber
Lucie	Biehler‐Gomez
Agnieszka	Bień
Mia	Bierbaum
Barbara	Biesecker
Christine	Bigby
Sarah	Bigi
Leon	Bijlmakers
Yaprak Ozum Unsal	Bilgin
Md Baki	Billah
Jo	Billings
Corey B.	Bills
Silvia	Bino
Peter	Binyaruka
Patricia	Biondo
Harry Tattan‐	Birch
Patricia	Birch
Marissa	Bird
Eva	Biringer
Søren	Birkeland
Hollie	Birkinshaw
Yvonne	Birks
Kathryn	Birnie
Kathryn A.	Birnie
Linda	Birt
Andrea	Bishop
Felicity	Bishop
Glenda M.	Bishop
Lauri	Bishop
Simon	Bishop
Marie	Bismark
Paul	Bissell
Mathieu	Bisson
Fernando Valentim	Bitencourt
Rebecca	Bitsko
Giulia	Bivona
Laura	Bix
M.	Bjarnadottir
Elisabeth Krefting	Bjelland
Stig	Bjønness
Thröstur	Björgvinsson
Anna	Björkdahl
Silje	Bjørnsen Haavaag
Aggie	Black
Georgia	Black
Melissa H.	Black
Michelle	Black
Steven	Blackburn
Steven	Blackburn
A. Marie	Blackmore
Rebecca	Blackwell
Amy	Blakemore
Amélie	Blanchet Garneau
David	Blane
Bob	Blankenberger
Amanda Jane	Blatch‐Jones
Lorraine	Blaxter
Charlotte	Blease
Nienke	Bleijenberg
Claire	Blewitt
Christian	Blickem
Christian	Blickem
Mette	Bliddal
B.	Blobel
Jeanett	Blom
Marie Inger	Blomberg
Ylva Thernström	Blomqvist
R.	Blood
Melissa J.	Bloomer
Jeffrey P.	Blount
Myra	Bluebond‐Langner
David L.	Blustein
Christopher C.	Blyth
Michelle D. S.	Boakye
Sheila A.	Boamah
Thomas	Boat
Marina	Boccardi
Virginia	Boccardi
Irina	Böckelmann
Janet	Boddy
Kate	Boddy
Christine	Bodemer
Catherine	Boden
Bo	Bodil
José Laerte	Boechat
Pauline	Boeckxstaens
Kasey	Boehmer
Anette	Boenick
Silke	Boenigk
Kathleen de	Boer
Marco	Boeri
Michael	Boettcher
Ronny	Boey
Dagje	Boeykens
Annick	Bogaerts
Laura	Bogart
Laura M.	Bogart
Ugo	Boggi
Søren Bie	Bogh
Marija	Bogic
Jennifer	Bogner
Cari A.	Bogulski
Bernt	Bøgvald Aarli
Antoine	Boivin
Jacky	Boivin
Chris	Bojke
Barbara	Bokhour
Angela	Boland
Fiona	Boland
Isabelle	Boland
L.	Boland
Laura	Boland
Francois V.	Bolduc
Marie	Boltz
Melanie	Boltzmann
Åse	Boman
Manja	Bomhoff
Hanna	Bomhof‐Roordink
Tanner	Bommersbach
Marcel	Bonay
Kathy S.	Bond
Jesper	Bonde
Loris	Bonetti
Inge	Bongers
Bruno	Bonnechère
Ann	Bonner
Sidra N.	Bonner
Emmanuel	Bonnet
Matthew	Booker
Annelies	Boonen
Fleur	Boot
Jonathan	Boote
Jonathan	Boote
Lizzy M. M.	Boots
Michael	Bording‐Jorgensen
Aleksandra	Borek
Johan	Borg
Francesca	Borgnis
Sander	Borgsteede
Sander	Borgsteede
Erica	Borgstrom
Josep M.	Borras
Carme	Borrell
Antonio	Borriello
Anna E.	Bortnick
J.	Bosak
Kate	Bosanquet
Veronique	Boscart
Jackie	Bosch
Cristina	Bosch‐Farré
Rachel	Boscott
Kobie	Boshoff
R. C.	Bosman
Sinthia	Bosnic‐anticevich
Maarten J. A. Van Den	Bossche
Jean‐Luc	Bosson
Jennifer	Bostock
Mari	Botti
Joan	Bottorff
Frederick	Bouckaert
Anastasia	Bougea
Kathryn	Bould
Maged N. Kamel	Boulos
Kelsie	Boulton
Anne Sophie	Boureau
Stéphanie	Bourion‐Bédès
Helen	Bourke‐Taylor
Claude Julie	Bourque
Céline	Bourquin
Maya Mroué	Boustani
Marie‐Ève	Bouthillier
Renée	Bouwman
Hylco	Bouwstra
Riley M.	Bove
Nicholas	Bowden
Audrey	Bowen
Liza	Bowen
Sarah	Bowen
Kate	Bower
Peter	Bower
Hannah	Bowers
Lucy	Bowes
Jessamyn	Bowling
Bryher	Bowness
Alan	Boyd
Rhonda	Boyd
Cherrie B.	Boyer
Mark E.	Boyes
Anne‐Marie	Boylan
Paul	Boyle
Chloe	Brabon
Massimo	Bracci
Dana Elisabeth	Brackney
Zoe	Bradfield
Eleanor	Bradley
Fay	Bradley
Hannah	Bradwell
Louca‐Mai	Brady
Marian	Brady
Bernadette	Brady
Line	Bragstad
Line Kildal	Bragstad
Katherine	Brain
Jeffrey	Braithwaite
Jeffrey	Braithwaite
Kate	Bramham
Manuel Castelo	Branco
Gabrielle	Brand
Paul L. P.	Brand
Megan	Branda
Marina de Brito	Brandão
Daan	Brandenbarg
Joline Elizabeth	Brandenburg
Helene	Brandon
Jenny	Brands
Alexandra	Brandt Ryborg Jønsson
Susan	Brandzel
Emer	Brangan
Robert E.	Brannigan
Sophie	Brassel
Anna Sofia	Bratt
Ewa‐Lena	Bratt
Ola	Bratt
Sandra	Brauer
Annette	Braunack‐Mayer
Marie Ernsth	Bravell
Alison	Bravington
Adrian J.	Bravo
Jesús	Bravo
Lucy	Bray
Debbie	Braybrook
Carol	Brayne
Anne‐Sophie	Brazeau
Sally	Brearley
Sarah	Brearley
Alan	Breen
Liz	Breen
Cathy	Breen
Caterina	Breitenstein
Jessica Y.	Breland
Audun	Brendbekken
Sydney	Breneol
Nicola	Brennan
Christopher Patrick	Bretherton
Jo	Brett
Jo	Brett
Erica	Breuer
Karen	Brewer
LaPrincess C.	Brewer
Violeta	Briciu
Leslie Ann Daline	Brick
Rachelle	Brick
Katie Jane	Brickwood
Margarita	Brida
Mala	Bridgelal Ram
Bernadetta	Bridgwood
Jo‐anne	Brien
Matthew	Brier
Andrew	Briggs
Farren B. S.	Briggs
Michelle	Briggs
Diana	Bright
Felicity A. S.	Bright
Kristin L.	Bright
Lisa	Brighton
Sarah Marie	Briké
Lucy	Brindle
Erica	Briones‐Vozmediano
Katie	Brittain
James	Britton
Flemming	Bro
Simon A.	Broadley
Lotte	Broberg
Mathias	Brochhausen
Paula M.	Brochu
Teresa	Brockie
Henry	Brodaty
Lucia	Brodosi
Anne	Brødsgaard
Martin B.	Brodsky
Amy	Brodtmann
M. J. M.	Broeders
Birit F. P.	Broekman
Jacqueline	Broerse
Mariske	Broersen
Elizabeth	Brogan
Stina	Brogard Andersen
Emma Louise	Broglia
Elaine	Brohan
Linda	Brom
Collette	Bromhead
Joanne	Brooke
Nicholas	Brooke
Dawn	Brooker
Helen	Brooks
Helen	Brooks
Ronald A.	Brooks
Dina	Brooks
Jane	Brooks
Ashley	Brooks‐Russell
Lisa M.	Brophy
Sandra	Brouwer
Therese	Brovold
Adrian	Brown
Alex	Brown
Carl	Brown
Edwina (Prof.)	Brown
Eleanor	Brown
Helen	Brown
Judith	Brown
Kevin D.	Brown
Lana	Brown
Laurin	Brown
Lydia	Brown
Maria T.	Brown
Marion	Brown
Richard	Brown
Richard	Brown
Stephanie J.	Brown
Louis D.	Brown
Annette	Browne
Felicia	Browne
Sharon	Brownie
Colette	Browning
Laura Jane	Brubacher
Emma	Bruce
Iain	Bruce
Anna L.	Bruckner
Thierry	Brue
Anna Levke	Bruett
Ruairi	Brugha
Eling D. de	Bruin
Melissa	Brunner
Crystal	Brunoro
Alfredo	Brusco
Natasha K.	Brusco
Mariana	Brussoni
Anna	Brütt
Anna Levke	Brütt
Jon Ram	Bruun‐Pedersen
Jean‐marie	Bruzzese
Joanne	Bryant
Louise	Bryant
Denise	Bryant‐Lukosius
Shayden	Bryce
Patrick	Brzoska
Sandra	Bucci
Francine	Buchanan
Rachelle	Buchbinder
Daniel Z.	Buchman
Jessica	Bucholc
Benjamin	Buck
Elizabeth Taylor	Buck
Harleah G.	Buck
Elizabeth	Buckley
Tracey	Bucknall
Timothy	Budden
Mia	Budescu
Monika	Budnik
Aurora	Bueno‐Cavanillas
Carol	Bugge
Franklin Chu	Buh
Anthony	Buisson
Diana	Buist
Anne	Bukten
Özlem	Bulantekin Düzalan
Claudia	Bull
Laurna	Bullock
Joske	Bunders
Frances	Bunn
Massimiliano	Buoli
Danilo	Buonsenso
Jennie	Burch
Sorrel	Burden
Teresa	Burdett
Julia G.	Burgdorf
Fred	Burge
Caroline	Burgess
Diana	Burgess
Rochelle	Burgess
Nils	Bürgisser
Gerd	Burmester
Anne‐Marie	Burn
Karen	Burnell
Michel	Burnier
Lorna	Burns
Alison	Burrows
Christine	Burrows
Julia	Burrows
Kristen	Burrows
Brian A.	Burt
Jenni	Burt
Jenni	Burt
Alexandra	Burton
Christopher	Burton
David	Burton
Wendy	Burton
Johannes	Burtscher
Errol L.	Bush
Matthew L.	Bush
Doreen	Busingye
Maaike	Buskermolen
Jan J. V.	Busschbach
Monica	Busse
Marta	Buszewicz
Nancy	Butcher
Rebecca	Butcher
Isabelle	Butcher
Javed	Butler
Stacey	Butler
Phyllis	Butow
Jack	Butterworth
Lyndsey	Butterworth
Sandra C.	Buttigieg
Bianca	Buurman
Bianca M.	Buurman
Samuel	Byiringiro
Richard	Byng
Richard	Byng
Elaine	Byrne
Margaret	Byrne
Molly	Byrne
Lucie	Byrne‐Davis
Diana	C. Sanchez Ramirez
Barbara	C. Schouten
Jennifer	C. Spencer
Elizabeth	C. Thomas
Nuria	Caballol
María J.	Cabero‐Perez
Sarah	Cable
Lauren	Cadel
Cristina	Cadenas‐Sanchez
Jennifer M.	Cadigan
Cathal A.	Cadogan
Ana	Caetano
Eilidh	Cage
Serena	Caggiano
Annachiara	Cagnin
Suzanne	Cahill
W.	Cahn
Zenglin	Cai
Joanne	Cairns
Cuma	Çakmak
Rocco Salvatore	Calabrò
Rachel	Calam
Susan	Calcaterra
Amaia	Calderón‐Larrañaga
Sara	Calderón‐Larrañaga
Lisa	Caldon
Patrina	Caldwell
Alison L.	Calear
Timothy	Callaghan
James M.	Callahan
Aileen	Callanan
Emily	Callander
Felicity	Callard
Felicity	Callard
Libby	Callaway
Joanne	Callen
Emily	Callendar
Michele L.	Callisaya
Sergio	Calonge‐Pascual
Melanie	Calvert
Xavier	Calvet
Miryam	Calvino
Rafael	Calvo
Maria Elena	Camacho‐Moll
David	Cámara‐Liebana
Celiane	Camargo Borges
Wendy	Camello‐Castillo
I. D.	Cameron
Jan	Cameron
Jill	Cameron
Joan	Cameron
Michelle H.	Cameron
Olivia	Cameron
Rachel	Cameron
Christine	Campbell
David	Campbell
John	Campbell
Letitia	Campbell
Megan	Campbell
Paul	Campbell
Stephen	Campbell
Marsha	Campbell‐Yeo
Zühal	Çamur
Çağdaş	Can
Eugenia	Canas
Dion	Candelaria
Pedro	Candol
Carey	Candrian
Bridget	Candy
J. P.	Caneiro
Ismelda	Canelo
Oliver J.	Canfell
Melissa	Cannon
Anne	Canny
Naomi	Cano‐Ibáñez
Robyn	Cant
Ryan S.	Cantor
Maureen	Cantwell
Karla	Canuto
Krysia	Canvin
Miriam	Capasso
Cristina	Caperchione
Lizzie	Caperon
Susan	Caplan
Lora	Capobianco
Andrea	Capstick
Enrico	Capuzzi
Kristine	Carandang
Sara	Carazo
Cristina Fernández	Carballido
Carina	Carbia
Alan	Card
Nicole	Cardenas
Magnolia	Cardona
Magnolia	Cardona
Emma	Carduff
Ann‐Louise	Caress
Melissa	Carey
James	Carifio
Fabrizio	Carinci
Misericordia	Carles‐Lavila
Kathleen	Carlson
Samantha	Carlson
Emilia	Carlsson
Eva	Carlsson
Tommy	Carlsson
Jill	Carlton
Kathleen	Carluzzo
Kellie	Carlyle
Kristin	Carman
Rebekah	Carney
Mary	Carolan‐Olah
Sarah	Caron
J. D.	Carpentieri
Sarah	Carr
Sarah	Carr
Tracey	Carr
Amanda	Carroll
Pádraig	Carroll
Gunilla	Carson
Nicholas	Carson
Andrew	Carson‐Stevens
Andrew	Carson‐Stevens
Louise	Carstam
Claire	Carswell
Adrian	Carter
Ben	Carter
Bernie	Carter
Bronwyn	Carter
Chris	Carter
David J.	Carter
Jessica	Carter
Julia	Carter
Melissa T.	Carter
Pam	Carter
Stacy	Carter
Tim	Carter
Vanessa	Carter
Gillian	Carter
Lisa	Carter‐Bawa
Lisa	Carter‐Harris
Kathleen B.	Cartmell
Giulia	Cartocci
Anto	Čartolovni
Francisco	Cartujano‐Barrera
Kate	Cartwright
Natasha	Carver
Georgia	Casanova
Siobhan	Case
Marta	Caserotti
Dympna	Casey
Lucinda	Cash‐Gibson
Aidan G.	Cashin
Daniela	Caso
Marica	Cassarino
Annie	Cassidy
Christine	Cassidy
Irene	Cassidy
Riccardo	Castagnoli
Sven	Casteleyn
Ursula	Castellano
Yolanda	Castellote‐Caballero
Xavier	Castells
Charlotte	Castor
Carolina Moreno	Castro
Gloria Fernanda	Castro
Maria L.	Castro‐Codesal
Enrique	Castro‐Sanchez
Glenys	Caswell
Gianluca	Catania
Soraia de Camargo	Catapan
Pascal	Cathebras
Duclos	Catherine
Davide	Cattaneo
Alfredo	Caturano
Afonso	Cavaco
Sabrina	Cavallo
Silvia	Cavedoni
Debbie	Cavers
Vladimíra Kurincová	Čavojová
C. Susana	Caxaj
Yasemin	Çayır
Lucía	Cea‐Soriano
Filippo	Ceccato
Christine	Cedraschi
Luca	Cegolon
Ivette	Cejas
Loredana	Cena
Jose G.	Centeno
Piera	Centobelli
Alison	Cerezo
Paige E.	Cervantes
Flaviane Cristina Rocha	Cesar
Carolyn E.	Cesta
Marisa	Cevasco
Muge	Cevik
EunSeok	Cha
Seungman	Cha
Wendy	Chaboyer
Anil	Chacko
Neil	Chadborn
Sena	Chae
Consuelo	Chafer‐Pericas
Benjamin W.	Chaffee
Nujjaree	Chaimongkol
Rhyddhi	Chakraborty
Hom Nath	Chalise
Sheetal	Challam
Iain	Chalmers
John	Chamberlain
Christine T.	Chambers
Derek	Chambers
Lucy	Chambers
Jane Dimmitt	Champion
Carmen W. H.	Chan
Christian S.	Chan
Esther	Chan
Helen Yue‐lai	Chan
Kam Wa	Chan
Miranda	Chan
Randolph C. H.	Chan
Raymond	Chan
Teresa M.	Chan
Vincy	Chan
Gary Chi Wang	Chan
Loretta Yuk Yee	Chan
Rahul	Chanchlani
Joht Singh	Chandan
Andrew	Chanen
B. P.	Chang
Won‐Du	Chang
Yan‐Shing	Chang
Khatidja	Chantler
Emma	Chaplin
Cath	Chapman
Derek A.	Chapman
Liz	Charalambous
Divya A.	Chari
Georgina	Charlesworth
Olga	Charnaya
Margaret	Charnley
Sirirat	Charuvanij
Anita	Chary
Jo‐Ana Dolojan	Chase
Guillaume	Chassagnon
Eleanor	Chatburn
Antoinette	Chateau
Angel	Chater
Rebecca E.	Chatham
Arunangsu	Chatterjee
Nikhil	Chaudhary
Swapna	Chaudhary
Atanu	Chaudhuri
Amrit	Chauhan
Nicolas	Chausson
Jorge E.	Chavarro
Elisa M.	Chávez
Ewelina	Chawłowska
Beibei	Che
Nancy	Cheak‐Zamora
Perla	Chebli
Kath	Checkland
Michael	Chee
Kathleen	Chell
Diane	Chen
Gang	Chen
Hong	Chen
Hong	Chen
Jie	Chen
Jun	Chen
Lei‐Shih	Chen
Pei‐Chun	Chen
Qirong	Chen
Ren	Chen
Timothy	Chen
Timothy	Chen
Yi Chun	Chen
Chuanpin	Chen
Anais	Cheneau
Abby L.	Cheng
Christina	Cheng
Ho Yu	Cheng
K. K.	Cheng
Kimmy	Cheng
Mei‐Yun	Cheng
Lyn	Chenoweth
Ai Theng	Cheong
Lynn	Cheong
Sudeh	Cheraghi‐Sohi
Gemma	Cherry
Antonio	Cherubini
Helen	Chester
Annie Wai Ling	Cheung
Daphne	Cheung
Janet	Cheung
Yin Ting	Cheung
Boris	Cheval
Bernadette	Chevalier
Boon How	Chew
Carolyn	Chew‐Graham
Betty	Chewning
Sumedha	Chhatre
Nai‐Fang	Chi
Stefania	Chiappinotto
Zatelli Maria	Chiara
Ilaria	Chiarantini
Francesco G.	Chiarelli
Lisa A.	Chiarello
Davide	Chiasserini
Maria	Chicco
Claudia	Chien
Ahizechukwu	Chigoziem Eke
Marshall H.	Chin
Jane Yuet	Ching Hui
Karuthan	Chinna
Kathy S.	Chiou
Michael G.	Chipeta
Jennifer	Chipps
Francesco	Chirico
Ilaria	Chirico
Anna	Chisholm
Katrina	Chisholm
Shilpa	Chitnis
Meredith L.	Chivers
NaiRui	Chng
Hwayoung	Cho
Mildred	Cho
Hyung J.	Cho
Young June	Choe
Chulhwan	Choi
Jeeyae	Choi
Seul Ki	Choi
Mumbi	Chola
Satheesh	Chonat
Patty	Chondros
Yuen Yu	Chong
Meck	Chongo
Tana	Chongsuwat
Stephen R.	Chorney
Marta	Choroszewicz
Aileen	Chou
Tanzeem	Choudhury
Maud‐Christine	Chouinard
Daniela	Choukair
Amine	Choukou
Amy Y. M.	Chow
Nashit	Chowdhury
Mimi	Choy‐Brown
Eva	Christalle
Alan J.	Christensen
Hannah	Christensen
Kaj Sparle	Christensen
Floor	Christie‐de Jong
Christmal	Christmals
Danielle Ni	Chroinin
Catherine	Chronaki
Kia‐Chong	Chua
Wei Ling	Chua
Emmeline	Chuang
Anna	Chudyk
Carla D.	Chugani
Ivy	Chumo
Holly	Chung
Il Yong	Chung
Jane	Chung
Soondool	Chung
Vincent	Chung
Yoongi	Chung
John	Church
Alasdair	Churchard
Kate	Churruca
Linda C.	Chyr
Luisella	Cianferotti
Maria Vincenza	Ciasullo
Marco	Cicciù
Giancarlo	Cicolini
Pierangelo	Cifelli
Necmettin	Çiftci
Dilek	Cilingir
Sarah	Ciotti
Sabrina	Cipolletta
Camille	Cipri
Francesca	Cirulli
Kumaresan	Cithambarm
Jurgen A. H. R.	Claassen
Lauren	Clack
Valerie A. Wright‐St	Clair
Creutzfeldt	Claire J.
Kirsty	Clark
Michael	Clark
Pat	Clark
Terryann C.	Clark
Aileen	Clarke
David	Clarke
David B.	Clarke
E.	Clarke
Emma	Clarke‐Deelder
Corinne	Clarkson
Gina	Clarkson
Jan	Clarkson
Ana	Cláudia Mesquita Garcia
Elizabeth B.	Claus
Kevin	Clauson
Angelika H.	Claussen
Nathalie	Clavel
Kate	Claydon‐Platt
Marla	Clayman
Marla	Clayman
Dora L.	Clayton‐Jones
Bryan	Cleal
Sheila M.	Clemens
Michel	Clement
Vicente Javier	Clemente‐Suárez
Mark	Clemons
Jerome	Cleofas
Rosemary	Clerehan
Kristin	Cleverley
Ash Kieran	Clift
Carolina	Climent‐Sanz
Victoria	Cluley
Victoria	Cluley
Donna	Clutterbuck
Jane	Coad
Kathryn E.	Coakley
Laura	Coates
Cory L.	Cobb
Julien	Cobert
Lorenzo	Cobianchi
Karen	Cochrane
Emma	Cockcroft
Emma	Cockcroft
Yvonne	Codd
Jillianne	Code
Norma B.	Coe
Adriana	Coelho
Carl	Coelho
Vera	Coelho
Jessica	Coffing
David R.	Coghill
David	Coghlan
Alison K.	Cohen
Joachim	Cohen
Enrico	Coiera
Nananda F.	Col
Alistair	Cole
Helen	Cole
Marco	Colizzi
Ryan J.	Coller
Johnny	Collett
Tracey	Collett
Aileen	Collier
Aileen	Collier
Philippa	Collin
Claire	Collins
Clare E.	Collins
Griffin S.	Collins
Jack	Collins
Jemma	Collins
Linda	Collins
T.	Collins
Tea	Collins
Cinzia	Colombo
Sara	Colomer‐Lahiguera
Cristina	Colon‐Semenza
Heather	Colquhoun
Erminia	Colucci
Josielli	Comachio
Adelina	Comas
Emilie	Combet
David	Comerford
Persis V.	Commissariat
Dominique	Como
Matteo Monzio	Compagnoni
Bruce	Compas
Lee Ann E.	Conard
Bin	Cong
Mairead	Conneely
Louise	Connell
Bradley T.	Conner
Barbara	Conner‐Spady
Amy K.	Connery
Dean	Connolly
Michael	Connolly
Charlotte	Connor
Paul O.	Connor
Stephen	Connor
Susan Jane	Connor
Elizabeth	Conroy
Tiffany	Conroy
Nathan	Consedine
Francesca	Conserva
Monica	Consolandi
John	Constantino
Constantina	Constantinou
Kathleen	Conte
Edgar Carnero	Contentti
Elisa	Conti
Richard	Contrada
David I.	Conway
Soledad	Coo
Chad	Cook
Jill	Cook
Loraine	Cook
Matthew	Cook
Tina	Cook
Heather A.	Cooke
Alexis	Cooke
Debbie	Cooke
Deborah	Cooke
Mary	Cooke
Mary	Cooke
Rachel	Cooney
Kate	Cooper
Kay	Cooper
Silvie	Cooper
Ruth E.	Cooper
Sherva Elizabeth	Cooray
Genevieve	Coorey
Susan	Coote
Hannah	Copeland
Lauren	Copeland
Jodie	Copley
Kirsten	Coppell
Michel	Coppieters
Ilaria	Coppola
Francesco	Corallo
Ilaria	Corazza
Angus	Corbett
Teresa	Corbett
Marc	Corbiere
Giselle	Corbie‐Smith
Rhiannon	Corcoran
Lis	Cordingley
Charlie	Corke
Evelyn	Cornelissen
Michelle	Cornes
Nadia	Corp
Helen	Correia
Kendall	Cortelyou
Catherine	Cosgrave
Sylvie	Cossette
Joao	Costa
Cristiane Maria Carvalho	Costa Dias
Anna	Costantini
Mary	Costanza
Leesa	Costello
Joanne	Coster
Denise	Côté‐Arsenault
Kristien	Coteur
Valerie T.	Cotter
Sarah	Cotterill
Seonaidh	Cotton
Elizabeth	Cottrell
Simon	Cottrell
William	Cottrell
Rachel	Couban
Helen	Coughlan
William	Couldwell
Drew	Coulson
Angela	Coulter
Juul M. J.	Coumans
Chelsea	Coumoundouros
Nia	Coupe
Nia	Coupe
Pierrick	Coupé
Darren	Courtney
Mary	Courtney
Suzanne E.	Courtwright
Melissa K.	Cousino
Sian	Cousins
Catherine	Coveney
Peter	Coventry
Fiona	Cowdell
Luke	Cowie
Caitríona	Cox
Christopher E.	Cox
Narelle S.	Cox
Ruth	Cox
Liza	Coyer
Liza	Coyer
Claire A.	Coyne
Imelda	Coyne
Stefano	Crabu
Ian	Craddock
Åsa	Craftman
Adam T.	Craig
Simon	Craig
Brooke	Craik
Melinda	Craike
Cathleen	Crain
Jane Murray	Cramm
Fiona	Cramp
Kathleen A.	Crapanzano
Michael	Craven
David	Crawford
Sharinne	Crawford
Alison	Crawshaw
Amy V.	Creaser
Nicole	Creasey
Ann‐Marie	Creaven
Bruce A. C.	Cree
Genevieve	Creighton
Pascal	Crépey
Cathy	Creswell
Alan	Cribb
Joanna	Crocker
Laura D.	Crocker
Katie	Crockett
Nicholas M.	Croft
Anne de la	Croix
Neeltje	Crombag
Sara J.	Cromer
Catherine	Crompton
Peter F.	Cronholm
Ashley	Crook
Liz	Croot
Ann	Crosland
Emily	Cross
Jane	Cross
Lisa	Crossland
Norah L.	Crossnohere
Maria	Crotty
Rebecca	Crowder
Rebecca	Crowder
Brandi	Crowe
Sally	Crowe
Angela A.	Crowley
Cynthia S.	Crowson
Damian	Cruse
Rik	Crutzen
Jonas Preposi	Cruz
Péter	Csécsei
Herbert	Cubasch
Oliver	Cuddeford
Ebenezer	Cudjoe
Alison E.	Cuellar
Jialiang	Cui
Yu	Cui
Danielle	Cullen
Patricia	Cullen
Olga	Cunha
Amy T.	Cunningham
Michael R.	Cunningham
David	Cunningham
Wayne	Cunningham
Emile	Curfs
Alexandra	Curran
Janet	Curran
Candace	Currie
Gillian	Currie
Jane	Currie
K.	Currie
Sinead	Currie
Jennifer	Currin‐McCulloch
Cate	Curtis
Jackie	Curtis
P.	Curtis
Katherine	Curtis‐Tyler
Kenneth	Cusi
Carlo	Custodero
Natalie	Cutler
Maarten	Cuypers
Rachael	Cvejic
Biljana	Cvetkovski
Jonathan	Cylus
Justyna	Czekajewska
Iwona	Czerska
Wladyslawa	Czuber‐Dochan
Matt	D.
Jared	D. Huling
Patrick	D. McGorry
Cameron	D. Norman
Jonathan	D. Santoro
Peter	D. Sasieni
James	D. Stefaniak
Ruben	D. Vromans
M. Pinto	da Costa
Kevin	Dadaczynski
Ann	Dadich
Payam	Dadvand
Maud	Daemen
Morgan L.	Daffin
Amrita	Daftary
Christian	Dagenais
Bente	Dahl
Karina	Dahl Steffensen
Hannah G.	Dahlen
Maria	Dahm
Maria R.	Dahm
Alina	Dahmen
André	Dailey
Luke	Daines
Francesca	Dal Mas
Knut Eirik	Dalene
Christine	Daley
Kim	Daley
Stephanie	Daley
Nicole K.	Dalmer
Jen	Dalton
Deirdre	Daly
Sheila	Daly
Timothy	Daly
Jean	Daly‐Lynn
Pieter J. Van	Dam
Olga	Damman
Olga C.	Damman
Kristen	Dams‐O'Connor
Natalya	Danchenko
Katrien	Danhieux
Kondziella	Daniel
Ojuka	Daniel
Jane	Daniels
Michelle	Dannenberg
Michelle	Dannenberg
Rosana	Dantas
Bianca	D'Antono
Melissa Dominice	Dao
Ansaam	Daoud
Marjan	Darabi
Alessia	d'Arma
Karleigh	Darnay
Blair G.	Darney
Francine	Darroch
Sirwan	Darweesh
Risqa Rina	Darwita
Indranil	Dasgupta
Sharoda	Dasgupta
Lise	Dassieu
Ana M.	Daugherty
Sarah E.	Daugherty
Barbara	D'Avanzo
Gaurav	Dave
Gaurav	Dave
Margie H.	Davenport
Janet	Davey
Chiara	Davico
Ana de	David
Kiefer	David
Jennie	David
Berlowitz	David J.
Alexandra	Davidson
Larry	Davidson
Patricia M.	Davidson
Anna	Davies
Clare	Davies
Elizabeth	Davies
Emma L.	Davies
Freya	Davies
Hugh	Davies
James	Davies
Julia	Davies
Luke	Davies
Myfanwy	Davies
Nathan	Davies
Ellen	Davies
Guadalupe	Dávila
Deborah	Davis
Gershan	Davis
Maryann	Davis
Mellar	Davis
Stacy	Davis
Carol	Davy
Zowie	Davy
Jo	Dawes
Nathan	Dawes
Piers	Dawes
Piers	Dawes
Amy	Dawson
Pauline	Dawson
Shoba	Dawson
Shoba	Dawson
Suzanne	Dawson
Angela	Dawson
Enya	Daynes
Chris	Dayson
Carolijn	de Beer
Esther	de Bekker‐Grob
Domenico	De Berardis
Edwin	de Beurs
Filipa	De Castro
Maria Cristina	De Cola
David	De Coninck
Werner	de Cruppé
Andre Schutzer	de Godoy
Hans	de Graaf
Judith	De Jong
Lieke	de Jong
Margriet F. C.	de Jong
Loes	de Kleijn
Johannes Hendrikus	De Kock
Inge M. C. M.	de Kok
Carlos	De las Cuevas
Melanie	de Looper
Katie	De Luca
Janneke	De Man‐van Ginkel
Maddalena	De Maria
Carmen	de Mendoza
Sandrine	de Montgolfier
Érica Brandão	de Morae
Simone	De Morgan
Jascha	de Nooijer
Ana	de Oliveira Guedes Bastos
Ludovica	De Panfilis
Chiara	De Poli
Sabina	De Rosis
Anniek	de Ruijter
Silvia	de Sanjosé
Francesco	De Seta
Anna	De Simoni
Ísis	de Siqueira Silva
Helena	De Sola
Oriol	De Sola‐Morales
Luis Manuel Mota	de Sousa
Ricardo Rodrigues	de Sousa Junior
Edige Felipe	de Sousa Santos
Ana Claudia	De Souza
Savia	de Souza
Marian	de van der Schueren
Anke J. E.	de Veer
Aline	de Vleminck
Frank	de Vocht
Kay	de Vries
Marieke	de Vries
Ardine	de Wit
John B. F.	de Wit
Kirk	Dearden
Shoumitro	Deb
Malden	Debbie E.
Héloïse	Debelle
Deborah	Debono
Matthew	DeCamp
Kalli B.	Decker
E.	Declercq
Danielle D.	DeCourcey
Christine	Dedding
Christine	Dedding
Gayle	Dede
Edward Christopher	Dee
Rita	Deering
Athavudh	Deesomchok
Chris	Degeling
Howard B.	Degenholtz
Pam B.	DeGuzman
Ali	Dehghani
Mats	Dehlin
Gus A.	Dekker
Tycho J.	Dekkers
Bruna	Del Vechio Koike
Alexandre	Delamou
Rebecca K.	Delaney
Julie M.	Deleemans
Luigi	Della Corte
Christine	Delon
Isabel‐Cathérine	Demel
Yasemin	Demir Avcı
Laura	Dempsey
Martin	Dempster
Stephan Van	den Broucke
Audrey‐Ann	Deneault
Sarah	Denford
Karen Harrison	Dening
Harrison	Denise
Semiha	Denktaş
Elizabeth	Denney‐Wilson
Tom	Denning
Sarah	Dennis
Rebecca	Dennison
Elaine	Denny
Konstancja	Densley
Christophe E.	Depuydt
Ufuk	Derinsu
Elvira	Derks
Claudia	Der‐Martirosian
Michael R.	DeRoode
Wayne	DeRuiter
Nimisha	Desai
Jane	Desborough
Mieke	Deschodt
Jonathan	DeShazo
Ashish A.	Deshmukh
Lorenzoi	Desider
Malini	DeSilva
Jules	Desmeules
Alethea	Desrosiers
Michel	Desy
Urshla	Devalia
Hemakumar	Devan
Niamh	Devane
Grazia	Devigili
Tracey J.	Devonport
Lindsay	Dewa
Maya	Dewan
Rachel	Dewar Haggart
Klodian	Dhana
Cecilia	Dhejne
Haryana	Dhillon
Teerapon	Dhippayom
Teerapon	Dhippayom
Claudio	Di Lorito
Clarissa Jonas	Diamantidis
Lisa C.	Diamond
Sonia	Dias
Ana	Diaz
Esperanza	Diaz
Marissa	Diaz
Andrea Duarte	Díaz
Lorena	Díaz De León‐Martínez
Miguel	Diaz‐Hernandez
Unai	Diaz‐Orueta
Cesar	Díaz‐Torné
Melissa	Dicarlo
Geoffrey L.	Dickens
Marissa	Dickins
Marissa	Dickins
Fiona M.	Dickinson
Angela	Dickinson
Rebecca	Dickinson
Annika	Diekmann
Lara	Diem
Kimberly	Dienes
Sigrid	Dierickx
Marie‐Luise	Dierks
Janan	Dietrich
Aimee	Dietz
R.	Digby
Michelle	DiGiacomo
Marina	Digregorio
Lisa	Dikomitis
Lisa	Dikomitis
Elise‐Marie	Dilger
Piyameth	Dilokthornsakul
Sandra	Diminic
Paul	Dimitri
Gina	Dimitropoulos
Ruoxi	Ding
James	Dionne‐Odom
Ndeye Thiab	Diouf
Carmen	Dirksen
Jörg	Dirmaier
Catherine	Diskin
Nicola	Diviani
Claire	Dix
Emma	Dixon
Jeremy	Dixon
Karen	Dixon
Katie	Dixon
Sharon	Dixon
Jafar Yahyavi	Dizaj
Abolghassem	Djazayery
Martin	Dlouhý
Sergiy	Dmytriyev
Christina	Dobson
Rosie	Dobson
Ruth	Dobson
Angie	Docherty
Alyson	Dodd
Helen	Dodd
Rachael	Dodd
Warren	Dodd
Robin L.	Dodds
Phidelia Theresa	Doegah
Susanne	Doepfmer
Omara	Dogar
Maman Joyce	Dogba
Anne M.	Doherty
Irene A.	Doherty
Megan	Doherty
Eva	Dohnalova
James C.	Doidge
Rowena	Dolor
Geert	Dom
Jan	Domaradzki
Stephan	Dombrowski
Caitlyn	Donaldson
Candice D.	Donaldson
Ascensión	Doñate‐Martínez
Sara	Donetto
Dong	Dong
Joseph	Donia
Valeria	Donisi
Jennifer R.	Donnan
Warren J.	Donnellan
Grainne	Donohue
Caroline	Donovan
Jenny	Donovan
Bonny	Donzella
O.	Doody
Keren	Dopelt
Paul	Dorfman
Andrea S.	Doria
Thomas E.	Dorner
Sam	Dorney‐Smith
Karoline	Doser
Leila	Doshmangir
Shelley	Doucet
Debbie	Dougherty
Flora	Douglas
Jacinta M.	Douglas
Linda	Douma
Michaël	Doumen
Eduardo	Dourado
Antonios	Douros
Robyn	Dowlen
James	Downar
Soo	Downe
Anna	Dowrick
Christopher	Dowrick
Ros	Dowse
Matt	Drabenstott
Mari‐Lynn	Drainoni
Anne	Drapkin Lyerly
Laynie	Dratch
Renate	Drechsler
Stefanie	Dreger
Vari	Drennan
Janet	Drew
Jessica	Drinkwater
Jessica	Drinkwater
Nina F.	Dronkers
Constance	Drossaert
Jenny	Drott
Aaron	Drovandi
Murray J. N.	Drummond
Amanda	Drury
Andrews Adjei	Druye
Nick	Drydakis
Melba Sheila	D'Souza
Esther	du Pon
Sanetta	du Toit
Tarun	Dua
Katarzyna	Dubas‐Jakóbczyk
Mark	Dubbelman
Paul	Duberstein
Veljko	Dubljevic
Nancy	Dudley
Jane	Duff
Mel	Duffy
Leah Stein	Duker
Vadim	Dukhanin
Simone V.	Dulmen
Elise	Dumas
Mari	Dumbaugh
Eilidh	Duncan
Polly	Duncan
Tinashe	Dune
Margaret	Dunham
Kate	Dunlop
Amy	Dunn
Jennifer	Dunn
Kate	Dunn
Simon	Dunne
Shira	Dunsiger
Jolan	Dupont
Veerle	Duprez
Mona	Dür
Mirko	Duradoni
Marie‐Anne	Durand
Marie‐Anne	Durand
Angela	Durante
Emma	Dures
Angela	Durey
Jo	Durham
Christine	Durif‐Bruckert
Matthew S.	Durstenfeld
Raika	Durusoy
Heidi	Dutton
Ülkü Saygılı	Düzova
Sarah	Dwinger
Andrew A.	Dwyer
Debra S.	Dyer
T. A.	Dyer
W. Justin	Dyer
Judith	Dyson
Krysia	Dziedzic
Margaret O'Hara	E.
Sarah	E. Hughes
Sarah	E. Rosenbaum
Kong	E. H.
Michelle J.	Eady
Valsamma	Eapen
Frances	Early
Julie	Easley
Daniela	Eassey
Christine E.	East
Phyllis	Easton
Narelle	Eather
Georgette	Eaton
Abbas	Ebadi
Judith	Eberhardt
David	Ebert
Jan M.	Eberth
Zahra	Ebrahimi
Azadeh	Ebrahimi‐Madiseh
Abi	Eccles
Claire	Eccleston
Eyyup	Ecevit
Micahell	Echteld
Mark	Eckman
Ayako	Edahiro
Julian	Edbrooke‐Childs
Regina	Eddie
Moses Onyemaechi	Ede
Alexandra	Edelman
Natalie	Edelman
Hilary	Edelstein
Offer Emanuel	Edelstein
Milton	Eder
Dawn	Edge
Alexandra	Edinger
Erandie	Ediriweera
Sarah M.	Edney
Matías	Eduardo Díaz Crescitelli
Adrian	Edwards
Katie	Edwards
Michelle	Edwards
Zoe	Edwards
Nicholas	Edwardson
Fisher	Edwin
Christantie	Effendy
Areti	Efthymiou
Richard	Egan
Leonard	Egede
Sandra K.	Eggenberger
Matthew J.	Ehrhardt
Frederic	Ehrler
Manuel	Eichenlaub
Michael	Eichinger
Trine Tetlie	Eik‐Nes
Rochelle	Einboden
Gunnar	Einvik
Andreas	Eisingerich
Sarah	Eitze
Dr. Ermias	Ejara
Stuart	Ekberg
Winifred	Ekezie
Inger	Ekman
Mirjam	Ekstedt
Charbel	El Bcheraoui
Suzanne	El Khechen
Faris	El‐Dahiyat
Sarira	El‐Den
Ann Catrine	Eldh
Ann Catrine	Eldh
Phoebe	Elers
Marie	Elf
Kristin	Elgersma
Ahmed	Elhakeem
Jaklin	Eliott
Mark	Elisha
Kathy	Eljiz
Safa	Elkefi
Saskia	Elkhalii‐Wilhelm
Katherine S.	Elkington
Moriah	Ellen
Lee	Ellington
Jo	Ellins
Jo	Ellins
Rachel	Elliot
Catherine	Elliott
Emma	Elliott
Jacobi	Elliott
Jacobi	Elliott
Jim	Elliott
Marc N.	Elliott
Meghan	Elliott
Meghan J.	Elliott
Rosalind	Elliott
Katherine	Ellis
Michael J.	Ellis
Naomi	Ellis
Charles	Ellis Jr.
Caroline	Ellis‐Hill
Glena	Ellitt
Adrian	Elmi‐Terander
Carina	Elmqvist
Walaa	Elnaggar
Heather	Elphick
Roni	Elran‐Barak
Els	Els Dozemane
Aladine	Elsamadicy
Helen	Elsey
Jennifer	Elston‐Lafata
Gerald	Elsworth
Rebecca	Elvey
Ann‐Kristin	Elvrum
Glyn	Elwyn
Rosiel	Elwyn
Petri	Embregts
Mark	Embrett
Margaret R.	Emerson
Suna	Emir
Lynne	Emmerton
Violeta	Enea
Janice	Eng
Lucien	Engelen
Ellen G.	Engelhardt
Bryant R.	England
Rachael	England
Kim	Engler
My	Engström
Priscilla	Ennals
Elissa	Epel
Fayron	Epps
Sacha	Epskamp
Ronald	Epstein
Tracy	Epton
Seda	Erdem
Mine	Ergelen
Loretta	Erhunmwunsee
Kristine M.	Erlandson
Joachim	Erlenwein
Joerg	Ermann
Mareike	Ernst
Nicole A.	Errett
Simon	Erridge
Bente	Ervik
Jo	Erwin
Kevin	Escandón
Antoine	Eskander
Ahmad Ali	Eslami
Maryam	Eslami‐Amirabadi
Aneez	Esmail
Katelyn	Esmonde
Albert	Espelt
Susanna	Esposito
Isabel Cristina	Esposito Sorpreso
Carole A.	Estabrooks
Emee	Estacio
María‐José	Estebanez‐Pérez
Shahram	Etemadifar
Jean‐François	Ethier
Bella	Etingen
Egbe B.	Etowa
Josephine	Etowa
Stella	Evangelidou
Bridie	Evans
Catherine	Evans
David	Evans
Gareth	Evans
Rachael	Evans
Rhiannon	Evans
Subhadra	Evans
Sue	Evans
Anna	Evanson
Miriam	Evensen
Amanda	Everall
Lisbeth A.	Evered
Bronwyn	Everett
Regula	Everts
Lizet van	Ewijk
Catherine	Exley
Cornelia	Exner
Deinera	Exner‐Cortens
Naseeb	Ezaydi
Allison M.	Ezzat
Chantal Ski	F.
Bruce	F. Chorpita
Edna	F. ROCHE
Alex	F. Peahl
Andrea	Fabbo
Alice	Fabbri
Margherita	Fabbri
Valeria	Fabre
Jemma	Facenfield
Karen	Facey
Karen	Facey
Roni	Factor
Katleen	Fagard
Antoinette	Fage‐Butler
Guy	Fagherazzi
Mark A.	Faghy
Mathumalar Loganathan	Fahrni
Eileen	Fahy
Inmaculada	Failde
Cynthia	Fair
Hannah	Fair
Hannah	Fairbrother
Ciaran Michael	Fairman
Paolo	Fais
Afolasade	Fakolade
Marie	Falahee
Anna	Falcó‐Pegueroles
Stephanie	Falke
Michelle	Falkenbach
Daisy	Fancourt
Karin	Fängström
Palmira	Faraci
Mansoureh Ashghali	Farahani
Mahbobeh	Faramarzi
Albert J.	Farias
Marcela	Farias
Nicolas	Farina
Michele	Farisco
Carrie M.	Farmer
Jane	Farmer
Sara	Farnbach
Louise	Farnworth
Morag	Farquar
Lorna	Farquharson
Michelle	Farr
Nesrine Saad	Farrag
Paul	Farrand
Albert	Farre
Erina L.	Farrell
Naomi	Farrington
Maree	Farrow
Rafey	Faruqui
Alfonso	Fasano
Daniel B.	Fassnacht
Farhad	Fatehi
Alison	Faulkner
Denise	Faulks
Alice	Faux‐Nightingale
Mina	Fazel
Seena	Fazel
Pariya L.	Fazeli
Sam	Fazio
Adriana	Feder
Stacey	Fedewa
Angela	Fedi
Evelina	Fedorenko
Sophia	Fedorowicz
Thomas Hugh	Feeley
James A.	Feinstein
Inna	Feldman
Mitchell	Feldman
Steven R.	Feldman
Andrea	Feldstain
Eleonora	Feletto
Gracia	Fellmeth
Barbara	Fenesi
Shuo	Feng
Xiaoqi	Feng
Natalie	Fenn
Rachel M.	Fenning
Ama	Fenny
Sarah‐Jane	Fenton
Rebecca	Feo
Gillian	Fergie
Caleb	Ferguson
Caleb	Ferguson
Jane	Ferguson
Ryan E.	Ferguson
Olivier	Ferlatte
Lorna	Fern
Venesser	Fernandes
Alicia	Fernandez
Cara	Fernandez
Jose Louis	Fernandez
Michelle	Fernandez
Erik	Fernandez y Garcia
Sara	Fernández‐Basanta
Martina	Fernandez‐Gutierrez
Cayetano	Fernández‐Sola
Gianluigi	Ferrante
Cristiana	Ferrari
Manuela	Ferrari
Cinzia	Ferraris
Daniel	Ferreira
Joaquim J.	Ferreira
Mireia	Ferri
Elizabeth D.	Ferucci
Garumma Tolu	Feyissa
Klemens	Fheodoroff
Richard	Fielding
Kirsten	Fiest
Daniela	Figueiredo
Johanne	Filiatrault
Gerasimos	Filippatos
Mirko	Filippetti
Graziella	Filippini
Linard	Filli
Serena	Filoni
Emma	Finch
Mary G.	Findling
Sarah	Finer
Monika E.	Finger
Natalie A.	Fini
Regina M.	Fink
Kenneth	Finlayson
Marcia	Finlayson
Marcia	Finlayson
Yvonne	Finn
Andrew	Finney
Caitlin A.	Finney
Sam	Finnikin
Maria	Fiol‐deRoque
Maddalena	Fiordelli
Chiara	Fioretti
Prof Julie	Fish
R.	Fish
Matthew	Fisher
Alana	Fisher
Allison P.	Fisher
Helen	Fisher
Lawrence	Fisher
Marisa H.	Fisher
Pamela	Fisher
Peter	Fisher
Rebecca	Fisher
Tamsin	Fisher
Zoe A.	Fisher
Michelle S.	Fitts
Amanda	Fitzgerald
Christine	FitzGerald
James J.	FitzGerald
Kathryn	Fitzgerald
Niamh	Fitzgerald
Elizabeth M.	Fitzpatrick
Scott J.	Fitzpatrick
Suzanne	Fitzpatrick
Gemmae	Fix
Kaitlyn M.	Fladeboe
Johan	Flamaing
Joanne	Flavel
Fenella	Fleischmann
Jori	Fleisher
Anniken	Fleisje
Jennifer M.	Fleming
Theresa (Terry)	Fleming
Jennifer A.	Flemming
Kate	Flemming
Marcel	Flendrie
Monica	Fletcher
Marie‐Josée	Fleury
Sarah	Flicker
Hazel	Flight
Maria	Flink
Koliouli	Flora
Lidiane Lima	Florencio
Marjo	Flykt
Marjo	Flykt
Darren	Flynn
Andrea	Fogarty
Flavio S.	Fogliatto
Zora	Föhn
Geraldine	Foley
Kimberley A.	Foley
Tony	Foley
Michał	Folwarczny
Ted C. T.	Fong
Erica	Fongaro
Ana	Fonseca
Guillaume	Fontaine
Cheryl	Forchuk
Rachel	Forcino
Alexander C.	Ford
Catherine Elaine Longworth	Ford
Elizabeth	Ford
Joseph	Ford
Tori	Ford
Greta	Forlani
Andreas	Fors
Hans	Forssberg
Alice S.	Forster
Martin	Forster
Katrina	Forsyth
L.	Forsythe
Laura P.	Forsythe
Laura P.	Forsythe
Karen L.	Fortuna
Jennifer Margaret	Fortune
Jennifer	Fortune
Christina	Foss
Ellie	Fossey
Carolyn C.	Foster
Claire	Foster
Cynthia Ewell	Foster
John H.	Foster
Kim	Foster
Mandie	Foster
Michele	Foster
Theresa	Foster
James	Fotheringham
Denis	Fouque
Simona	Fourie
Thomas	Fovet
Cathrine	Fowler
Sally	Fowler Davis
Sally	Fowler‐Davis
Chris	Fox
Deborah	Fox
Joanna	Fox
Louis	Fox
Rachel	Fox
Siobhan	Fox
Una	Foye
Elizabeth	Fradgley
Annika	Frahsa
Emma	France
Emma	France
Emma	France
Janis L.	France
Matteo	Franchi
Emilie	Francis‐Auton
	Franco
Elizabeth	Franklin
Sue	Franklin
Mirjam P.	Fransen
Mirjam P.	Fransen
Arie	Franx
Peter L.	Franzen
Emily	Franzosa
Alec	Fraser
Anne	Fraser
C.	Fraser
Jennifer	Fraser
Kimberly D.	Fraser
Helena C.	Frawley
Jane	Frawley
Timothy	Frawley
Kate	Frazer
Susan K.	Frazier
Rosanne	Freak‐Poli
Janet K.	Freburger
Kristian Steen	Frederiksen
Nina	Fredland
Karen	Fredriksen‐Goldsen
Mio	Fredriksson
Rolien	Free
Angela	Freeman
Jennifer	Freeman
Toby	Freeman
M.	Freestone
Claudia de	Freitas
Claudia de	Freitas
Daniela Fonseca	Freitas
David	French
David	French
Paul	French
Bram	Fret
Alexandra M.	Frețian
Rosemary	Frey
Sean M.	Frey
Julia	Fricke
Oliver	Fricke
Jared S.	Fridley
Eiko	Fried
Esther M.	Friedman
Orsolya	Friedrich
Kathleen	Friel
Sharon	Friel
Roland	Friele
Noud	Frielink
Daniel	Frings
Nora E.	Fritz
Dennis	Frohlic
Andrea	Frolic
Andrea	Frosolini
Rachael	Frost
Gasto	Frumence
Michal	Frumer
Margaret	Fry
Margaret	Fry
Caroline	Fryer
Nancy	Fu
Yu	Fu
Ahmad	Fuady
Sandra	Fucile
Nina	Fudge
Daniel	Fuerstenau
Stefania	Fugazzaro
Daniel	Fulford
Jeffrey	Fuller
Naomi	Fulop
Carles Martin	Fumadó
Simona	Fumagalli
Kin Wah	Fung
Laura	Funk
Martha	Funnell
Christine	Furber
Jennifer J.	Furin
John	Furler
Per	Fürst
Olga	Fuseini
Adam	Fusheini
Beth	Fylan
Richard	G. Kyle
Constantine	G. Lyketsos
Brandon	G. Bergman
Joan	G. Carpenter
Maha El	Gaafary
Mark	Gabbay
Robert A.	Gabbay
Mirko	Gabelica
Hesham Fathy	Gadelrab
Piyada	Gaewkhiew
Harry	Gaffney
Anna	Gagliardi
Anna	Gagliardi
Marie‐Pierre	Gagnon
P.	Galanis
Alison A.	Galbraith
Nicola	Gale
Thomas	Gale
Jerome T.	Galea
Martina	Galeotti
Jacqueline	Galica
Elizabeth	Galik
Ann	Gallagher
Colleen	Gallagher
Mary P.	Gallant
Jorge	Gallardo
Frode	Gallefoss
Danielle	Gallegos
Carol L.	Galletly
Jessica	Galli
Michele	Gallo
Maria Helena	Galvão
Jaime Paneque‐	Gálvez
Rose	Galvin
Kristi E.	Gamarel
Giuditta	Gambino
Julie	Gamble‐Turner
Julia	Game
Chloe	Gamlin
Eduardo	Gandara
Marialuisa	Gandolfi
Zanetta	Gant
Joanne	Ganton
Freda DeKeyser	Ganz
Caroline	Gao
Mi Mi	Gao
Xiu	Gao
Pamela M.	Garabedian
Montse	Garcia
Sofia F.	Garcia
Diego Santos	García
Susana	García Gutiérrez
Miguel	Garcia‐Argibay
Maitane	Garcia‐Lopez
Carmen	García‐Peña
Francisco	García‐Torres
Luz	Garcini
Clare	Gardiner
Caroline	Gardner
Kathryn	Gardner
Sara	Garfield
Kranti	Garg
Pankaj	Garg
Ian W.	Garner
Mark	Garner
Anna	Garnett
Veerle	Garrels
Marco	Garrido‐Cumbrera
Lorenza	Garrino
Scott R.	Garrison
Deirdre	Gartland
Mirjam	Garvelink
Mirjam M.	Garvelink
K.	Gaskin
Chiara	Gastaldon
Dimitrios	Gatsios
Jane R. von	Gaudecker
Joseph	Gaugler
Iris	Gault
Surabhi	Gautam
Lynne Vonda	Gauthier
Amélie	Gauthier‐Beaupré
Maria Beatriz Duarte	Gavião
James	Gavin
Caryl	Gay
Valerie	Gay
Charlotte A.	Gaydos
Shena	Gazaway
Caleb	Gbiri
Peicong	Ge
Montserrat	Gea‐Sánchez
Azeb	Gebresilassie Tesema
Yonas E.	Geda
Andrew	Geddis‐Regan
Andrew	Geddis‐Regan
Stephan	Gehring
Emma	Geijer‐Simpson
Franziska	Geiser
Emel	Genc
Melissa	Gench
Tingting	Geng
Jochen	Gensichen
Erin K.	George
Johnson	George
Molly	George
Steven Z.	George
Badicu	Georgian
Dejan	Georgiev
Apostolos	Georgopoulos
Ehab	Georgy
Aisling A.	Geraghty
Fatemeh	Geranmayeh
Karen	Gerard
L. A. A.	Gerbens
Andrea	Gerlak
Gino	Gerosa
Robin	Gerrits
Debby	Gerritsen
A. E.	Gerritsen
Esther	Gerritzen
Elisa	Gervasoni
Sabina	Gesell
Gert J.	Geurtsen
Rosa	Geurtzen
Rémon	Geuze
Ateret	Gewirtz‐Meydan
Monica A.	Ghabrial
Ali	Ghaddar
Setareh	Ghahari
Atousa	Ghahramani
Solmaz	Ghanbari‐Homaie
Masoud	Gharib
Saruna	Ghimire
Daniela	Ghio
Sergio	Ghirardo
Dr. Majorie	Ghisoni
Shima	Gholamalishahi
Mahdia	Gholami
Malabika	Ghosh
Manonita	Ghosh
Muhammad Thesa	Ghozali
Rebecca	Giallo
Pucciarelli	Gianluca
Vaitsa	Giannouli
Daniele	Giansanti
Chris	Gibbons
Chris	Gibbons
Elizabeth	Gibbons
Lisa	Gibbs
Susannah	Gibbs
Andy	Gibson
Andy	Gibson
Barbara	Gibson
Faith	Gibson
Rosemary	Gibson
Godwin Denk	Giebel
Jennifer M.	Gierisch
Anik	Giguere
Keon L.	Gilbert
Frederic	Gilbert
Ileen	Gilbert
Laura Sisola	Gilchrist
Philippe T.	Gilchrist
Emma L.	Giles
Michelle	Giles
Sally	Giles
Meghan	Gilfoyle
Diana	Gil‐González
Andrea	Gilkison
Emily	Gill
Fenella	Gill
Justyna Bandola‐	Gill
Kate	Gill
Paul	Gill
Steve	Gillard
Steven	Gillard
Brigid	Gillespie
Gordon Lee	Gillespie
Judy	Gillespie
Robyn	Gillespie
Stephanie	Gillibrand
Stephanie	Gillibrand
Kate	Gillies
Megan	Gilligan
Catharina	Gillsjö
Tameka	Gillum
Janneke De Man‐van	Ginkel
Liane	Ginsburg
Michael	Gionfriddo
Vito	Giordano
Gabriele	Giorgi
Guido	Giovanardi
Elodie	Girard
Nicolas	Giraudeau
Jane	Girling
Tre	Gissandaner
Angela N.	Gist‐Mackey
Alberto	Giubilini
Laura	Giuntoli
Emanuele M.	Giusti
Ali	Giusto
Muluken	Gizaw
Evgenia	Gkintoni
Anna	Gkiouleka
Jon	Glasby
Karen	Glaser
Kathryn M.	Glaser
Manuela	Glattacker
Catherine R.	Glenn
Mia	Glerup
Jennifer L.	Glick
Rainer	Gloeckl
Fabienne	Glowacz
Robe	Glynne‐Jones
Kristin N.	Gmunder
Vivian	Go
Nina	Gobat
Robbert J. J.	Gobbens
Joslyn	Gober
Kali	Godbee
M.	Godfrey
Catarina	Godinho
William	Godolphin
William C.	Goedel
Dianne P.	Goeman
Katja	Goetz
Sarah L.	Goff
Anita	Goh
Patrick K.	Goh
Figen	Gökçay
Ozden	Gokdemir
Rebecca	Gokiert
Irving	Gold
Jeffrey I.	Gold
Marthe R.	Gold
Sara	Golden
Danielle	Goldfarb
Sharon	Goldfeld
Lucy Pollyanna	Goldsmith
Ellen	Goldstein
Roger	Goldstein
Jason	Goldstick
Sandra	Goldsworthy
Joanna	Goldthorpe
Grace	Gombolay
Fabio	Gomes
Sagrario	Gomez
Javier	Gomez‐Pilar
Prakash	Gondode
Silvia	Gonella
Gilbert	Gonzales
Miriam	Gonzalez
Nerea	Gonzalez
Jesus	Gonzalez‐Lama
Pedro	Gonzalo
Rachael	Gooberman‐Hill
Karen	Goodall
Debbie	Goode
James R.	Gooden
Claire	Goodman
Jessica Marian	Goodman‐Casanova
Jessica Marian	Goodman‐Casanova
Joanna	Goodrich
Donna	Goodridge
James	Goodwin
John	Goodwin
Nick	Goodwin
Susan	Goold
Anne	Goossensen
Dipesh	Gopal
Mira	Goral
S. J.	Gordijn
Adam Lee	Gordon
Howard	Gordon
Howard	Gordon
Lisi	Gordon
Sarah	Gordon
Susan	Gordon
Nick	Gore
Kim A.	Gorgens
Ashraf S.	Gorgey
Marloes van	Gorp
Sylwia	Gorska
Jan Willem	Gorter
Jan Willem	Gorter
Hayley	Gorton
Sara	Gostoli
Poonam	Goswami
Helga	Gottfredsdottir
Nora	Gottlieb
Karla	Gough
Beatriz	Goulão
Rebecca L.	Gould
Rebecca	Goulding
Ram	Gouripeddi
Dianne	Gove
Ishtar	Govia
Genevieve	Graaf
Ted G.	Graber
Klemenn	Grabljevec
Daniel	Grace
Rebekah	Grace
Fergus	Gracey
William D.	Graf
Lesley A.	Graff
Blair	Graham
Christina	Graham
Christopher	Graham
Cynthia	Graham
Ian	Graham
Tanya	Graham
Yitka	Graham
Rebecca	Grainger
Mateusz	Grajek
Robert	Gramling
Stuart	Grande
Sonia M.	Grandi
Ulla Halgren	Graneheim
Mats	Granlund
Aileen	Grant
Aimee	Grant
Andrew	Grant
Julia	Grass
Scott	Graupensperger
Lucinda J.	Graven
Aaliyah	Gray
Ben	Gray
Kathleen	Gray
Patricia	Gray
Tamryn F.	Gray
Richard	Gray
Kara A.	Gray‐Burrows
Federica	Graziano
Annmarie	Grealish
Colin	Greaves
Gizell	Green
Jenn	Green
Jennifer Greif	Green
Jon	Green
Jonathan	Green
Julie	Green
Suetonia	Green
Judith	Green Mckenzie
Stephen J.	Greene
Alexandra	Greene
Kathryn	Greene
Saara	Greene
Talya	Greene
Ian	Greener
Beatrice	Greenfield
David	Greenfield
Geva	Greenfield
Thomas K.	Greenfield
Trish	Greenhalgh
Kate	Greenwell
Hannah	Greenwood
Kathryn	Greenwood
Sharlene	Greenwood
Ben	Greer
Celia	Gregson
Pippa	Grenfell
Laura Elisabeth	Gressler
Maddy	Greville‐Harris
Ruby	Greywoode
Suzanne M.	Grieb
Jessica	Grieger
Sharon	Grieve
Joan	Griffin
Percy	Griffin
Sian	Griffin
Simon	Griffin
Tomás P.	Griffin
Alys	Griffiths
Chris	Griffiths
Kathy	Griffiths
Peter	Griffiths
Christiane	Grill
Stephanie A.	Grilo
Lars J.	Grimm
Chloe	Grimmett
Cheryl	Grindell
Konstadina	Griva
Michael P. W.	Grocott
Patricia	Grocott
Oliver	Groene
Vigdis Abrahamsen	Grøndahl
Mette	Grønkjær
Toto	Gronlund
Wim	Groot
Daniel F.	Gros
Douglas	Gross
Linda	Grosser
Alessandra Agnese	Grossi
Amy	Grove
Christine	Grove
Prateek	Grover
Serena	Grumi
Anna H.	Grummon
Hanna	Grundström
A. C.	Grundy
Andrew C.	Grundy
Luke E.	Grzeskowiak
Ting	Guan
Leandro S.	Guedes
Cornelia	Guell
Andrea	Guerra
Ivan	Guerreiro
Giuliana	Guggino
Neeraj	Gugnani
Chandana	Guha
I. Neil	Guha
Sara	Guilcher
Jessica	Guinane
Elaine de	Guise
Veslemøy	Guise
Rani	Gul
Martin	Gulliford
Hannah L.	Gullo
Monika Knudsen	Gullslett
Oya	Gumuskya
Hendra	Gunawan
Mintaze Kerem	Gunel
Christine	Gunn
Christine M.	Gunn
Jane	Gunn
Kate	Gunn
Karen	Gunnell
Lena	Gunningberg
Ling	Guo
Mughi	Guo
Qi	Guo
Aditi	Gupta
Dipankar	Gupta
Harish	Gupta
Samir	Gupta
Veenu	Gupta
Veenu	Gupta
Veenu	Gupta
Dawne	Gurbutt
Lindsey	Gurin
Sepali	Guruge
D.	Gurung
Gagan	Gurung
Rejina	Gurung
Maria	Gustafsson
Catharina	Gustavsson
Corina	Güthlin
Regina	Guthold
Steven	Guthridge
Dawn M.	Guthrie
Lorena	Gutiérrez‐Hermoso
Kerry	Gutridge
Agnieszka	Guzik
Jaime	Guzman
Hanna	Gyllensten
Zsuzsa	Győrffy
Simone	H. Crouch
Ashley	H. Ng
Juanita	Haagsma
Juliet	Haarbauer‐Krupa
Maria	Haarmans
Romi	Haas
Jolanda C. M. van	Haastregt
Travis	Haber
Jessica E.	Haberer
Christina	Hackett
Maree L.	Hackett
Rebecca	Haddock
	Hadjiat
Michelle	Hadjiconstantinou
Heather	Hadjistavropoulos
Thomas	Hadjistavropoulos
Emina	Hadziabdic
Kinga	Hadzsiev
Julie	Haesebaert
Kåre	Hagen
Virginia	Hagger
Arja	Häggman‐Laitila
Magnus	Hagiwara
Pola	Hahlweg
Hyeouk Chris	Hahm
Joan	Hahn
Sharif	Haider
Motti	Haimi
Kimberley	Haines
Kimberley J.	Haines
Neil	Hair
Bridget	Haire
André	Hajek
Faisal	Hakeem
Hadeel	Halaweh
Elizabeth	Halcomb
Elizabeth	Halcomb
Anne‐Grethe	Halding
Sarah	Hales
William E.	Haley
Georgia	Halkett
Katherine	Hall
Abigail J.	Hall
Alex	Hall
Alix	Hall
Helen	Hall
Trevor A.	Hall
Sally	Hall Dykgraaf
Julie	Hallet
Emily	Hallgren
Marie Louise	Hall‐Lord
Kate	Hallsworth
Bonnie	Halpern‐Felsher
Tanya	Halsall
Christine	Halse
Joschka	Haltaufderheide
Marianne Berg	Halvorsen
Liv	Halvorsrud
Miki	Hamada
Marije	Hamaker
Yaira	Hamama‐Raz
Johannes	Hamann
Johannes	Hamann
Sarah	Hamed
Samer	Hamidi
Mohammad	Hamiduzzaman
Mohammad	Hamiduzzaman
Alison	Hamilton
Bridget	Hamilton
Clayon	Hamilton
Jessica L.	Hamilton
Marysia	Hamilton‐Kirkwood
Alison	Hammond
Jonathan	Hammond
Sahar	Hammoud
Omar	Hammouda
Bente	Hamnes
Ilyes	Hamouda
Hae‐Ra	Han
Paul	Han
Volkan	Hanci
Nicola	Hancock
Annette	Hand
Brittany N.	Hand
Charlotte	Handberg
Reed	Handlery
Melanie	Handley
Ingrid	Handlovsky
Deepa	Handu
Peter	Hangoma
Matthew	Hankins
Bec	Hanley
Stephanie	Hanley
Bret M.	Hanlon
Charlotte	Hanlon
Mark	Hann
Kerry	Hanna
Ben	Hannigan
Kathleen	Hannon
Susan	Hannon
Susan M.	Hannum
Barbara	Hanratty
Henrik	Hansen
Dharinee	Hansjee
Sarah	Hanson
Brenda	Happell
Natasha	Hardicre
Jessica	Hardin
Emma	Harding
Katherine E.	Harding
Janet	Hardy
Stephen	Harfield
Ian	Hargraves
Deena P.	Harji
Guy	Harling
Marline	Harmsen
Joanna	Harnett
Nigil	Haroon
ALice	Harper
Candace	Harrington
Daniel W.	Harrington
Dean A.	Harris
Holly	Harris
Janet	Harris
Melissa	Harris
R.	Harris
Cheryce L.	Harrison
Elizabeth	Harrison
James	Harrison
Lauren	Harrison
Mark	Harrison
Reema	Harrison
Rosie	Harrison
Ben	Harris‐Roxas
Erin Nicole	Harrop
Jo	Hart
Laura	Hart
Laura C.	Hart
Martin	Härter
Martin	Härter
Samantha	Hartley
Lisa	Hartling
Nicholas D.	Hartman
Tim	Hartman
Gillian	Harvey
Idethia Shevon	Harvey
Sam	Harvey
Matire	Harwood
Cristina	Has
Rim	Hasan
Shahzad	Hasan
Erin Rutherford	Hascup
Wolfgang	Hasemann
Kelly	Haskard
Divna M.	Haslam
Shaima	Hassan
Angela	Hassiotis
Carolyn Ruth	Hastie
Paul D.	Hastings
Andrew	Hatala
Adel	Hatami‐Marbini
Taku	Hatano
Elham	Hatef
Randula	Haththotuwa
Irene E.	Hatsu
Chris	Hatton
Mona‐Iren	Hauge
michael	Haugh
Katrina	Hauschildt
Andrea	Hausheer
Elliott R.	Haut
Farinaz	Havaei
Lotte	Haverman
Lotte	Haverman
Ashley	Hawarden
Jacinta	Hawgood
Rhiannon	Hawkes
Alex	Hawkey
Jemma L.	Hawkins
Phillip	Hay
Keiko	Hayano
Melissa	Hayden
Helen M.	Haydon
Corey	Hayes
Daniel	Hayes
Sara	Hayes
Emily	Haynes
Glen	Hazlewood
Afeez Abiola	Hazzan
Ping	He
Shan	He
Emma	Healey
Sean	Healy
Patricia	Healy
Sarah	Healy
Jasmine	Hearn
Vanessa	Heaslip
Gemma	Heath
Anne	Heaven
Molly	Hebditch
Birgit	Heckemann
Lindsay	Hedden
Katarina	Hedin
Anna Karin	Hedström
Christoph	Heesen
Olivia	Heffernan
Anna	Hegedues
Liv‐Helen	Heggland
Ellie	Heidari
Melissa	Heightman
Katharina	Heimerl
Benedetta	Heimler
Konstantin G.	Heimrich
Sue P.	Heiney
Vanessa N.	Heitplatz
Rikke	Helene Moe
Bruno	Heleno
Magnus	Helgesson
Ragnhild	Hellesø
Sarah	Hellewell
Toby	Helliwell
Andreas	Hellström
Karin	Hellström
Marc H.	Hemmelder
N. F.	Hempler
Nana Folmann	Hempler
Coralie De	Hemptinne
Emily	Henderson
Emily	Henderson
Jo	Henderson
Joanna	Henderson
R. Claire	Henderson
Wendy A.	Henderson
Andrew	Hendifar
Tereza	Hendl
Caroline	Hendricks
Gordon J.	Hendry
Karen M. Oude	Hengel
Aldiene	Hengelaar
Eilis	Hennessy
Silje Havrevold	Henni
Carrie	Henning‐Smith
Jens	Henrichs
Pontus	Henriksson
Roland	Henry
Anna	Henschel
I.	Henselmans
Inge	Henselmans
Catherine	Henshall
Tarja	Heponiemi
Lauren	Hepworth
Dilinie	Herbert
Franziska A.	Herbst
Fernando	Herkrath
Lize	Hermans
Rosella	Hermens
Eulàlia	Hernández Encuentra
Helen	Herrman
Jolyn	Hersch
Deborah	Hersh
Deborah	Hersh
Nicola	Heslehurst
Sarah	Hetrick
James Christoffel	Heunis
Christian	Heuser
Christian	Heuser
Sabina	Heuss
Marloes	Heutmekers
Michelle	Heward
Jenny	Hewison
Claudia	Hey
Katja	Heyduck
Sophie E.	Heywood
Anne	Hickey
Gary	Hickey
Martha	Hickey
Ian	Hickie
Caitlin W.	Hicks
Vanesa	Hidalgo
Samantha	Hider
Adele	Higginbottom
Agnes	Higgins
Mary	Higgins
Peter	Higgins
Robert	Higgins
I. J.	Higginson
Katerina	Hilari
Zoe	Hildon
Courtney	Hiley
Briony	Hill
Catherine	Hill
Jonathan	Hill
Sophie	Hill
Marij	Hillen
Susan	Hillier
Anne	Hillman
Danny	Hills
Medbh	Hillyard
Grace C.	Hillyer
Andrea	Hilton
Cesar A.	Hincapié
Jesse	Hinckley
Daniel	Hind
Anette Lykke	Hindhede
Rochelle	Hine
Sonia	Hines
Kathryn	Hinsliff‐Smith
Lisa	Hinton
Verena	Hinze
Tetsuya	Hirata
Shivayogi V.	Hiremath
Marina	Hirjaba
Oliver	Hirsch
Julia	Hiscox
Danielle	Hitch
Carol	Hitchon
Elisabet	Hjorleifsdottir
Emily S.	Ho
Roger C.	Ho
Ha	Hoang
Mollie	Hobensack
Sebastian	Hobson
Lenka	Hodacova
Pat	Hoddinott
Matthew	Hodes
Victoria	Hodges
Femke	Hoekstra
Pieter J.	Hoekstra
Jerry K.	Hoepner
Michael	Hoerger
Marieke P.	Hoevenaar‐Blom
Jeanne M.	Hoffman
Laura	Hoffmann
Tammy	Hoffmann
Magdalena	Hoffmann
Caroline	Hoffstedt
Luiza	Hoga
Anne	Hogden
Olivia	Hogue
Aaron	Hogue
Marikken	Høiseth
Mel	Holden
Stephanie	Holliday
Olivia	Hollingdrake
Samantha	Hollingworth
Brendon T.	Holloway
Saron	Holly
Amelia	Hollywood
Lotte	Holm
Jack	Holman
Daniel	Holman
Mats	Holmbert
Michelle M.	Holmes
Margaret	Holmes‐Rovner
Elizabeth	Holmes‐Truscott
Arja	Holopainen
Eleanor	Holroyd
Ditte H.	Holt
Natalie R.	Holt
Nicole	Holt
Sara	Holton
Sara	Holton
Melanie	Hom
Catherine Verity	Homer
Mieko	Homma
Sailish	Honap
Anne	Honey
P.	Hong
Robert S.	Hong
Y. Alicia	Hong
Zhi‐Jie	Hong
Michael C.	Honigberg
Simone	Honikman
Suzanne	Hood
Barbara J. van den	Hoofdakker
Emiel O.	Hoogendijk
Alison	Hooper
Elske	Hoornenborg
Thomas A.	Hope
Suellen	Hopfer
Brandon	Hopkins
Jane	Hopkins
Liza	Hopkins
Isobel	Hoppe
Louise	Hopper
Ann	Hopton
Suyin	Hor
Gonul Dinc	Horasan
Ulrica	Horberg
Frances	Horgan
S. Ben	Horin
Shigeko	Horiuchi
Marcia	Horn
Maria	Horne
Maria	Horne
Åsa	Hörnsten
Rianne	Hornstra
Antje	Horsch
Haley Kranstuber	Horstman
Daniel	Horton
Jeremy	Horwood
Md Sazzad	Hosen
Ahmed	Hossain
M. Mahbub	Hossain
Muhammad	Hossain
Hassan	Hosseinzadeh
Cody	Hostutler
Emily J.	Hotton
Xiaorong	Hou
Linzy	Houchen‐Wolloff
Catherine	Houghton
John	Houghton
Kim	Houlind
L. Duane	House
Amy J.	Houtrow
Juul	Houwen
Natasha	Howard
Louise M.	Howard
Matt C.	Howard
Ileana M.	Howard
Stephanie	Howard
Jo	Howe
Samantha	Howe
Andrew J.	Howell
Doris	Howell
Doris	Howell
Martin	Howell
Mette Terp	Hoybye
Pei‐Shan	Hsieh
Suh‐Ing	Hsieh
Chao‐Kai	Hsu
Lewis L.	Hsu
Sheng‐Der	Hsu
Wei‐Chung	Hsu
Mila Nu Nu	Htay
Wendy	Hu
Wen‐Yu	Hu
Fei Fei	Huang
Jennifer	Huang
Terry	Huang
Ya‐Ling	Huang
Yueqin	Huang
Yunyun	Huang
Zhengfei	Huang
Zhengzong	Huang
Yumin	Huang‐Link
Jutta	Hübner
M.	Huchko
Catherine	Hudon
Briony F.	Hudson
Briony F.	Hudson
Chloe C.	Hudson
Jennifer	Hudson
Amy G.	Huebschmann
Tanja	Huesch
Christy	Huff
Stephen	Hughe
Carmel	Hughes
David	Hughes
Jane	Hughes
Lyndsay	Hughes
M. Courtney	Hughes
Mary	Hughes
Stephanie	Hughes
Shana	Hughes
Siobhan	Hugh‐Jones
Jo‐anne	Hughson
Z.	Hui
Jennifer M.	Hulett
Erik Hj	Hulzebos
Sofia von	Humboldt
Sandra	Hummel
John M.	Humphrey
Tracy	Humphrey
Gillian	Hundt
Florence Van	Hunsel
Andrew P.	Hunt
Glenn E.	Hunt
Kate	Hunt
Kelly	Hunt
Benton R.	Hunter
Michael	Hunter
Rachael	Hunter
Sue	Hunter
Sabina	Hunziker
Maisha R.	Huq
Katja van den	Hurk
Deirdre A.	Hurley
R. Andrew	Hurley
Daniela	Husárová
Andy	Husband
Bettina S.	Husebo
Anne Marie Lunde	Husebø
Kerryn	Husk
Saira	Hussain
Norita	Hussein
Norita	Hussein
Ziad	Hussein
Sally	Huston
Jane	Hutchens
Alison	Hutchinson
Anastasia	Hutchinson
Ann	Hutchinson
Ann	Hutchinson
Brian	Hutchison
Karina	Huus
Jongnam	Hwang
Amelia	Hyatt
Catherine	Hyde
Lars‐Christer	Hydén
Nerida	Hyett
Fiona	Hyland
Jinshil	Hyun
Kathryn	Hyzak
Karen	I. Connor
Margaret	I. Fitch
Patricia	I. Moreno
Andrea	Iaboni
Pietro	Iaffaldano
Maya	Ibenfeldt
Bukola Mary	Ibitoye
Norhayati	Ibrahim
Kinda	Ibrahim
Ayumi	Igarashi
Fehintola A.	Ige
Suzanne	Ii
Roya	Ijadi‐Maghsoodi
Maarten	IJzerman
Theresa	Ikegwuonu
Deborah	Ikhile
Chinyere	Ikwuakor
Gabriela	Ilie
Kimberly S.	Illingworth
Nicole	Ilonzo
Fatma	İltuş
Gabriele	Imbalzano
Christine	Imms
Ibrahim Halil	Inanc
Astrid	Indekeu
Elin	Inge
Brooke	Ingersoll
Guglielmo	Inglese
Jenny	Ingram
Bianca Maria Serena	Inguscio
Laura	Inhestern
Anthea	Innes
Kelli	Innes
Domenico	Intiso
Magdalini	Ioannou
Rosa	Iodice
Frank	Iorfino
Halima	Iqbal
Jane	Ireson
Patricia	Irizar
Karoline	Irschara
Andy	Irving
Andi Masyitha	Irwan
Mary J.	Isaacson
Kahabi Ganka	Isangula
Hiroyuki	Isayama
Gözde Gökçe	İsbir
Esma R.	Isenovic
Kodai	Ishihara
Masanobu	Ishii
Hirono	Ishikawa
Miho	Ishimaru
Elif	Isik
Georgi	Iskrov
Nazrul	Islam
Rabiul	Islam
Rakibul	Islam
Shayla	Islam
Noor	Ismael
Hanif	Ismail
Leila	Ismail
Rakhi	Issrani
Maria V.	Ivanova
Mariya	Ivanovska
N. M.	Ivers
Srividya	Iyer
Jonathan	Izudi
David Castle	J.
James	J. Callery
Victoria	J. McGowan
Lee	J. Y.
Jacquelyn	J. Benson
Emily	J. Braun
Natalie	J. Cox
Christopher	J. D. Wallis
Sarah	J. Egan
Lila	J. Finney Rutten
Cameron	J. Gettel
Regina	J. Giraldo‐García
Carol	J. Howe
Barbara	J. Kuter
Leodoro	J. Labrague
Kimberly	J. Petersen
Clayton	J. Shuman
Ashlee	J. Vance
Tiny	Jaarsma
Brigit	Jaarsveld
Susan	Jack
Simon	Jacklin
Adam	Jacks
Chandra L.	Jackson
Clare	Jackson
Mathieu	Jackson
Sandra L.	Jackson
Thomas A.	Jackson
Yves	Jackson
Molly	Jacobs
Helene	Jacobson
Lisa A.	Jacobson
Cristina	Jácome
Constanza	Jacques Aviñó
Farrah	Jacquez
Fabienne	Jaeger
Thomas	Jaenisch
Usman	Jaffer
Annemarie D.	Jagielo
Paweł	Jagielski
Parbir	Jagpal
Heiko J.	Jahn
Tina	Jahnel
Reidun Birgitta	Jahnsen
Mukul	Jain
Jessica	Jaiswal
Benjamin C.	James
Inger	James
Karen	James
Shirley	James
Tyler	James
Hamish	Jamieson
Shazia	Jamshed
Shazia	Jamshed
Stephen	Jan
Gillian	Janes
Insil	Jang
Bhautesh	Jani
Maximiliane	Jansky
Bianca	Jansky
Anna	Janssen
Ellen M.	Janssen
Astrid	Janssens
Astrid	Janssens
Rosanne	Janssens
Bernadetta	Janusz
Ying‐Ling	Jao
Narumol	Jarernsiripornkul
Don Des	Jarlais
Alenxandra	Jarling
Przemysława	Jarosz‐Chobot
Elizabeth Ann	Jarpe‐Ratner
Kajsa	Järvholm
Kathryn	Jarvis
Marjan	Javanbakht
Aysha	Jawed
Krishnamurthy	Jayanna
Ruwan Duminda	Jayasinghe
Mark	Jayes
Fatemeh Rangraz	Jeddi
Stephen	Jeffares
Michael	Jefford
Mark	Jeffries
Marie	Jelinkova
Brenda	Jeng
Zoe	Jenkins
Lee	Jennings
Annesofie Lunde	Jensen
Claus Sixtus	Jensen
Natasja Koitzsch	Jensen
David	Jernigan
Teresa A.	Jerofke‐Owen
Carlos Hernandez	Jerónimo
Jed	Jerwood
Tine	Jess
Sweety Suman	Jha
Jiafu	ji
Fu‐Jun	Jia
Changchuan	Jiang
Boshen	Jiao
Mingli	Jiao
Kon‐Siong	Jie
Jane	Jih
Alberto	Jiménez‐Maldonado
Jing	Jin
Xia	Jing
Karishma	Jivraj
Moyez	Jiwa
Julia	Jockusch
Martin	Joehr
Aud	Johannessen
Monika	Johansen
Jan	Johansson
Linda	Johansson
Nina	Johansson
Amber	John
Ann	John
Oommen	John
John	John Oliffe
Denny	John
Michelle M.	Johns
Helle	Johnsen
Lisa	Johnson
Michael L.	Johnson
Adrienne L.	Johnson
Ensa	Johnson
Eugenie	Johnson
Judith	Johnson
Leslie W.	Johnson
Mark R.	Johnson
Nathalie A.	Johnson
Nia	Johnson
Nicholas	Johnson
Rebecca J.	Johnson
Romaine F.	Johnson
Sara B.	Johnson
Sherryl	Johnson
Sharon	Johnston
Sara	Joice
Anjni Patel	Joiner
Rafael	Jomar
Charles	Jonassaint
Cindy	Jones
Heidi E.	Jones
Christina J.	Jones
Daniel A.	Jones
Elisa	Jones
Fiona	Jones
Fiona	Jones
Georgina	Jones
Geraint	Jones
Hannah	Jones
Jacob Daniel	Jones
Julia	Jones
Karyn O.	Jones
Katy	Jones
Lander	Jones
Laura	Jones
Lauren	Jones
Lyell	Jones
Lynnette	Jones
Marion Moser	Jones
Matthew D.	Jones
Nev	Jones
Randy	Jones
Sophie	Jones
Hannah	Jongebloed
Michelle I.	Jongenelis
Karin R.	Jongsma
Szymon	Jonik
Anneleine	Jonker
Helga	Jonsdottir
Alexandra Brandt Brandt	Jønsson
Pieter	Joosse
Yvonne	Joosten
Gerald	Jordan
Isabel	Jordan
Jo	Jordan
Kelvin	Jordan
Mikaela	Jorgensen
Kim	Jørgensen
Kim	Jørgensen
Kjetil Nordbø	Jørgensen
Lone	Jørgensen
Kim	Jose
Amanda	Joseph
Olivia	Joseph
Natalie	Joseph‐Williams
Fabrice	Jotterand
Tanisha	Jowsey
Angela	Ju
Norha Vera San	Juan
Shannon B.	Juengst
Sofia	Juhlin
Pétur Benedikt	Júlíusson
Sandra	Jumbe
Mao	Jun
Hailey H.	Jung
Neti	Juniarti
Gregor	Jurak
Truls	Juritzen
Jenny	Jw Liu
Satu	Jyväkorpi
Sian Smith	K.
Christabel	K. Cheung
Shannon	K. Bennetts
Maya	K. Gislason
Jean	K. Gordon
Tamar	Kabakian
Ashley	Kable
Michal	Kafri
Eva	Kahana
Outi	Kahkonen
Outi	Kähkönen
K.	Kahn
Robin	Kahn
Turhan	Kahraman
Anu‐Marja	Kaihlanen
Therese	Kairuz
Kaitlyn	kaitlyn Kuryk
Mette	Kalager
Deepak M.	Kalaskar
Shona	Kalkman
Csilla	Kalocsai
Alon	Kalron
Biren B.	Kamdar
Kyosuke	Kamijo
Leonard	Kaminsky
Jochen	Kammermeier
Kendra J.	Kamp
Rejina	Kamrul
Panos	Kanavos
Arti	Kandalam
Tjaša	Kanduč
Joseph P. M.	Kane
William J.	Kane
Takahisa	Kanekiyo
Noriyo	Kaneko
Associate Professor Melissa	Kang
Youjeong	Kang
Mari	Kangasniemi
Mari	Kangasniemi
Vadivelan	Kanniappan
Sabuj	Kanti Mistry
Ira	Kantrowitz‐Gordon
Dhami	Kapadia
Sabi	Kaphle
Anuj	Kapilashrami
Gilaad G.	Kaplan
Z. L. Rana	Kaplan
Sujita Kumar	Kar
Burak	Karaaslan
Marlène	Karam
Nektarios	Karanikas
Fatma	Karapinar
Mumtaz	Karatas
Philippe	Karazivan
Hans	Karbe
Nicole	Karcher
Michele	Karel
Neda	Karimi
Rose‐Marie	Karlsson
Amol	Karmarkar
Justin Elliott	Karr
Anne	Kasper
Jürgen	Kasper
Dimitrios	Kasselimis
Susan L.	Kasser
Marise J.	Kasteleyn
Nick	Kates
Shanna K.	Kattari
Hans D.	Katzberg
Kenneth R.	Kaufman
Manmeet	Kaur
Surinder	Kaur
Jean‐François	Kaux
Anne	Kavanagh
David	Kavanagh
Grace	Kavanaugh
Pawel	Kawalec
Keisuke	Kawata
Lisa	Kawatsu
Emma Sophia	Kay
Melissa C.	Kay
Dan K.	Kaye
François Kaze	Kaze
John	Keady
Stephen	Kearing
Aine	Kearns
Hazel	Keedle
Laurie	Keefer
Julia	Keenan
Richard	Keers
Anju Devianee	Keetharuth
Michelle	Kehoe
Oksana	Kehoe
Sarah A.	Keim
Lauren	Kelada
Lauren	Kelada
Pallavi	Kelkar
Sinéad	Kelleher
Anastasia	Keller
Jim	Kellner
Anne‐Maree	Kelly
Berni	Kelly
C. M.	Kelly
Jaimon	Kelly
Janet	Kelly
Karen	Kelly‐Blake
Rebecca	Kelly‐Campbell
Kathleen	Kendall
Tony	Kendrick
Tony	Kendrick
Tetyana	Kendzerska
Alison	Kennedy
Anne	Kennedy
Anne	Kennedy
Catriona	Kennedy
Cole J.	Kennedy
Maura	Kennedy
Maura	Kennedy
Norelee	Kennedy
Katherine	Kenny
Patricia	Kenny
Louise	Keogh
Sarah C.	Keogh
Syed Afroz	Keramat
Angeliki	Kerasidou
Kyle A.	Kercher
Nóra	Kerekes
Courtney	Kerestes
Connor	Kerns
Claire	Kerr
Ian H.	Kerridge
Julia	Kerschbaum
Paula	Kersten
Diana	Kerwin
Prashant	Kesharwani
Lars Vedel	Kessing
David	Kessler
Rita	Kessler
Jessica M.	Ketchum
Victoria E.	Kettle
O'Leary	Kevin
Ehsan	Keykhosravi
Chris	Keyworth
Amor	Khachemoune
Kate	Khair
Saman	Khalatbari‐Soltani
Hanan	Khalil
Asma	Khalil
Hossein	Khalili
Mandana	Khalili
Fary	Khan
Jahangir	Khan
Menal	Khan
Nagham	Khanafer
Ashrafunnesa	Khanom
Kalpa	Kharicha
Nadia	Khartabil
Resham B.	Khatri
Utsha G.	Khatri
Maryam	Khazaee‐Pool
Dmitry	Khodyakov
Bee Luan	Khoo
Ban Hock	Khor
Jennifer E.	Khoury
Emmanuelle	Khoury
Jaya S.	Khushalani
Lisa	Kidd
Sean	Kidd
Bridget	Kiely
Hanna	Kienzler
Marie	Kierkegaard
Gladys	Kigozi
Laura	Kihlström
Iwaho	Kikuchi
Ali	Kılınç
Katarzyna	Kiliś‐Pstrusińska
Monique	Kilkenny
Eóin	Killackey
Sarah Louise	Killeen
Anne	Killett
Daniel	Kim
Grace J.	Kim
Hae Won	Kim
Hyejin	Kim
Hyun Kyoung	Kim
Joong‐Seok	Kim
Katherine	Kim
Katherine K.	Kim
Minah	Kim
Sang Yun	Kim
Shinmi	Kim
Sung Reul	Kim
Yeunkyung	Kim
Yunmi	Kim
Kilian	Kimbel
Barbara	Kimbell
Katsuhiko	Kimoto
Jochen	Kindler
Dominic	King
Doug A.	King
Lindsay Yount	King
Lindy	King
Nicole	King
Rachel	King
Tania	King
Aimee	Kingsada
Chinedu Okafor	Kingsley
Shauna	Kingsnorth
Gail A.	Kingston
Tom	Kingstone
Julie	Kinley
Shoji	Kinoshita
Sherazade	Kinouani
Connor J.	Kinslow
Michelle	Kip
Pawel	Kiper
Ramazan	Kıraç
Tara	Kiran
Emma	Kirby
Sarah	Kirby
Inge	Kirchberger
Anne C.	Kirchhoff
Dimitra	Kiritsi
Sue	Kirk
Susan	Kirk
James B.	Kirkbride
Pia	Kirkegaard
Benedicte	Kirkøen
Jonathan D.	Kirsch
Bonnie	Kirsh
Nikolai	Kiselev
Taishiro	Kishimoto
Aygul	Kissal
Caroline	Kistin
George D.	Kitas
Betty A.	Kitchener
Henry	Kitchener
Lisa	Kitko
Georgios D.	Kitsios
Sarah	Kittel‐Schneider
Simon	Kitto
Ingvild	Kjeken
Sofia	Kjellström
Emma	Kjörk
Nadja	Klafke
Evan M.	Kleiman
Augustus	Klein
Olga A.	Klein
Knut‐Inge	Klepp
Keith	Kleszynski
Nolan	Kline
Jennifer	Klinedinst
Julian	Klingbeil
Lisa	Klingelhöfer
Elisabeth	Klinkenberg
Vanessa	Klodnick
Kari	Klungsøyr
Peter	Knapp
Ian	Kneebone
Brandon A.	Knettel
Erin	Knight
Sarah	Knight
Katherine	Knighting
Johannes	Knitza
Leanne K.	Knobloch
Christopher E.	Knoepke
Jennifer	Knopf
Sarah	Knowles
Simon	Knowles
Liam	Knox
Lone	Knudsen
D.	Knutson
Jun	Kobayashi
Peter	Koch
Bharati	Kochar
Agnes	Kocher
Bogda	Koczwara
Tamar	Kodish
Andreas	Koehler
Niek	Koenders
Christopher J.	Koenig
Hans‐Helmut	Koenig
Jan	Koetsenruijter
Christopher	Kofahl
Alexis Coulourides	Kogan
Amy J.	Kogon
Gerald Choon‐Huat	Koch
Marty	Kohler
Pamela	Kohler
Philipp	Kohler
Stefanie	Köhler
Simone	Kohler
Sara Ahola	Kohut
Kelly	Kohut
Binu	Koirala
Maryse	Kok
Albère	Köke
Alper	Köker
Kristina M.	Kokorelias
Ifeoluwapo Oluwafunke	Kolawole
Niina	Kolehmainen
Betty	Kollia
Petros	Kolovos
Aleksandra	Kołtuniuk
Rita	Komalasari
Aravind	Komuravelli
Anna Pefoyo	Kone
Rie	Konno
Hanne	Konradsen
Tzartzas	Konstantinos
Jerzy	Konstantynowicz
Evangelos	Kontopantelis
Pia	Kontos
Ischelle Jing Yuan	Koo
Rudolf	Kool
Lyndsie M.	Koon
Harriet	Koorts
Ida	Korfage
Gülbahar	Kormaz Aslan
Christina S.	Korownyk
Radha	Korupolu
Daphne	Kos
Sanna	Koskinen
Marcin	Kosmalski
Ellen Ernst	Kossek
Oyéné	Kossi
Kasia	Kostyrka‐Allchorne
Tomomi	Kotani
Dipak	Kotecha
Yasuhiro	Kotera
Yasuhiro	Kotera
Anita	Kothari
Mohit	Kothari
Daniel	Kotz
Ali	Kouhi
Sofia	Koukouli
Toula	Kourgiantakis
Eleni	Koustriava
Dimitrios	Koutoukidis
Katharina	Kovacs Burns
Maksymilian	Kowalik
Betul	Kozanhan
Maria	Kózka
Kasia	Kozlowska
Natascha	Kozlowski
Jeff A.	Kraakevik
Paul	Krabbe
Lotte	Krabbenborg
Thomas	Krafft
Daniel	Kramer
Jennifer	Kraschnewski
Tal	Krasovsky
Ines	Krass
Karolin	Krause
Roni	Kraut
Christian	Krauth
Richard	Kravitz
Adalbert	Krawczyk
Ulrika	Kreicbergs
Iwona	Krela‐Kaźmierczak
Eric P.	Krenning
Irene	Kretchy
Lillian	Krikheli
Sunil	Kripalani
Rahul	Krishnamurthy
Meinir	Krishnasamy
Heggdal	Kristin
Sigfus	Kristinsson
Agnete E.	Kristoffersen
Afranio	Kritski
Teppo	Kroger
Hans	Kroon
Marlou de	Kroon
Janet	Krska
Nicolas	Krucien
Nicolas	Krucien
Katja	Krug
Sebastian	Krug
Kasper	Kruithof
Margaret E.	Kruk
Maria	Krutikov
Karoilna	Krysinska
	Ku
Li	Kuang
Karolina	Kuberska
Anastacia	Kudinova
M. Ellen	Kuenzig
Maja	Kuharic
Katja	Kühlmeyer
Randall	Kuhn
Georg	Kuhn
Yvonne	Kuipers
Omar	Kujan
Małgorzata	Kujawska
Polina	Kukhareva
Vaitheeswaran	Kulothungan
Hideki	Kumagai
Amit	Kumar
Manisha	Kumar
Nanjesh	Kumar
Saravana	Kumar
Vinayak Anand	Kumar
Narmadan	Kumarasamy
Anastasia	Kunac
Marleen	Kunneman
Marleen	Kunneman
Marleen	Kunneman
Filiz	Kunuroglu
Setor	Kunutsor
Nicholas I‐Hsien	Kuo
Shu‐Yu	Kuo
Nuriye	Kupeli
Gilad	Kuperman
Aylin	Kurt
Silke	Kuske
Nancy	Kusmaul
Isabelle S.	Kusters
Agnieszka Zogata	Kusz
Anne	Kuusisto
Linda A. H.	Kvæl
Liv	Kvalvik
Marit	Kvangarsnes
Linda	Kwakkenbos
Timothy	Kwok
Simona C.	Kwon
Simona C.	Kwon
Mimmie	Kwong
Kelly A.	Kyanko
Maya	Kylén
Mikko	Kytö
Laura	Kytövuori
Rebecca	L. Pearl
Katherine	L. Possin
Michelle	L. Townsend
Jennifer	L. Hefner
Patrece	L. Joseph
Douglas	L. Kriner
Kerri	L. LaRovere
Vanessa	L. Merker
Anne	L. R. Schuster
Nathan	L. Vanderford
Jean‐Pierre	Laake
E‐Liisa	Laakso
Tracey‐Lea	Laba
Guy	Laban
Maude	Laberge
Rebecca E.	Lacey
Lisa	Lachance
Nathan	Lachowsky
Ashley	Lacombe‐Duncan
Jennifer	Lacy‐Nichols
Emma	Ladds
Ricardo	Ladeiras‐Lopes
Keren	Ladin
Jennifer	Lafata
Nadine	Lages
Durjoy	Lahiri
Michelle	Lai
Rebekah	Laidsaar‐Powell
M.	Laimer
M. K.	Laine
Elizabeth Ann	Laird
Yvonne	Laird
Peter Laszlo	Lakatos
Alison M.	Lake
Richard	Lakeman
Lincy S.	Lal
Mirza	Lalani
Nasreen	Lalani
Pieterbas	Lalleman
Joanne	Lally
Joan Gabrielle	Lalor
Simon Ching	Lam
Tai Pong	Lam
Wendy	Lam
Sallie	Lamb
Kelly	Lambert
Veronica	Lambert
Hannah	Lambie‐Mumford
Marie‐Eve	Lamontagne
Marie‐Eve	Lamontagne
Gary	Lamph
Gary	Lamph
Giovanni	Lamura
Victoria	Land
Kalle	Landerholm
Giulia	Landi
Markus A.	Landolt
Frank	Lane
Julie	Lane
Riki	Lane
Stuart	Lane
Berit	Lange
Rael T.	Lange
Brittney S.	Lange‐Maia
David A.	Langer
Mirjam	Langeveld
Aili	Langford
Aisha T.	Langford
Ann	Langius‐Eklöf
Joseph	Langley
John	Lantos
Sylvie	Lantuejoul
Ana Belén del Río	Lanza
Roberta	Lanzillo
Annie	Lapointe
Catherine	Laporte
Julia M.	Lappin
Mariela Loreto	Lara‐Cabrera
Liliana	Laranjo
David T.	Lardier
Meri	Larivaara
Nadine	Larivière
Louise	Larkin
Michael	Larkin
Sarah	Larkins
Ebenezer	Larnyo
Helena	Laroche
Rasmus Tolstrup	Larsen
Marie	Larsen
Ingrid	Larsson
Markus	Larsson
Francene	Larzelere
Cecilia	Lastra
Youssef	Latifeh
Corine	Latour
Emily	Lattie
Jennifer Y. F.	Lau
Vincent Issac	Lau
Sandra B.	Lauck
Lucas	Lauder
Fiquet	Laure
Christina A.	Laurenzi
Jacqueline	Laures‐Gore
Guy	Laureys
Steven	Laureys
Giuseppe Lauria	Lauria
Rosapia	Laurogrotto
Verna	Lavender
Kate	Laver
Anna	Lavis
Josee	Lavoie
Calvin	Law
Rebecca‐Jane	Law
Folake	Lawal
Belinda	Lawford
Peter	Lawlor
Caroline	Lawlor
Sharon	Lawn
Maggie	Lawrence
Robyn L.	Lawrence
Vanessa	Lawrence
Zoe	Lawrence
Anna	Lawrence‐Jones
Lindsey	Lawson
Julia	Lawton
Rebecca	Lawton
Natasha	Layton
Ioulietta	Lazarou
Jeffrey V.	Lazarus
Maria	Lazo‐Porras
Delphine	Le Goff
Marielle	Le Mat
Leire	Leache
Letícia Ferro	Leal
Michael	Lean
Yvan	Leanza
Mark	Learmonth
Owen P.	Leary
Olivia	Leavy
Jack	Leayr
Annie	LeBlanc
Judith	Leblanc
Lydie A.	Lebrun‐Harris
Andrea	Lechiancole
CaRolina	Lechosa Muñiz
George	Leckie
Bernard T.	Lee
Cheuk Kwong	Lee
Danbi	Lee
Heeyoung	Lee
Hooyeon	Lee
Hyejin	Lee
Hyojin	Lee
Janet Y.	Lee
Jay M.	Lee
Juhee	Lee
Jung‐Ah	Lee
Kyung Hee	Lee
Lena J.	Lee
Linda	Lee
Phil Hyu	Lee
Ping‐Yein	Lee
Sejeong	Lee
ShiuYu	Lee
Soo‐Kyoung	Lee
Suk‐jeong	Lee
Ye‐Seul	Lee
Yew‐Kong	Lee
Yvonne C.	Lee
Ikjae	Lee
Do‐Youn	Lee
Pak Wing	Lee
Szu‐Ping	Lee
Shelley	Lees
Jenny	Leese
Barbara L. van	Leeuwen
G. Tyler	Lefevor
France	Légaré
France	Légaré
Carlo	Leget
Johann	Lehrner
Kelli	Lehto
Wang	Lei
Eduardo A.	Leiderman
Julia	Leinweber
Yago	Leira Feijóo
Ulrike	Leiss
Ian P.	Leistikow
Hércules Ribeiro	Leite
Aliénor	Lemieux‐Cumberlege
Ferew	Lemma
Sabine	Lemoyne
Heidi	Lempp
Primrose	Lentin
Trevor A.	Lentz
Anthony	Leonard
Beatriz	León‐Salas
Jeffrey J.	Leow
Didier	Lepelletier
Michael J.	Lepore
A.	Leppin
Aaron	Leppin
Catherine	LePrevost
Pierre	Lequin
Ondrej	Lerch
Harold	Lesch
Gavin	Leslie
Myles	Leslie
Audrey	L'Esperance
David	Lessard
Ethan G.	Lester
Connie	Lethin
Laurent	Letrilliart
Agnes	Leu
Tiffany I.	Leung
Cindy	Leung
Mei‐yung	Leung
Geraline L.	Leusink
Rosella	Levaggi
Kate M.	Levett
April R.	Levin
Anthony	Levitt
Shelly	Levy‐Tzedek
Allison	Lewinski
A.	Lewis
Adam	Lewis
Cara	Lewis
Cara L.	Lewis
Celine	Lewis
Harriet	Lewis
Joanne	Lewis
Krystina B.	Lewis
Martyn	Lewis
Neil A.	Lewis
Penny	Lewis
Sophie	Lewis
Stephen P.	Lewis
Roberto	Lewis‐Fernandez
Marquita W.	Lewis‐Thames
Zakkoyya H.	Lewis‐Trammell
Ann	Ley
JoAnna K.	Leyenaar
Juan M.	Leyva‐Moral
Lina	Li
Mengmeng	Li
Bin	Li
Bin	LI
Chengyue	Li
Haitao	Li
Hao	Li
Hongmin	Li
Jingbo	Li
Jinshuo	Li
Ka	Li
Kaigang	Li
Linda	Li
Linda Chelan	Li
Madeline	Li
Ming	Li
Minghui	Li
Ming‐Qing	Li
Shirley Xin	Li
Shunping	Li
Su‐Xia	Li
Wenjing	Li
Xia	Li
Xin	Li
Zhi	Li
Kristin	Liabo
Cuiying	Liang
Xiaojie	Liang
Yizhi	Liang
Yue	Liao
Sok Ying	Liaw
Elisa	Liberati
Jacki	Liddle
Clare E.	Liddy
Barry	Liden
Sarah C.	Lidstone
Hanne C.	Lie
Helene	Lie
Rebecca	Lieberman
Sarah	Liebherz
Daniel	Liebzeit
Aart C.	Liefbroer
Su May	Liew
Svante	Lifvergren
Sapfo	Lignou
Sapfo	Lignou
Kelly de	Ligt
Claudio	Liguori
Robert	Likic
Maria	Liljeroos
James B.	Lilleker
Daniela	Lillekroken
Hung	Lillian
Crystal S.	Lim
Jae‐young	Lim
Lyn‐Li	Lim
Khatijah	LIm Abdullah
Carlos Augusto de Mendonça	Lima
Regina Aparecida Garcia de	Lima
Felix	Limbani
Ashleigh	Lin
Chung‐Ying	Lin
Frances	Lin
John	Lin
Michelle P.	Lin
Shen (Lamson)	Lin
Shuanglan	Lin
Yannan	Lin
Lewei	Lin
Berit	Lindahl
Magnus	Lindberg
Sebastian	Lindblom
Brooke	Linden
Karolina	Linden
Pirjo	Lindfors
Anja	Lindig
Lee Ann	Lindquist
Sally	Lindsay
Cecilia	Lindsjö
Nadine	Linendoll
Nicholas	Linfoot
Jonathan	Ling
Mathangee	Lingam
Gareth	Lingham
Amy	Linsky
Katarzyna	Lion
Christina	Liossi
Ellen	Lipman
Kate	Lippiett
Ruby	Lipson‐Smith
Ruby	Lipson‐Smith
Ellen	Lipstein
Wendy	Lipworth
Karin	Lisspers
Ian	Litchfield
Julian	Little
Virna	Little
Donna	Littlewood
Kristin	Litzelman
Chaojie	Liu
Chelsea	Liu
Danping	Liu
Hueiming	Liu
Jesse	Liu
Megan F.	Liu
Xiaoguang	Liu
Xinchun	Liu
Maria	Livanou
Polly	Livermore
Jennifer A.	Livingston
Patricia	Livingston
Pablo	Lizana
Lucylynn	Lizarondo
Gwynnyth	Llewellyn
Joy	Llewellyn‐Beardsley
Joan	Llobera
Javier	Llorca
Hywel	Lloyd
Jane	Lloyd
Melanie	Lloyd
Elizabeth	Lobb
Fiona	Lobban
Michelle	Lobchuk
Elton	Lobo
Dara L.	LoBuono
Alexandre Andrade	Loch
Johanna	Löchner
Merilyn	Lock
Abigail	Locke
Craig	Lockwood
Joanna	Lockwood
Louise	Locock
Stacy	Loeb
Nele	Loecher
Sabine	Loeffert
Julia	Loewenthal
Tyler J.	Loftus
Jeongok G.	Logan
Alvona Zi Hui	Loh
Kah Poh	Loh
Matthias	Löhle
Katharina	Löhr
Samantha M.	Loi
Sofia	Loizou
Gururaj Harinahallo	Lokesh
Nicoline	Løkken
Cynthia	Lokker
Zerina	Lokmic‐Tomkins
Amali	Lokugamage
James	Lomas
C.	Lombard
Richard	Lombard‐Vance
Janet	Long
Tracy	Long‐Sutehall
Gunilla	Lönnberg
Jonathan Han	Loong Kuek
Floor C.	Loonstra
Emily W.	Lopes
Snehal	Lopes
Rosa	López‐Gigosos
José Francisco	López‐Gil
Nicole E.	Lorenzo
Paula	Lorgelly
Luis	Loria‐Rebolledo
Claudio Di	Lorito
Elsa	Lorthe
Susan	LoRusso
Andres	Losada
Nneka	Lotea Ifejika
Robyn	Lotto
Gemma	Louch
M. Diane	Lougheed
Alexander V.	Louie
Adetola	Louis‐Jacques
Svetla	Loukanova
David W.	Lounsbury
Padideh	Lovan
Camilla	Lovvik
Lee‐Fay	Low
David John	Lowe
Elizabeth D.	Lowenthal
Linda Pax	Lowes
Emily	Lowthian
Estíbaliz	Loza
Lin	Lu
Qiuchen	Lu
Shurong	Lu
Xinyuan	Lu
Yanming	Lu
David R.	Lubans
Dan	Lubman
Jennifer A.	Lucas
Robyn	Lucas
Todd	Lucas
Federica	Lucivero
Peter	Lude
Tana	Luger
Carla	Luguetti
Florence	Lui
Gloria	Lui
José	Luis Cuesta‐Gómez
Julie	Luker
Angela F.	Lukowski
Eva	Luksaite
Astri	Lundervold
Jennifer	Lundine
Yona	Lunsky
Jake	Luo
Deborah	Lupton
Lely	Lusmilasari
Elizabeth A.	Luth
Jennifer	Lutomski
Barbara J.	Lutz
Binghui	Lv
Courtney R.	Lyles
Elizabeth	Lynch
Fiona	Lynch
Jennifer	Lynch
Hilda Bø	Lyng
Fiona	Lynn
Anthony	Lyons
Georgios	Lyratzopoulos
Gandy	M.
Nicole Rankin	M.
Victoria Flood	M.
Mandy	M. Archibald
Lukas	M. Fuchs
Darrell	M. Gray II
Ellen	M. McCreedy
Laura	M. Perry
Mahendra	M. Reddy
Patrick	M. Bossuyt
Cynthia	M. Boyd
Amanda	M. Clifford
Jarrod	M. Ellingson
Andrea	M. Haqq
Ashley	M. Hyde
Claire	M. Keene
Clare	M. McCann
Jacqueline	M. McGinley
Alison	M. Mudge
Sean	M. Murphy
Manan	M. Nayak
Jenny	M. Norlin
Sharmilee	M. Nyenhuis
Jonaid	M. Sadang
Jaclyn	M. W. Hughto
Richard	Ma
Xiaorong	Ma
Yanan	Ma
Yong	Ma
Claar D. van der	Maarel‐Wierink
Christopher Ryan	Maboloc
Olivia	Mac
Margaret	MacAndrew
Zachary	Macchi
Ilaria	Maccora
Marilyn	Macdonald
Sheila	Macdonald
Arlene G.	MacDougall
Arlene Greer	MacDougall
Hannah	MacDougall
Katie	MacEntee
Sarah R.	MacEwan
Steve	MacGillivray
Alexander James	MacGregor
Pedro M.	Machado
Karen	Machin
Fortunate	Machingura
Ángel Ramos	Macías
Julian Piotr	Maciaszek
Emma	MacIver
Amanda	Mack
Crystal	MacKay
Lynette	Mackenzie
Shylie	Mackintosh
Fiona	Macklchan
Alice	MacLean
Joanna E.	Maclean
Graeme	MacLennan
Martha	Macleod
Alexandra	Macmillan
Antonio	Maconi
Catherine	MacPhail
Karen	MacPherson
Peter	MacPherson
Jennifer	MacRitchie
Mina	Madan
Lucy	Maddox
Michelle	Maden
Octavia‐Luciana	Madge
Neha	Madhiwalla
Debbie Y.	Madhok
Aubrey Spriggs	Madkour
Kristoffer Panduro	Madsen
Walter	Maetzler
Elisa M.	Maffioli
Marina	Maffoni
Eric	Mafuta
Eunice	Magalhães
Rosa	Magallón‐Botaya
Mark Jesus M.	Magbanua
Astrid	Magele
Gkikas	Magiorkinis
A.	Magnoss
Laio	Magnoss
Lina	Magnusson
Marina	Magrey
Natasha	Magson
Tessa	Maguire
Abhishek	Mahajan
Sangeeta	Mahajan
Fayez	Mahamid
Syed Sarosh	Mahdi
Humaira	Maheen
Lynne	Maher
Paul J.	Maher
Marc‐Andre	Maheu‐Cadotte
Ziyad Riyad	Mahfoud
Abela	Mahimbo
Asos	Mahmood
Humaira	Mahmood
Ozayr	Mahomed
Lyn	Mahoub
David W.	Maidment
Ian	Maidment
Marja‐Liisa	Mailend
Lara	Maillet
Jacqueline L.	Mair
Frances	Mair
Umair	Majid
Winnie	Mak
Danii	Makarov
Kristin G.	Maki
Hyuma	Makizako
Shelly	Makleff
Yuwan	Malakar
Sabiha	Malek
Simon	Malfait
Prahbhjot	Malhi
Chetna	Malhotra
Rahul	Malhotra
Ahmad Azam	Malik
Saloni	Malik
Shahab Alam	Malik
Bradley A.	Malin
Lucas	Malla
Anastasia	Mallidou
Ailish	Malone
Colin	Malone
Beth	Malory
Mats	Målqvist
Kirsti	Malterud
Helena C.	Maltezou
Hadii M.	Mamudu
Laxmaiah	Manchikanti
Julien	Mancini
Julien	Mancini
Patrizia	Mancini
Pravat K.	Mandal
Thomas	Mandl
Belinda N.	Mandrell
Della	Maneze
Jennifer	Manganello
Elisabeth	Mangrio
Courtney W.	Mangus
Kiran	Manhas
Elizabeth	Manias
Logan	Manikam
Manjula	Manikandan
Kathleen	Manipis
Maria	Maniscalco‐Feichtl
Veena	Manja
Kim	Manley
Kim	Manley
Kaisa	Mannerkorpi
Joseph C.	Manning
Molly	Manning
Melissa L.	Mannion
Jittima	Manonai
Elise	Mansfield
Elizabeth	Mansfield
James	Mansi
Razan	Mansour
Jill	Manthorpe
Elisa	Mantovani
Emmanuel	Manu
Joel	Manzi
Myfanwy	Maple
Debbie L.	Marais
Inocencio	Maramba
Rachel	Marans
Gelkopf	Marc
Gary E.	Marchant
Emily	Marchant
Giulia	Marchetti
Laura	Marciano
Laura	Marciano
Roberto	Marcone
Jorge Marcos	Marcos
Afrodita	Marcu
Kanchan	Marcus
Marjan	Mardani‐Hamooleh
Carin	Maree
Johanna Elizabeth	Maree
Claire E.	Margerison
Monica	Margoni
Mette	Margrethe Løwe
Silvia Francesca	Maria Pizzoli
Musso	Mariacristina
Smaragdi	Marinaki
Katitza	Marinkovic
Jennifer	Marino
Eduardo	Mariño
Gino	Marioni
Sarah	Markham
Beth	Marks
Elizabeth	Marks
Nancy	Marlett
Charles	Marley
Laura	Marlow
Srinivas	Marmamula
Lena	Marmstål Hammar
Elena	Marques‐Sule
Marisa E.	Marraccini
Ruth Ann	Marrie
Christina N.	Marsack‐Topolewski
Matthias	Marsall
Andrew	Marsh
Claire	Marsh
Lynne	Marsh
Alison	Marshall
Amy	Marshall
Andrea	Marshall
Cassondra	Marshall
Deborah	Marshall
M.	Marshall
Sarah Alexandra	Marshall
Marta	Marsilio
Terry	Martens
Jennifer L.	Marti
Charlene	Martin
Angela	Martin
Denis	Martin
Graham	Martin
Jennifer H.	Martin
Joanna	Martin
Kimberly J.	Martin
Lindsey	Martin
Rachelle	Martin
Iveris L.	Martinez
Maria Soledad	Martinez
Noelia	Martínez‐Molina
Maria Luisa	Martino
Chantal	Martin‐Soelch
Alessio	Martucci
Toshiyuki	Marutani
Francoise A.	Marvel
Christine	Mary Hallinan
Emanuele	Marzetti
Helena	Marzo‐Ortega
Franco	Mascayano
Vasilios G.	Masdrakis
Sarah C.	Masefield
Stephen	Mason
Abbey R.	Masonbrink
Umair	Masood
Reza	Masoodi
Maurizio	Massaro
Barbara	Masser
Greta	Massetti
Oliver T.	Massey
Caroline	Massot
Agnieszka	Mastalerz‐Migas
Daniel	Masterson
Stefanos	Mastrotheodoros
Shoichi	Masumoto
Maria	Matarese
Karen	Mate
Kedar K. V.	Mate
Begoña	Matellanes
Sethunya	Matenge
Joaquin	Mateo
Kayla	Matheus
Katherine D.	Mathews
Elspeth	Mathie
Liv	Mathiesen
Liv	Mathiesen
Elaine	Mathieson
Manu	Mathur
Sonia	Mathur
Moliehi	Matlala
Joseph K. B.	Matovu
Yui	Matsuda
Eiji	Matsuura
María G.	Matta
Peter	Mattei
Evan W.	Matthews
Rachel	Matthews
Stephen	Matthey
Marianne	Matthias
Giovanni	Mattia
Soeren	Mattke
Meghan	Mattos
Kerstin	Mattukat
Łukasz	Matusiak
Louis S.	Matza
Rodolfo	Mauceri
Rapeephan R.	Maude
Twiddy	Maureen
Petra	Maurer‐Karattup
Heleen	Maurice‐Stam
Lauren	Mawn
Rebecca	Mawson
Tulio	Maximo
Christina	Maxwell
Folasade P.	May
Sylvie Le	May
Tamara	May
Jutta S.	Mayer
Anna G.	Mayhew
Chris	Maylea
Fiona	Mayne
Evan	Mayo‐Wilson
Hannah L.	Mayr
Monir	Mazaheri
Marcellus	Mbah
Elliot	Mbunge
Kevin	Mc Namara
Joanna	McAdam
Mara A.	McAdams‐DeMarco
Sarah	McAllister
Sue	McAllister
Susan	McAndrew
Caitlin	McArthur
Corey	McAuliffe
Eilish	McAuliffe
Fionnuala M.	McAuliffe
Karen	McBride‐Henry
Erin	McCabe
Kirsten	McCaffery
Nikki	Mccaffrey
Janya	McCalman
Edward	McCann
Tamara	McCarron
Kristen	McCarter
Gerard	McCarthy
Hazel	McCarthy
Sharon L.	McCartney
Mary	McCauley
Darren	McCausland
Andrea	Mccloughen
Doreen	McClurg
David	McConnell
Natalie	McCormick
Christine	McCourt
Courtney E.	McCracken
Julie S.	McCrae
Janet	McCray
May	McCreaddie
Karen	McCreesh
Marie	McCullagh
Bradley	McDaniels
Meghan	McDarby
Fiona	McDermott
Orii	McDermott
Janet	McDonagh
Emily G.	McDonald
Clare	McDonald
Katherine	McDonald
Ruth	McDonald
Pearl	McElfish
Gemma	Mcerlean
Rachel	McEvoy
Alison	McEwen
Alison	Mcfaddon
Soroya Julian	McFarlane
Anne	McFarlane
Lisa	McGarrigle
Jennifer	McGaughey
Bronwyn	McGill
Gerry	McGivern
Eleanor	McGlinchey
Lucy	McGoron
Ruth	McGovern
Aisling	McGrath
Jacqueline M.	Mcgrath
Nuala	Mcgrath
Gordon	McGregor
Treasure	McGuire
Gretl	McHugh
Susan	McInnes
Daniel I.	McIsaac
Adam	Mckay
Fiona	McKay
Kathy	McKay
Anne Chevalier	McKechnie
Martin	McKee
Lois	McKellar
Odessa	McKenna
Verna	McKenna
Fiona	McKenzie
Mick	McKeown
John	McKie
Shannon	McKinn
Aidín	McKinney
Iain	McKinnon
Ulla	McKnight
Sarah	Mclachlan
Laurie	McLay
Alan	McLean
Caitlan	Mclean
Jenny	McLeish
Jenny	McLeish
Jordana	McLoone
James	McMahon
Naoimh E.	McMahon
Darcy Jones	McMaughan
Brian	McMillan
Sara	McMillan
Christel	McMullan
Anne	McMurray
Rosa	McNamara
Harry	Mcnaughton
Ryan	McNeil
Patricia	McNeilly
Patricia	McNeilly
Ruth	McNemanin
Joanne	McPeake
Steven	McPhail
Amy	McPherson
Colleen M.	McQuown
Theodore R.	McRackan
Jackie	McRae
Jackie	McRae
Lauren	McTier
Laura	McWhirter
Daniel F.	Mcwilliams
Lorna	McWilliams
Cathy	Meade
Oonagh	Meade
Angela	Meadows
Graham	Meadows
Fanuel	Meckson Bickton
Arielle J.	Medford
Roberto	Mediavilla
Laura	Medina‐Perucha
Courtney L.	Meehan
Rebecca	Meehan
Joerg J.	Meerpohl
Mira	Meeus
Krista	Mehari
Salima	Meherali
Ramtin	Mehraram
Yongxia	Mei
Rozanna	Meijboom
Judith M. M.	Meijers
Andreas	Meisel
Ramona	Meister
Michael Benedict A.	Mejia
Inge	Melchior
Jeanette	Melin
Ben	Meller
Jemima E.	Mellerio
Jemima E.	Mellerio
Svein Ivar	Mellgren
Débora Gusmão	Melo
Pedro	Melo
Marco	Meloni
Samuel	Menahem
Prudence	Menard
Felipe Augusto dos Santos	Mendes
Shanthi	Mendis
Justino	Mendoza
Matthew	Menear
Melody	Menezes
Zelalem	Mengesha
Zelalem	Mengesha
Anne Marit	Mengshoel
Devidas	Menon
Viaks	Menon
Adwoa	Mensah
Alessandro	Mentasti
Cristian	Mercê
Stewart	Mercer
Reshma Aziz	Merchant
Liesbeth	Mercken
Mario	Merialdi
Thomas V.	Merluzzi
Eamon	Merrick
Hannah	Merrick
Sally	Merry
Hanneke	Merten
Eva‐Maria	Merz
Bertalan	Meskó
Alice	Mesnard
Anna	Metcalfe
Lotte	Meteyard
Abigail	Methley
Alexandra	Metse
Allison	Metz
Margot	Metz
Silke	Metzelthin
Gloria	Metzner
Yvette	Meuleman
Fraukje E. F.	Mevissen
Amary	Mey
Turgut	Meydan
Ashley	Meyer
Carly	Meyer
Ilan H.	Meyer
Sandra	Meyer‐Moock
Delky	Meza‐Valderrama
Roberto	Mezzina
Pisters	M. F.
Jahanara	Miah
Petty	Michael
Brian	Michael Draper
Maria	Michail
Erin	Michalak
Bernhard	Michalowsky
Kalina J.	Michalska
Kaleb	Michaud
Chantal	Michel
Hilary K.	Michel
Giorgia	Michelini
Erin D.	Michos
Amanda M.	Midboe
Jo	Middlemass
Laura	Middleton
Lyn	Middleton
Nick	Midgley
Julie	Midtgaard
Koen	Migchelbrink
Arn	Migowski
Antonio	Miguel‐Cruz
Rasa	Mikelyte
Ellen M.	Mikkelsen
Antonina A.	Mikocka‐Walus
Andrew	Milat
Benjamin	Milbourn
Kimberley G.	Miles
Shannon R.	Miles
Andrew	Miller
Colette	Miller
Evonne	Miller
Sarah J.	Miller
Chris	Miller‐Rosales
Kristi	Milley
Catherine	Mills
Jesse N.	Mills
George R.	Milne
Linda	Milnes
Rachel	Milte
Alyssa	Milton
Akira	Mima
Laurel	Mimmo
Christina	Minami
Tafadzwa	Mindu
David	Ming
Nadia	Minian
Barbara	Mintzes
Pietro	Minuz
Alessandro	Miola
Andrea	Mira Olivos
Adralan	Miraei
Rose	Miranda
Jelena	Mirkovic
Evelina M.	Miropolsky
Gita D.	Mishra
Shiraz I.	Mishra
Joanna	Mishtal
Shivani	Misra
Caroline	Mitchell
Caroline M.	Mitchell
Gary	Mitchell
Geoffrey	Mitchell
Kirstin	Mitchell
Sarah	Mitchell
Susan	Mitchell
Janis	Miyasaki
Roobol	M. J.
Philipa	Mladovsky
Nosimilo	Mlangeni
Amy R.	Mobley
Pilar	Modamio
S.	Modanl
Sara	Modig
Perpetua	Modjadji
Robiana	Modjo
Mantji Juliah	Modula
K.	Moeller‐Saxone
Suzanne	Moffatt
Victor	Mogre
Rosmawati	Mohamed
Salbiah	Mohamed Isa
Masoud	Mohammadnezhad
Rouidi	Mohammed
Akshay	Mohan
Nathaniel	Mohatt
Mohammadreza	Mohebbi
T.	Moholdt
Wiebke	Mohr
Cynthia M.	Mojica
Freda	Mold
Sue	Molesworth
Pablo	Molina‐Garcia
Mauricio	Molinari‐Ulate
Matthew	Molineux
Sandra	Moll
Tatyana	Mollayeva
Rikke Elmose	Mols
Andrew	Monaghan
Ken	Monaghan
Renée	Monchalin
Amir Abbas Tahami	Monfared
Suneeta	Monga
Helen	Monkman
Ilaria	Montagni
Jane	Montague
Amanda	Montalbano
Eva	Montané
Anna‐Rae	Montano
Claire	Montell
Nicola	Montemurro
Stephanie	Montesanti
Pilar	Montesó
Victor	Montori
Xavier	Moonen
Kim	Mooney‐Doyle
Darren	Moore
Jane	Moore
Kirsten	Moore
Steven A.	Moore
Timothy	Moore
Gregory P.	Moore
Arifa	Moosa
Stephanie R.	Morain
Ernesto	Morales
Paul	Moran
Galia	Moran
Grace	Moran
Susanna	Morano
Nicola	Morant
Nicola	Morant
Andrew	Morden
Kimberly D.	Morel
Evalotte	Morelius
Carmen	Moreno
Ana Maria	Moreno Londono
Antonio R.	Moreno‐Poyato
Cathy	Morgan
Deborah J.	Morgan
Deidre	Morgan
Heather	Morgan
Jenna L.	Morgan
Kerri A.	Morgan
Mark	Morgan
Kohji	Mori
Andrew	Moriarty
Nuccia	Morici
Angela	Morin
Nils‐Halvdan	Morken
Maru	Mormina
Valentina	Moro
Abigail	Morris
Bonny B.	Morris
Brigid	Morris
Christopher	Morris
Heather	Morris
Jacqui	Morris
Megan	Morris
Rachel S.	Morris
Rebecca	Morris
Blaise	Morrison
John M.	Morrison
	Morrow
Alyssa	Morse
Brad	Morse
Elissa	Morse
Maggie	Mort
Liza	Morton
Ali Mohammad	Mosadeghrad
Paola	Mosconi
Ellen	Moseholm
Albine	Moser
Tobias	Moser
Idda	Mosha
Fabiola V.	Moshi
Fabiola	Moshi
Isabel	Mosquera
Gary	Moss
Jennifer L.	Moss
Zahra	Motaghi
Robert	Motl
Joz	Motmans
Keiko	Motokawa
David John	Mott
Nicolas	Mottet
Gregory	Moullec
Alice	M. Oult
David	Mountain
Gail A.	Mountain
Ghassan	Mourad
Ellen	Mowry
Wendy	Moyle
Katie	Moynihan
Jessica	Mozersky
Adam J.	Mrdjenovich
Lilian	Mselle
Asanda	Mtintsilana
Carla	Mucignat‐Caretta
Istvan	Mucsi
Amy S.	Mudano
Axel C.	Muehlbacher
Susanne	Muehlschlegel
Susanne	Muehlschlegel
Evamaria	Mueller
Julia	Mueller
Peter	Muennig
Natacha	Mugeni
Faraz	Mughal
Kenneth K.	Mugwanya
Michael	Muhammad
Michael	Muhammad
Felix	Mühlensiepen
Cassey	Muir
Agurtzane	Mujika
Judith	Mukamurigo
Ferdinand	Mukumbang
Marleen de	Mul
Thomas C.	Mules
Jordan	Mullard
Caroline	Mullen
Cecile	Muller
Sam	Muller
Kai Ivar	Müller
Katharina	Müller
Roy	Müller
C. Daniel	Mullins
Andrea	Mulliri
Gillian	Mulvale
Fehmidah	Munir
Nete	Munk Nielsen
Zachary	Munn
Celia	Muñoz
Ricardo F.	Muñoz
Laura	Muñoz‐Bermejo
Inés	Muñoz‐Paredes
Michelle	Munson
Michelle R.	Munson
Giulia M.	Muraca
M.	Murad
Keiko	Murakami
Louisa	Murdin
Adam B.	Murphy
Eileen F.	Murphy
Fiona	Murphy
Joan	Murphy
Aja	Murray
Ashlee	Murray
Carolyn	Murray
Cynthia	Murray
Elizabeth	Murray
Jenni	Murray
Joanne	Murray
Louise	Murray
Beth	Murray‐Davis
Logan T.	Murry
Padma	Murthi
Shruti	Murthy
Yogesh	Murugan
Sarah	Musa
Khamisi	Musanje
Danielle	Muscat
Kate	Muse
Alessandro	Musetti
Umayya	Musharrafieh
David	Musoke
Murad	Mustafa
Allyson	Mutch
Matt G.	Mutchler
Livhuwani	Muthelo
Moses	Muwanguzi
Anita	M. Y. Goh
Linda	Myerholtz
Phyo	Myint
Girish	N.
Imogen Clark	N.
Lemma Bulto	N.
Titov	N.
Buus	N.
Monika	N. Lind
Candice	N. Selwyn
Jenneken	Naaldenberg
Rania	Nadia
Nupur	Nag
Anil Kumar Mysore	Nagaraj
Nabeelah	Nagdee
Taniya	Nagpal
Deepti	Nagrath
Leena	Nahata
Irmina	Nahon
Aanand D.	Naik
Roshan das	Nair
Toshio	Naito
Motohiro	Nakajima
Meranda	Nakhla
Sanggon	Nam
Aravind Kumar	Namasivayam
Susan	Nancarrow
Joy	Nanda
Arindam	Nandi
Nadia	Nandlall
Karabi	Nandy
Karen	Nanji
Veronica	Nanton
Yonemoto	Naohiro
Hendrik	Napierala
Anand K.	Narayan
Gopalakrishnan	Narayanamurthy
Sarah H.	Nash
Khurram	Nasir
Damoun	Nassehi
Nabil	Natafgi
Patrizia	Natale
Prabhu Manickam	Natarajan
Joseph L.	Nates
Sally	Nathan
Maury	Nation
Kim	Naude
Kelly M.	Naugle
Ajinkya	Navare
Patricia	Navas
Leizl Joy	Nayahangan
M. D.	Naylor
Nabi	Nazari
James	Nazroo
Costase	Ndayishimiye
Anne Victoria	Neale
Ann‐Charlotte	Nedlund
Alexander	Nedopil
Robert	Nee
Lakshmi	Neelakantan
Rashi	Negi
Luca	Negrogno
Ruth D.	Neill
Martina P.	Neininger
Elena	Neiterman
Willianne L. D. M.	Nelen
Laura B.	Nellums
Annmarie	Nelson
LaRon E.	Nelson
Lindsay D.	Nelson
Lonnie	Nelson
Ottar	Ness
Eivind	Ness‐Jensen
Paul G.	Nestor
Lis	Neubeck
Antje	Neubert
Carter	Neugarten
Stefan	Neuner‐Jehle
Sonja	Neuser
Ana Luísa	Neves
Barbara Barbosa	Neves
Philip W. S.	Newall
Katie	Newby
Elaine	Newell
Bronwyn	Newman
Kristina	Newman
Ainsley	Newson
Lisa	Newson
Amanda	Newton
Amanda	Newton
Nicola Clare	Newton
Anna	Newton‐Levinson
Chirk Jenn	Ng
Chirk‐Jenn	Ng
Fowie	Ng
Kwok	Ng
Reuben	Ng
Shamay Sheung Mei	Ng
Olivia Miu Yung	Ngan
Irene	Ngune
H. Q.	Nguyen
Linda	Nguyen
Thu Nam T.	Nguyen
Éidín	Ní Shé
Jennifer	Nicholas
Susanne	Nicholas
Tendongfor	Nicholas
Stuart G.	Nicholls
Michelle	Nichols
Brian D.	Nicholson
Caroline	Nicholson
Charlie	Nicholson
Brooke	Nickel
Ginger E.	Nicol
Christina	Nicolaidis
Jørgen E.	Nielsen
Jörn	Nielsen
Karina	Nielsen
Rune K. L.	Nielsen
Mikko	Niemelä
Henk	Nies
Claire	Nightingale
Julie	Nightingale
Ruth	Nightingale
Nik Daliana	Nik Farid
Alireza	Nikbakht Nasrabadi
Anne Britt Vika	Nilsen
Kerstin	Nilsson
Maria	Nilsson
Sandra Feodor	Nilsson
Danielle	Nimmons
Aurora	Ninfa
Matilde	Nisbeth Jensen
Jeff	Nisker
Giulio	Nittari
Joshua D.	Niznik
Manka	Nkimbeng
Flory	Nkoy
Bev	Noble
James	Nobles
Leticia M.	Nogueira
Saara	Nolvi
Camilla W.	Nonterah
Maria	Noonan
Ismail	Noor
Laura	Nooteboom
Laura A.	Nooteboom
Lena	Nordgren
Annika	Nordin
Anna	Norhammar
Cristina	Noriega
Amruta	Nori‐Sarma
Alyson	Norman
Rebecca	Normansell
Sarah	Northcott
Anne	Norup
Sara	Noruzi
Daniel C.	Norvell
Torunn	Nøst
Thamer	Nouh
Mazen	Noureddin
Elysée	Nouvet
Richard	Nowak
Jane	Noyes
Eric	Nsiah‐Boateng
Fidele	Ntie‐Kang
Pitchaya	Nualdaisri
Rebecca Louise	Nund
Jo	Nunnerley
Michael	Nunns
Roberto	Nuño‐Solinís
Abdullah	Nurul
Kayla	Nuss
Kayla	Nuss
Don	Nutbeam
Sabina	Nuti
Sarah	Nutter
Gertrude	Nyaaba
Mathew	Nyashanu
Jenny	Nyberg
Jens	Nygren
Patrick	Nyikavaranda
Fredrica	Nyqvist
Deirdre	O. Donnell
Jennifer	O'Brien
Georgina Burns	O'Connell
Denise	O'Connor
Holly K.	O'Donnell
Fay	O'Donoghue
Analise	O'Donovan
Denis	O'Mahony
Nicola	O'Malley
Dympna	O'Sullivan
Trish	O'Sullivan
Pippa	Oakeshott
Gabriela R.	Oates
Juno	Obedin‐Maliver
Omar	Obeidat
Stefan	Oberndorfer
Karel	OBrien
Kelly	O'Brien
Kerry	O'Brien
Niki	O'Brien
Kate	Obst
Rose	O'byrne
Austin	O'Carroll
Alicia	O'Cathain
Josephine	Ocloo
Rory C.	O'Connor
Dan	O'Connor
Elodie	O'Connor
Margaret	O'Connor
Meredith	O'Connor
Rachel	O'Conor
Simon J. W.	Oczkowski
Wendy	Oddy
Harrison Lindsay	Odgers
Apondi J.	Odhiambo
Donna H.	Odierna
Donna H.	Odierna
Kieran	O'Doherty
Agricola	Odoi
Catherine	O'Donnell
Catherine A.	O'Donnell
Deirdre	O'Donnell
Owen	O'Donnell
Shane	O'Donnell
Sinead M.	O'Donnell
Emily	O'Dowd
Emma L.	O'Dowd
Elisabeth M.	Oehrlein
Nelly	Oelke
Kellynn Qi Xuan	Oen
Anaeze	Offodile
Eirik H.	Ofstad
Jane	Ogden
Hirofumi	Ogihara
Toshihiro	Ogiwara
Tatiana	Ogourtsova
Ebru Gok	Oguz
Peter	O'Halloran
Jeneva L.	Ohan
Jane	O'Hara
Jane	O'Hara
Kelechi	Ohiri
Anne	Oikarinen
Anneka	Oishi
Emmanuela	Ojukwu
Motohiro	Okada
Sally	O'Keeffe
Daniel	Okeowo
Aizemea	Okojie
Henshaw Uchechi	Okoroiwu
Karen	Okrainec
Trine	Oksholm
Michael S.	Okun
Muideen T.	Olaiya
Ellinor	Olander
Michael	Olasoji
Oladele S.	Olatunya
Sepideh	Olausson
Katarzyna	Olcon
Lieke	Oldenhof
Marcel	Olde‐Rikkert
Ben	Oldfrey
Timothy	Olds
B.	Oliván‐Blázquez
Déborah	Oliveira
Henrique Ceretta	Oliveira
Isabel de Jesus	Oliveira
Juliana S.	Oliveira
Debra Parker	Oliver
Kathryn	Oliver
Sandy	Oliver
Tanya	Olmos‐Ochoa
Anna L.	Olsavsky
Johanna	Olson
Rebecca	Olson
Emma	Olsson
Erik M. G.	Olsson
Lars E.	Olsson
Magdalena	Olszanecka‐Glinianowicz
Olufunto	Olusanya
Oladunni	Oluwoye
Mary	O'Malley
Stefano	Omboni
Wioleta Justyna	Omeljaniuk
Lars Haukali	Omland
Omnia El	Omrani
John D.	O'Neil
Jason J.	Ong
Natalie	Ong
Ju Lynn	Ong
Helen Tosin	Oni
Sandersan	Onie
Sarah S.	Ono
Daniel	Ontaneda
Sylvia A.	Opanga
Annabella	Opoku
Maxwell Peprah	Opoku
Eva‐Maria Wild Née	Oppel
Sian	Oram
Pedro	Ordunez
Claire	O'Reilly
Natasha	O'Reilly
Sharleen L.	O'Reilly
Kate	O'Reilly
Rosaura E.	Orengo‐Aguayo
Hod	Orkibi
Julia	Orkin
Martina	Orlovic
Liz	Ormondroyd
Hannah M.	O'Rourke
Pilar	Ortega
Daisy Ramirez	Ortiz
Raymond Uyiosa	Osarogiagbon
David	Osborn
Eamon	O'Shea
Grace	O'Shea
Fatumo	Osman
Onome Henry	Osokpo
Joan	Ostaszkiewicz
Edoardo Giuseppe	Ostinelli
Katarina	Ostojic
Mina	Ostovari
Anna	Ostropolets
Maria	O'Sullivan
Goetz	Ottmann
Ya‐Nan	Ou
Laurent	Oudre
Abe	Oudshoorn
Kate	Oulton
Tiago Fleming	Outeiro
Ludmila	Ovchinikova
Mathilde M.	Overbeek
Charlotte	Overgaard
Christabel	Owens
John	Owens
Matthew	Owens
Otis	Owens
Amanda	Owen‐Smith
Mayowa	Owolabi
Kazuaki	Oyake
Genko	Oyama
Christine	Oye
Ahmet	Özaslan
İpek Betül	Özçivit
Mehmet	Ozel
Ayşe	Özkaraman
Fatma Özlem	Öztürk
Yasemin Ertaş	Öztürk
Louis	P. Garrison
Eric	P. Moll van Charante
Michele	P. Andrasik
Hilary	P. Bagshaw
Gwendolyn	P. Quinn
Mathew	P. White
Piret	Paal
Markus	Paananen
Davy	Paap
Gonzalo Salazar de	Pablo
Alexander	Pabst
Isabella	Pacchiarotti
Megan	Paceley
Lissa	Pacheco‐Brousseau
Eugenio	Paci
Tanya	Packer
Jon	Packham
Tara L.	Packham
Tasleem	Padamsee
Aasim	Padela
Deborah K.	Padgett
Prianka	Padmanathan
Chi‐Un	Pae
Antonio	Pagan
Valerie J.	Page
Claudia	Pagliari
Madhukar	Pai
Hsiang‐Chu	Pai
Carlos Eduardo	Paiva
Domingo	Palacios Ceña
Lorenza	Palazzo
Alvisa	Palese
Andrea	Palinski
Raffaele	Palladino
Amy S.	Paller
Kirsten	Pallesen
Ana	Palmar‐Santos
Victoria Jane	Palmer
Rocío	Palomo‐Carrión
Omkar D.	Palsule Desai
Rocco	Palumbo
Fan	Pan
Maria	Panagioti
Margarita	Panayiotou
Heather	Panczykowski
Mamata	Pandey
Vinciya	Pandian
Danielle Mari	Panelli
Sanaullah	Panezai
Jing	Pang
Henri	Panjo
Stacey	Panozzo
Teresa	Paolucci
Janet	Papadakos
Davide	Papola
Stan	Papoulias
Monique	Pappadis
Colette	Parai
Nayana	Parange
Manish	Pareek
Justin	Parent
Helen	Paretti
Chanhee	Park
Do Hyun	Park
Eun‐Cheol	Park
Eunhee	Park
Hong‐Jae	Park
Hyejoon	Park
Jeongok	Park
Linda G.	Park
Catriona	Parker
Lauren J.	Parker
Philip	Parker
Richard	Parker
Ron	Parker
Stephen	Parker
Justin	Parkhurst
Justin	Parkhurst
Anne	Parkinson
Lynne	Parkinson
Jim	Parle
Elena	Parmelli
Anna	Parola
Ben	Parry
Ruth	Parry
Christine	Parsons
Judith	Parsons
Liz	Parsons
Susan K.	Parsons
Jeanna	Parsons Leigh
Judith S. L.	Partridge
Stephanie R.	Partridge
Sahdia	Parveen
Sanaz	Parvizi
Hanna Van	Parys
Amedro	Pascal
Zoe	Paskins
Emma	Patchick
Atul	Patel
Neesha	Patel
Pinika	Patel
Sapana	Patel
Sonal	Patel
Debora	Paterniti
Catherine	Paterson
Andrea M.	Patey
Sanghamitra	Pati
Gopal Kumar	Patidar
Lewis W.	Paton
Sandra	Patricia Osorio Galeano
Irini	Patsaki
Brian W.	Patterson
Susan M.	Patterson
R.	Pattinson
Deborah	Patton
Rachel E.	Patzer
Prof. Alberto	Paucar‐Caceres
Priya	Paudyal
Vibhu	Paudyal
L.	Paul
Serene S.	Paul
Slater	Paul
Gebrehiwet	Paulos
Mari Mohn	Paulsen
Gabriela	Pavarini
Sofia	Pavarini
Milena	Pavlova
Juliessa M.	Pavon
Camille	Paynter
Jessica	Paynter
Carl J.	Payton
Maria	Paz Zulueta
Fiona	Pazsa
Stuart	Peacock
Kathryn J.	Peall
Alison	Pearce
Christina Joanne	Pearce
Eiluned	Pearce
Mark	Pearson
Mark	Pearson
Mark	Pearson
Matthew R.	Pearson
Odette	Pearson
Tessa	Peasgood
Cornelia	Pechmann
Lydia H.	Pecker
Stephen	Peckham
Henrik	Pedersen
Rebecca	Pedley
Azucena	Pedraz‐Marcos
Roberto F. E.	Pedretti
Farhad	Peerani
Mariëlle	Peeters
Kirsten	Peetoom
Jessica	Pehlke‐Milde
Casey L.	Peiris
Roshini	Peiris‐John
Sandra	Pelaez
Bárbara	Peleteiro
Wiesje	Pelkmans
Mia	Pellizzer
Niall	Pender
Linda	Peng
Craig	Pennell
Iris‐Katharina	Penner
Mark	Pennington
Thomas B.	Pepinsky
Miguel	Peralta
Armanda	Pereira
Carla M.	Pereira
Rosa M. R.	Pereira
Lidia	Perenc
Kate	Perepezko
Lilisbeth	Perestelo‐Perez
Lilisbeth	Perestelo‐Perez
Lilisbeth	Perestelo‐Pérez
Pamela	Pereyra‐Zamora
FerminaRojo	Pérez
Raquel Bosó	Pérez
Sagrario	Pérez‐de la Cruz
Anna	Pérez‐Aronsson
Mª Virtudes	Pérez‐Jover
Jeanette	Perez‐Ramos
Eleanor M.	Perfetto
Vaios	Peritogiannis
V.	Periyakoil
Iain	Perkes
Yelena	Perkhounkova
Unchalee	Permsuwan
Thomas	Perneger
Julie	Péron
Krista M.	Perreira
Eliane	Perrin
Beth	Perry
Henry	Perry
Meredith A.	Perry
Yael	Perry
Mark	Pertile
Panagiota	Pervanidou
Janette	Perz
Melanie	Pescud
Sarah	Peskoe
Marcia	Pestana‐Santos
Barb	Pesut
Pavlo	Petakh
Bridgette J.	Peteet
Micah	Peters
Sarah	Peters
Morten Aa	Petersen
Daniel S.	Peterson
Elin	Peterson
Mark D.	Peterson
Jennifer	Petkovic
Elisabetta	Petracci
Benoit	Pétré
Mark C.	Petrie
Laura	Petrillo
Julie	Petrin
Katja	Petrowski
Saverio	Petruzzelli
Helen L.	Petsky
Gunn	Pettersen
Anna	Pettican
Julia	Petty
Wilco	Peul
Birgit	Pfaller
Luzia Iara	Pfeifer
Elisa	Pfeiffer
Timo‐Kolja	Pförtner
Atharva	Phatak
Helen	Phelan
Leonel	Philibert
Beth	Philips
Hilde	Philips
Christine	Phillips
Jane	Phillips
Karen P.	Phillips
Patrick	Phillips
Rita	Phillips
Shannon	Phillips
Gregory	Phillips II
Lyn	Phillipson
Alison	Phinney
Denham	Phipps
Million	Phiri
Peter	Phiri
Ratchanok	Phonyiam
Viet‐Hai	Phung
Myra	Piat
Myra	Piat
Philippe	Pibarot
Giorgina B.	Piccoli
Alessandro	Picelli
Susannah	Pick
Anton	Pick
A. Simon	Pickard
Kate	Pickett
Christoph	Pieh
Frédéric B.	Piel
Anna‐Maija	Pietilä
Giada	Pietrabissa
Valerie	Piguet
Karin	Piil
Harri	Piitulainen
Jose	Pijoan
Minna	Pikkarainen
Karen	Pilkington
Elizabeth	Pillay
Sophie	Pilleron
Steve	Pilling
Toby	Pillinger
Kate	Pincock
Vanessa	Pinfold
Any	Ping
Ranyon	Ping
Ana Rita	Pinheiro
Jonathan	Pinkney
Andrew	Pinto
Anna	Pinto
Carla	Pires
Sandra	Pires
Jane	Pirkis
Melissa	Pirrie
Alexandra	Pitman
Gabriele	Pitschel‐Walz
Chiara	Pittalis
Carina	Pittens
Samantha	Pitts
Grasiela	Piuvezam
Olga	Pivovarova‐Ramich
Rafael	Pizarro‐Mena
Claire	Planner
Mirjam	Plantinga
Dietrich	Plass
Chris	Platania‐Phung
Michaela	Plath
Lucy	Platt
Thomas	Platz
Thomas	Pletschko
Jenny	Ploeg
Marika	Plöthner
Michelle	Ploughman
Matthew	Plow
Thomas	Poder
Brianna F.	Poirier
Rachele	Pojednic
Brenda	Poku
E.	Poku
Ohemaa	Poku
Fiona	Poland
Megan	Polden
Sarah	Poletti
Kay	Polidano
Mary C.	Politi
McLean D.	Pollock
Alex	Pollock
Ramesh	Poluru
Marie‐Pascale	Pomey
Georg	Pongratz
Maiken	Pontoppidan
Javad	Pool
Marie	Poole
Norman A.	Poole
Abner Weng Cheong	Poon
Liona C.	Poon
Victor	Pop
Catherine	Pope
Janet E.	Pope
Uwe	Popert
Aaron	Poppleton
Piero	Porcelli
Ashley	Port
Alison	Porter
Jerlym S.	Porter
Jude	Porter
Mari Carmen	Portillo
Robert M.	Portman
Dannielle	Post
Judith A.	Potashkin
Thomas	Potrebny
Beth	Potter
Shelley	Potter
Kevin	Pottie
Annie	Pouliot‐Laforte
Christine	Poulos
Ingrid	Poulsen
Rie Mandrup	Poulsen
Melissa N.	Poulsen
Marie	Pouquet
Nadereh	Pourat
Negar	Pourvakhshoori
Catherine	Powell
Jason	Powell
Phil	Powell
Emma	Power
Karina	Powers
Kelly	Powers
Bunny	Pozehl
Kinga	Pozniak
Chiara	Pozzi
Regina	Praetorius
Vishvesh	Prajapati
Natasha	Prasad
Vibhore	Prasad
Giuliana B.	Prato
Laura	Prato
Zachary	Predmore
Tamar J.	Preminger
Leah	Prencipe
Trisha M.	Prentice
Justin	Presseau
Jennifer	Preston
Robyn	Preston
Nancy	Preston
Claudette	Pretorius
Anna M. H.	Price
Amy	Price
Anna	Price
Eluned	Price
Melanie	Price
Owen	Price
I.	Prichard
Olaf	Prieske
Jette	Primdahl
Samantha	Prime
Alexis	Prince
Tamara	Pringsheim
James	Prior
Yeliz	Prior
Liza	Pristianty
Lesley	Pritchard
Yasmine	Probst
Yasmine	Probst
Judith J.	Prochaska
Laura M.	Prolo
Peter J.	Pronovost
Joanne	Protheroe
Judith	Proudfoot
Livio	Provenzi
Federica	Provini
April	Prunty
Helen	Pryce
Alexandra	Psihogios
Lihui	pu
Jo‐Anne	Puddephatt
Yvonne Marina	Pudritz
Frank	Puga
Wendy	Pugh
Robert	Pugliese
Rebecca M.	Puhl
Laia Maynou	Pujolras
Rahul	Pulimamidi
Cheneal	Puljevic
Jack	Pun
Brittany E.	Punches
Soorej Jose	Puthoopparambil
Zudin	Puthucheary
Zeeshan	Qamar
Suparna	Qanungo
Amir	Qaseem
Shazia	Qayyum
Wei	Qi Koh
Li	Qiao
Hong	Qiu
Pamela	Qualter
Lucas C.	Quarantini
Paula A.	Quatromoni
Ana	Querido
Amélie	Quesnel‐Vallée
Fiona	Quigley
Meaghen	Quinlan
Leah	Quinlivan
Catherine	Quinn
Michael	Quinn
Patrick D.	Quinn
Rosie	Quinn
Sam	Quinn
Terry J.	Quinn
Maria M.	Quiñones
Yina	Quique
Silvia Cristina	Quispe‐Prieto
Nadeem	Qureshi
David Thompson	R.
Pracheth	R.
Danielle	R. Davis
Erin	R. Foster
Juliet	R. H. Wakefield
Kristen	R. Haase
Camille	R. Quinn
Rachel	R. Tambling
Kaamran	Raahemifar
Jehad A.	Rababah
Syed Arman	Rabbani
Linda	Rabeneck
Anna	Råberus
Louise	Racine
Nicole	Racine
Jany	Rademakers
Shellie	Radford
Lalit Kumar	Radha Krishna
Asa	Radix
Anika	Rädke
Toby	Raeburn
Leo De	Raeve
Chiara	Rafanelli
Stéphane	Raffard
Miriam	Rafferty
Ismael	Rafols
Maya I.	Ragavan
Marina	Raguz
Ole	Rahbek
Jean‐François	Rahier
Md. Moshiur	Rahman
Helen	Rainey
Linda	Rainey
Luke Sheridan	Rains
Devan	Rajendran
Chakravarthi	Rajkumar
Dorra	Rakia Allegue
Ndidzulafhi Selina	Raliphaswa
Anna P.	Ralph
Nicholas	Ralph
Elizabeth R.	Ralston
Emily	Ramage
Diego	Ramaioli
Astha	Ramaiya
Gita	Ramdharry
Margareta	Rämgård
Sriram	Ramgopal
Lucie	Ramjan
Haywantee	Ramkissoon
Suvira	Ramlall
Shulamit	Ramon
Francesco	Ramponi
Chhabi Lal	Ranabhat
Antonio	Ranchal‐Sánchez
Cynthia	Rand
Stacey	Rand
Gurch	Randhawa
T. L.	Rangel
Manon	Ranger
Sita	Rani
Hadi	Ranjbar
Soheila	Ranjbaran
Audrey	Rankin
Judith	Rankin
Janice M.	Ranson
Penny	Rapaport
Donna de Levante	Raphael
Tim	Rapley
Frances	Rapport
Charlene	Rapsey
Peter	Rasche
Davide	Rasella
Mikael	Rask
Jeffrey S.	Raskin
Sarah A.	Raskin
Bodil	Rasmussen
Stanisa	Raspopovi
Murad A.	Rassam
Jessica	Rassart
Maryam	Rassouli
Cédric	Rat
Joanne	Rathbone
Cheryl	Rathert
Kendra	Ratnapradipa
Ann‐Kathrin	Rauch
Patrick J.	Raue
Arkiath Veettil	Raveendran
Helen	Rawson
Joel G.	Ray
Simon	Ray
Robin	Ray
Jo	Rayner
David	Raynor
Homie	Razavi
Valentina	Razmovski‐Naumovski
Halley	Read
John	Read
Megan	Readman
Louise	Reagan
Tessa	Reardon
Nicola J.	Reavley
Maija	Reblin
Cristian	Rebolledo
Paola	Rebora
Virginia	Recchia
Vivian	Reckers‐Droog
Jennifer M.	Reckrey
Sandeep	Reddy
Maren	Reder
Julie	Redfern
Bernice	Redley
Michelle	Redman‐MacLaren
Maggie	Redshaw
Sabi	Redwood
Eline	Ree
Lindsey	Reece
Ms Rebecca	Rees
Kathrin	Reetz
Caroline	Reeves
David	Reeves
Kathryn	Refshauge
Sam	Regan de Bere
Krishna	Regmi
Joan	Reibman
Natasha	Reid
Dianne	Reidlinger
Claire	Reidy
Lennart	Reifels
Glòria	Reig‐Garcia
Siobhan	Reilly
A.	Reimer‐Taschenbrecker
Lieke	Reinhoudt‐den Boer
Ulrich	Reininghaus
Ria	Reis
Sari	Reisner
Molly	Remch
Bjørn‐Atle	Reme
Masha	Remskar
Katharine	Rendle
Laoise	Renwick
Erika	Renzi
Linda Amundstuen	Reppe
Julie	Repper
James T.	Reston
Carlos Gómez	Restrepo
Dan	Reuland
Marvin	Reuter
Thijs	Reyniers
John	Reynolds‐Wright
Hamid	Reza Khankeh
Hyekyun	Rhee
Sarah	Rhodes
Fahad	Riaz
Federica	Ribaldi
Cíntia	Ribeiro Martins
Bianca Sakamoto	Ribeiro Paiva
Jorge Javier	Ricarte
Ignacio	Ricci‐Cabello
Laura	Rice
Simon	Rice
Simon M.	Rice
Antonia	Rich
Lauralie	Richard
Dawn	Richards
Dawn P.	Richards
Suzanne	Richards
Alison	Richardson
Emma	Richardson
Jane	Richardson
Noel	Richardson
Daniel	Richardson
Ilana B.	Richman
Jennifer	Richmond
Dirk	Richter
Peggy	Richter
Michelle	Rickett
Debra J.	Rickwood
Adrián	Ricoy Cano
Valery	Ridde
John V.	Rider
Damien	Ridge
Nicola D.	Ridgers
Anna Lisa	Ridolfo
Steffi	Riedel‐Heller
Jana	Riegger
Judith	Rietjens
Anne‐Sophie	Rigaud
Khary	Rigg
Sara	Riggare
Elisha	Riggs
Ivana	Rihova
Mieke	Rijken
Michela	Rimondini
Arja	Rimpela
Sarah	Ringold
Robin	Rinn
Daniel	Rippon
Andrea	Rishworth
Kristin L.	Rising
Heather J.	Risser
Lisa	Riste
Jens Carsten	Ritter
Debra P.	Ritzwoller
Melina	Rivard
Jo	River
Ismar A.	Rivera‐Olivero
Diego	Rivera‐Porras
Amado	Rivero‐Santana
Scott	Rivkees
Katherine	Rizzolo
Pamela	Roach
Kathryn	Robb
Paul	Robben
Glenn	Robert
Brian W.	Roberts
Jane	Roberts
Nicola	Roberts
Shelley	Roberts
Douglas James	Robertson
Eden	Robertson
Jane	Robertson
Noelle	Robertson
Catherine	Robinson
Claire H.	Robinson
Edmondo J.	Robinson
Jackie Anne	Robinson
Jo	Robinson
Katie	Robinson
Sally	Robinson
Anna	Robinson‐Barella
Michael	Robling
Bernt‐Peter	Robra
Kristy	Robson
Rosellen	Roche
Aaron	Rochlen
Erna	Rochmawati
Karen	Rodham
Alessandro	Rodolico
Juliana	Rodolpho
Anabel	Rodriguez
Hector	Rodriguez
Leonor	Rodriguez
Mercedes	Rodríguez
Iago	Rodríguez‐Lago
Merida	Rodriguez‐Lopez
Kenny	Rodriguez‐Wallberg
David	Roe
Susanne	Roehr
Annelieke M.	Roest
Veronique L.	Roger
Anne	Rogers
Anne	Rogers
Baker A.	Rogers
Laura Q.	Rogers
Simon	Rogers
Wendy	Rogers
Stine	Rognan
Young sook	Roh
Noelle	Rohatinsky
Susanne	Röhr
Anne Marie Mork	Rokstad
Abigail	Rolbiecki
Estefania	Roldan
Lutaud	Romain
Fernanda Bittencourt	Romeiro
Verónica	Romero‐Ferreiro
M.	Romero‐Gómez
Katelyn F.	Romm
Sjim	Romme
Kenton L.	Rommens
Maria	Romøren
Elena	Ronda
Elena	Ronda‐Pérez
Nadia M. T.	Roodenrijs
Rizwana	Roomaney
Fátima	Roque
Regis Goulart	Rosa
William E.	Rosa
Margaret	Rosario
Rikki	Roscoe
Diana	Rose
John	Rose
Matthias	Rose
Susannah	Rose
Benjamin	Rose‐Davis
Joseph Gregory	Rosen
Rebecca	Rosen
Zahava	Rosenberg‐Yunger
Joshua D.	Rosenblat
Annika	Rosengren
Summer E.	Rosenstock
Jennifer L.	Rosenthal
Anna	Ross
Jamie	Ross
Jamie	Ross
Jennifer Gunberg	Ross
Kathryn M.	Ross
Robert	Ross
Susan	Rossell
Monica	Rosselli
Gournellis	Rossetos
Roberta Elisa	Rossi
Giampiero	Rossi‐Fedele
Lisa	Rotenstein
Esther	Rothblum
Christine	Rotonda
Maryam	Rouhi
Poli	Roukova
Thomas	Round
Maria	Roura
Hugues	Rousset
Ash	Routen
Pauline	Roux
Brian H.	Rowe
Fiona	Rowe
Ingrid	Rowlands
Campbell S.	Roxburgh
Rajshri	Roy
Shalini	Roy
Jessica	Roydhouse
Tomas	Rozbroj
David C.	Rozek
Ronen	Rozenblum
Linda	Rozmovits
Eric	Rubenstein
Christine	Rubertsson
Lola	Rueda‐Ruzafa
Nicolas	Ruesch
Maria	Ruiz Yanzi
Pedro	Ruiz‐Esteban
Tea Vukušić	Rukavina
Godfrey Zari	Rukundo
Raffaella Ida	Rumiati
Sharyn	Rundle‐Thiele
Alexander	Rushforth
Stephanie	Russ
Alex M.	Russell
Grant	Russell
Sarah	Russell
Stuart	Russell
Andrea	Russo
Jennifer	Russomanno
Alison	Rustagi
Vinod K.	Rustgi
Anna‐Lynne	Ruth Adlam
	Rutkowski
Sarah E.	Rutstein
Frans H.	Rutten
Kirstin	Ruttmann
Assumpta	Ryan
Jean	Ryan
Jennifer L.	Ryan
Jennifer M.	Ryan
Jillian C.	Ryan
Laura	Ryan
Leona	Ryan
Rebecca	Ryan
Sara	Ryan
Sarah	Ryan
Lucie	Rychetnik
ELoise	Ryde
Knud	Ryom
Magdalena	Rzewuska
O'Neill	S.
Jamie	S. Myers
Robert	S. Kerrison
Riitta	Saarela
Markus	Saarijärvi
Milla	Saarinen
George	Saba
Marwan N.	Sabbagh
Wael	Sabbah
Jordi Collet‐	Sabé
Kathryn	Sabella
Séverine	Sabia
Kathryn	Sabo
Lawrence B.	Sacco
Joel	Sadavoy
Stephen E.	Saddow
Euan	Sadler
Sahrai	Saeed
Shazina	Saeed
Mugula Chris	Safari
Nasia	Safdar
Emma	Safstrom
Zachary	Sager
Fatma Uslu	Şahan
Sezgin	Şahin
Eva	Sahlberg‐Blom
Catherine	Said
Cathy	Said
Bandana	Saini
Pooja	Saini
Brodie	Sakakibara
Mohamed	Sakel
Yusuke	Sako
Mozhdeh	Salajegheh
Anna Santos	Salas
Sameh N.	Saleh
Zyad	Saleh
Manuela	Salerno
Allan Robson Kluser	Sales
Hani	Salim
Francesco	Salis
Wheelwright	Sally
Omar Hussein	Salman
Heli	Salmi
Jo	Salmon
Jon	Salsberg
Stacie	Salsbury
Giovanni	Salum
Gemma	Salvadó
Martin	Salzmann‐Erikson
Ludovic	Samalin
Aoun	Samar M.
Waqas	Sami
Alexei	Sammut
Hugues	Sampasa‐Kanyinga
Laura	Sampietro‐Colom
Kritika	Samsi
Laura J.	Samuel
Preethy S.	Samuel
Lauren R.	Samuels
Amanda L.	Sanchez
Delida	Sanchez
Diana T.	Sanchez
Katherine	Sanchez
Kyle	Sanchez
Mercedes	Sánchez
Eleuterio A.	Sánchez Romero
David	Sancho
Ilana Katz	Sand
Shaifali	Sandal
Jane	Sandall
Magnus	Sandberg
Caroline	Sanders
Caroline	Sanders
Tom	Sanders
Margaret	Sandham
Marlene	Sandlund
Tony	Sandset
Ann	Sandvi‐O
Noemí	Sansó
Maria	Sansoni
Andrea	Santamato
Maria	Santana
Gabriella	Santangelo
Sara	Santarossa
Joeke van	Santen
Harini	Santhanam
Iván	Santolalla‐Arnedo
Claudia	Santos
Cristina Peixoto	Santos
Derek	Santos
Helena	Santos
Manuel Filipe	Santos
Ana João	Santos
Seyedeh Belin Tavakoli	Sany
Jennifer	Saper
Elizabeth	Sapey
Bhim Prasad	Sapkota
Syed Imran	Saqib
Srishti	Sardana
Sally	Sargeant
Urmimala	Sarkar
Cristina A.	Sarmiento
Elissa L.	Sarno
Gino	Sartor
David B.	Sarwer
Angie	Sassano
Jeanne	Sassenou
Ferdinando Carlo	Sasso
Prassannah	Satasivam
Rose‐Marie	Satherley
Djaina	Satoer
Davide	Sattin
Jane	Sattoe
Benjamin	Saunders
Bernadette J.	Saunders
Catherine	Saunders
Catherine L.	Saunders
Katie	Saunders
Rob	Saunders
Vicki	Saunders
Eileen	Savage
Carl	Savage
Wilma	Savelberg
Elena	Savoia
Mouna	Sawan
Tyson	Sawchuk
Simon	Sawhney
Kapil	Sayal
Caitlin S.	Sayegh
Jo	Sayers
Rachel	Sayko Adams
Zoe L.	Saynor
Simona	Scaini
Peter	Scalia
Sasha	Scambler
Justin	Scanlan
Katrina	Scarff
Alice	Scavarda
Anthony	Scerri
Charles	Scerri
Laura	Schaap
Andrea L.	Schaffer
Ellika	Schalling
Laura E.	Schanberg
Annelie	Scharf
Tine	Schauer Eri
Fedde	Scheele
Ayden	Scheim
Richard M.	Schein
Lisa	Schelbe
Barbara	Schellenberger
Luíza Buzatto	Schemiko
Martijn	Scherrenberg
Sara	Schiavone
Anita	Schick
Kara	Schick‐Makaroff
Holger	Schiele
Jason	Schiffman
Karen	Schipper
Désirée	Schliemann
Nicolas J.	Schlienz
Karen	Schliep
Karen C.	Schliep
Mette	Schlütter
Peter	Schmidt
Julia	Schmidt
Matthew	Schmidt
Susanne	Schmidt
Jessica	Schmitt
Kai‐Uwe	Schmitt
Sara	Schmitt
Tugce	Schmitt
Julian	Schmitz
Sebastian	Schneeweiss
Carl	Schneider
Carmen Huckel	Schneider
Marie‐Paule	Schneider
Jeffrey	Schnipper
Penelope	Schofield
Jesse D.	Schold
Nicole	Scholes‐Robertson
Isabelle	Scholl
Nadine	Scholten
Gwendolijne	Scholten‐Peeters
Brett	Scholz
Pär	Schon
Mara	Schonberg
Meryam	Schouler‐Ocak
Anette	Schrag
Sarina	Schrager
Andreas Sebastian	Schroeder
Krista	Schroeder
Carlo	Schuengel
Brittany	Schuler
Andreas	Schulte
Annette S. H.	Schultz
Peter	Schulz
Tracy L.	Schumacher
Florian	Schuster
Kara	Schvartz‐Leyzac
Roxana	Schwab
Carolyn E.	Schwartz
Lisa A.	Schwartz
Marc	Schwartz
Rachel	Schwartz
Emma	Schwartzkoff
Jürgen	Schwarze
Alessandro	Sciarra
Emma	Sciberras
Ian	Scott
Jan	Scott
Jason	Scott
Jonathan B.	Scourfield
Juan	Scribante
Paul	Scuffham
Holly	Seale
Adam	Searby
Aidan	Searle
Kim	Sears
Giada	Sebastiani
Antonin	Sebela
Valeria	Sebri
Ernesto M.	Sebrié
Kate	Seear
Jonathan	Segal
Seppe	Segers
Rebecca	Seguin‐Fowler
Zac	Seidler
Deepa L.	Sekhar
Mandeep	Sekhon
Yukari	Seko
Geir	Selbæk
Debbie	Selby
Nura Fitnat	Selcuki
Kate	Sellen
Kath	Sellick
Johann	Sellner
Lucy	Selman
Lucy Ellen	Selman
Rebecca R.	Seltzer
Cherith J.	Semple
Michaela	Senek
Mohammed	Senitan
Konstantin	Senkevich
Nicholas	Senne
Enoch	Sepako
Nombulelo V.	Sepeng
Alexandre	Sepriano
Karen	Sepucha
Lydia	Sequeira
Peter T.	Serina
Valerie	Seror
Kristie	Serota
Pedro	Serrano
Diego	Serrano‐Muñoz
Laurent	Servais
Kathy	Service
Luciana M. S.	Servo
Sanjay	Sevak
Nick	Sevdalis
Jae	Sevelius
Mehmet S.	Sever
Christina	Severinsen
Eithne	Sexton
John Bryan	Sexton
Eruyar	Seyda
Mehmet Akif	Sezerol
Duygu	Sezgin
Gorkem	Sezgin
Hafifa Siddiq	Shabaik
Arash	Shaban‐Nejad
Jahan	Shabnam
Victoria A.	Shaffer
Jai	Shah
Syed Ghulam Sarwar	Shah
Goded	Shahaf
Suzana	Shahar
Rabia	Shahid
Marcus	Shaker
Tom	Shakespeare
Daniel	Shalev
Sherene	Shalhub
Omar M.	Shamieh
Isdore Chola	Shamputa
Hana	Shamsan
Linghan	Shan
Abhishek	Shankar
Adrienne E.	Shapiro
Mulatu Biru	Shargie
A.	Sharif
Sameer	Sharif
Christina	Sharkey
Amit	Sharma
Dileep	Sharma
Nisha	Sharma
Pravesh	Sharma
Saurab	Sharma
Catherine	Sharp
Charlotte	Sharp
Linda	Sharp
Lisa	Sharp
Paul	Sharp
Alison	Shaw
Christopher D.	Shaw
Liz	Shaw
Palma M.	Shaw
Rachel	Shaw
L.	Shay
Elizabeth H.	Shayo
Rod	Sheaff
Laura	Sheard
Heather	Shearer
Katie	Sheehan
Rory	Sheehan
Kaitlin M.	Sheerin
Pamela	Sheffler
Ross	Shegog
Sahar	Shekoohi
Elena	Sheldon
Radley Christopher	Sheldrick
Rachel C.	Shelton
Benjamin	Shemesh
Minxue	Shen
Xinglei	Shen
Heather	Shepherd
Tom	Shepherd
Victoria	Shepherd
Sukhwinder S.	Shergill
Nicolette	Sheridan
Rebecca Syed	Sheriff
Athena D. F.	Sherman
David K.	Sherman
Susan	Sherman
Frances	Sherratt
Denese	Shervington
Merryn	Sherwood
Lili	Shi
Darren	Shickle
Carol	Shieh
Cleveland	Shields
Nora	Shields
David	Shiers
Patti	Shih
Takeshi	Shimamoto
Carolyn	Shimmin
Grace	Shin
Grace Donghee	Shin
Ju Young	Shin
Christine	Shiner
Ken	Shinmura
Nathan	Shippee
Camila	Shirota
Noureen	Shivji
Olayinka	Shiyanbola
Stephanie	Shoop‐Worrall
Shahin	Shooshtari
Maayan	Shorer
Shefaly	Shorey
Stephanie D.	Short
Stephen	Shortell
Susan M.	Shortreed
Bonginkosi	Shozi
Svetlana	Shpiegel
M. Wade	Shrader
Archana	Shrestha
Bradley	Shrimpton
Kirstine	Shrubsole
Kirstine	Shrubsole
Jay H.	Shubrook
Janet	Shucksmith
Caroline	Shulman
James M.	Shultz
Wendy	Shulver
Lei	Si
Ching Sin	Siau
Shannon	Sibbald
Kathryn M.	Sibley
Margaret	Sibley
Miles	Sibley
Corianna Elizabeth	Sichel
Gemma	Sicouri
Jonathan	Sicsic
Najma	Siddiqi
Nicola	Sides
Nicola	Sides
Chris	Sidey‐Gibbons
*	Sidorova
Richard J.	Siegert
Rikke	Siersbaek
Joyce	Siette
Pallop	Siewchaisakul
Solrun	Sigurdardottir
Eloise	Silburn
Jonathan	Silcock
Laura	Sile
Carlos	Sillero
Diego S.	Silva
Luciana	Silva
Susana	Silva
Tricia Cleland	Silva
Tiago	Silva Jesus
Wagner	Silva‐Ribeiro
Maria	Silveira
Geoff	Silvera
Jonathan I.	Silverberg
Victoria	Silverwood
Nicholas J.	Silvestri
Sanju	Silwal
Jenny	Sim
Sara	Simblett
Magenta	Simmons
Michael	Simon
Tracey G.	Simon
Jane M.	Simoni
Efi	Simou
Alan	Simpson
Alan	Simpson
John	Simpson
Kati	Simpson
Robert	Simpson
Enver	Simsek
Joanie	Sims‐Gould
Jacqueline	Sin
Shane	Sinclair
Jonathan	Singer
Ilina	Singh
Kavita	Singh
Shweta	Singh
Simron	Singh
Swaran	Singh
Tarundeep	Singh
Abhishek	Singhai
Ann	Single
Katrin	Singler
Gaurav R.	Sinha
Mohd Zulkarnain	Sinor
Liron	Sinvani
Katya Y. J.	Sion
Sandra	Sipetic‐Grujicic
Anna	Sippel
A.	Siriwardena
Fuschia	Sirois
Giedrė	Širvinskienė
Andrea	Sisti
Regina W. S.	Sit
Manoj	Sivan
Jane	Sixsmith
Jane	Sixsmith
Samantha	Siyambalapitiya
Katarina	Sjögren Forss
Glykeria	Skamagki
Ane‐Marthe Solheim	Skar
Sarah	Skeen
Payam	Skeikhatari
Imogen	Skene
Suzanne	Skevington
Suzanne	Skevington
Adam	Skinner
Mark	Skinner
Caroline	Skirrow
Eva‐Maria	Skoda
Nicole	Skoetz
Samuel A.	Skootsky
Torild	Skrivarhaug
Magdalena	Skrybant
Tobias	Skuban‐Eiseler
David	Skvarc
Linda	Slack‐Smith
Mike	Slade
Helen	Slater
Peter	Slattery
Susan	Slatyer
Amanda	Slaunwhite
Åshild	Slettebø
Jude	Sligo
Melanie	Sloan
Jennifer	Sloane
Stephen	Smagula
Latoya A.	Small
Neil	Small
Nicola	Small
Natasha	Smallwood
Nicola	Smania
Luke R.	Smart
N. A.	Smart
E. M. A.	Smets
Tinne	Smets
Katarzyna	Smilowska
Amelia	Smit
Cees	Smit
Caitlin	Smith
Julia	Smith
Katharine A.	Smith
Rima K. Dhillon‐	Smith
Alexander	Smith
Brett	Smith
Catherine	Smith
Claire Friedemann	Smith
Corey	Smith
Cynthia	Smith
Debbie	Smith
Emily	Smith
Eve	Smith
Helen	Smith
James	Smith
Katie	Smith
Lorraine	Smith
Louise	Smith
Lucy	Smith
Maureen	Smith
Michael	Smith
Nick	Smith
Rebecca	Smith
Sian	Smith
Stephanie	Smith
Susan	Smith
Valerie	Smith
Stephanie	Smits
Elzbieta	Smolewska
Nina	Smythe
David	Snadden
Stephen	Snelders
Jeanne	Snelling
Helen	Snooks
Austyn	Snowden
Lonnie R.	Snowden
David	Snowdon
Jeremy	Snyder
Célia	Soares
Deanne	Soares
Diantha	Soemantri
Peter	Søgaard
Sze‐Ee	Soh
Dia	Soilemezi
Sophie	Soklaridis
Bhavana S.	Solanky
Jordan	Soliz
Suja	Somanadhan
Glenys	Somayajula
Kristian	Sommerfelt
Tae‐Jin	Song
Hyun	Song
Ranran	Song
Yuting	Song
Erica	Sood
Linn Marie	Sorbye
Marjorita	Sormunen
Felice	Sorrentino
Giovanni	Sotgiu
Christopher J.	Soto
Nelia	Soto‐Ruiz
Arthur D.	Soto‐Vásquez
Andrew	Soundy
Andre	Sourander
Nadia	Sourial
Paulo	Sousa
Kris	Southby
Emanuele	Souza Marques
Jonas	Spaak
Kerstin	Spanhel
Patricia	Spears
Brittany	Speller
Susan	Spence
Grace	Spencer
Kevin	Spencer
Angela	Sperotto
Johanna	Spiers
Michelle	Spirtos
Sandra L.	Spoelstra
Sharon	Spooner
Anneke	Spoorenberg
Esther	Sportel
Tanisha	Spratt
Julie	Spray
Georg Mathias	Sprinzl
Inge	Spronk
Tim	Sprosen
Martijn	Spruit
Eleni	Spyreli
Ray	Squires
Nickolas	Stabellini
Dawn	Stacey
Gemma	Stacey
Tania	Stafinski
Jona T.	Stahmeyer
Amanda E.	Staiano
Kristina	Staley
Britt‐Marie	Stålnacke
Sophie	Staniszewska
James	Stanley
Emma	Stanmore
Annette L.	Stanton
Rob	Stanton
Veronica	Stanton
Emily	Stapley
Sally	Stapley
Nicola Jayne	Starkey
Lauren T.	Starr
Sandra	Staudacher
William M.	Stauffer
Brian L.	Stauffer
Charitini	Stavropoulou
Charitini	Stavropoulou
Theresa	Stearn
Eric	Steegers
Nick	Steel
Gabriela	Stein
Mary Lyn	Stein
Katharine	Steinbeck
Katherine	Steinbeck
Simen A.	Steindal
Artur	Steiner
Lesley	Steinman
Suzanne	Steinman
Henry	Stelfox
Michael	Stellefson
Una	Stenberg
Barker	Stephanie
Kevin George	Stephenson
Vicky	Stergiopoulos
Brocha Z.	Stern
Antonio	Sterpetti
Esther	Steultjens
Melisa	Stevanovic
Sharon A. M.	Stevelink
Sebastian	Stevens
Fiona	Stevenson
Kay	Stevenson
Kerrie	Stevenson
David J.	Stewart
Derek	Stewart
Donna	Stewart
Ellen	Stewart
Iain	Stewart
Victoria	Stewart
Sarah	Stewart‐Brown
Mareike	Stiel
Chris	Stiff
Anne	Stiggelbout
Barbara	Stiglbauer
Colleen	Stiles‐Shields
Victoria	Stirrup
Jan Van den	Stock
Susan	Stockdale
Elizabeth M.	Stoeckl
Esther	Stoffers
Jonathan	Stokes
Tim	Stokes
Paul	Stolee
Samantha	Stonbraker
G.	Stone
Tony	Stone
Tracey	Stone
Charlotte R.	Stoner
Annerieke	Stoop
Nicole	Stormon
Nicole	Stormon
Al	Story
David	Story
William T.	Story
Ngaire Susan	Stott
Joshua	Stott
Angela	Stotts
Peter	Stoustrup
Daniel Llywelyn	Strachan
Gustav	Strandvik
Barbara	Strasser
Gregory P.	Strauss
Penelope	Strauss
Judith	Strawbridge
Jackie	Street
Maryann	Street
Richard	Street
Jean	Strelitz
Nicola	Strenzke
Paul A.	Stricker
Karen	Strickland
Emma	Strid
Ralf	Strobl
Fay J.	Strohschein
Katie A.	Strong
Karien	Stronks
Gillian	Strudwick
Jeroen	Struijs
Elizabeth A.	Stuart
Jennifer J.	Stuart
Dorothy	Stubbe
Jan	Stubberud
Brendon	Stubbs
Jamie L.	Studts
Matej	Stuhec
Simone	Stumpf
Nancy	Sturman
Andrew	Sturrock
Maria	Stuttaford
Jin	Su
Jing Jing	Su
Yakup	Subasi
Mohsan	Subhani
Indu	Subramanian
Dolly	Sud
May	Sudhinaraset
Rebecca	Sudore
Diarmuid D.	Sugrue
Jee Kyung	Suh
Mina	Suh
Sandra	Suijkerbuijk
J. Jill	Suitor
Sulistyawati	Sulistyawati
Donald R.	Sullivan
Amit	Summan
James F.	Sumowski
Fei	Sun
Hong	Sun
Tao	Sun
Tao	Sun
Wei	Sun
Vinay	Sundaram
Kay	Sundberg
Annelie J.	Sundler
Ann‐Sofie	Sundqvist
Valerie	Sung
David	Sunkersing
Alessandra	Surace
Anastacea	Suraev
Sophie	Suri
Joan Carles	Suris
Clare	Surr
Nirmal	Surya
Yayi	Suryo Prabandari
Brandstetter	Susanne
Katelyn	Sushko
Indri Hapsari	Susilowati
Ezra	Susser
Melanie	Sustersic
Panggung	Sutapa
Caroline	Sutcliffe
Adam	Sutherland
Brad A.	Sutherland
Rebecca	Sutherland
Liz	Sutton
Anne‐Marie	Suutari
Cindy	Suveg
Mats	Svantesson
Mia	Svantesson
Petra	Svedberg
Maria	Svedbo Engström
Katarina	Swahnberg
Laura	Swaithes
Veronica	Swallow
Lakshmi	Swaminathan
Dawn	Swancutt
Christian	Swanna
Angela	Sweeney
Mary Rose	Sweeney
Jennifer	Sweetman
W. E.	Swildens
Laura	Swink
Julie A.	Switzer
Sarah A.	Sydlowski
Kate	Sykes
Susie	Sykes
Alison	Symington
Anneliese	Synnot
Malin Olsen	Syversen
Palma	Szabo
Shelagh M.	Szabo
Felipe	Szabzon
Andrea	Szasz
Eszter	Szilassy
Gergő József	Szőllősi
Máire O'Donnell	T.
Søren	T. Skou
Katherine	T. Foster
Oren	T. Guttman
Nicole	T. M. Hill
Andrea	Tacchino
Getachew Redeae	Taffere
Louis	Taffs
Louis	Taffs
Louis	Taffs
Anna	Tagliabue
Bahareh	Tahani
Tamara L.	Taillieu
Ming	Tai‐Seale
Yoshiteru	Takekita
Mohammad	Talaei
Preetham	Talari
Amelia	Talbot
Catherine	Talbot
Sainz	Talia
Ambrose	Talisuna
Koji	Tamai
Alicia	Tamarit
Jeanette	Tamplin
Darrell H. S.	Tan
Gene S.	Tan
Kyle K. H.	Tan
Maw Pin	Tan
Tze‐Woei	Tan
Masaru	Tanaka
Mary Anne	Tanay
Kimberson	Tanco
Marija	Taneska
Chengxiang	Tang
Eugene Y. H.	Tang
Jean Y.	Tang
Qi	Tang
Shixing	Tang
Sydney Chi Wai	Tang
Yi‐lang	Tang
Zhongjun	Tang
Viroj	Tangcharoensathien
Randi J.	Tangvik
Ahmet	Tanhan
Ahmet	Tanhan
Bubela	Tania
Thitipong	Tankumpuan
Bernadette	Tanner
Denise	Tanner
Mylène	Tantchou Dipankui
Ekamol	Tantisattamo
Fangbiao	Tao
Hazel	Tapp
Howard	Taras
Lionel	Tarassenko
Edith A. M.	Tarimo
Raúl	Tárraga‐Mínguez
Carolyn	Tarrant
Burak	Taşcı
Saima	Tasneem
Sevinc	Tastan
Camilla	Tatari
Judith	Tate
Oktay	Tatlıcıoğlu
Elizabeth Barbara	Tatsi
Athina	Tatsioni
Michael J.	Tatterton
Orit	Taubman‐Ben‐Ari
Kouhyar	Tavakolian
Lara	Tavoschi
Ibrahim	Tawhari
Djin	Tay
Anna	Taylor
Denise	Taylor
Francesca	Taylor
Graham	Taylor
J.	Taylor
Jo	Taylor
Joseph J.	Taylor
Judy	Taylor
Kate	Taylor
Nicholas	Taylor
Rachel M.	Taylor
Rod S.	Taylor
Scott J.	Tebbutt
Helena	Teede
Lorynn	Teela
Henok Getachew	Tegegn
Martina	Teichert
Stefan	Teipel
Natacha	Teissier
Ana C.	Teixeira‐Santos
Jeff R.	Temple
Kevin	ten Haaf
Leontine W.	Ten Hoopen
Chantal	ten Kate
Tim	Tenbensel
Olli	Tenovuo
P.	Teodoroski
Piotr	Teodorowski
Anja	Terkamo‐Moisio
Gijs	Terlouw
Julia	Terry
Handan	Terzi
Stefano	Terzoni
Ephrem	Tesfay
Martin	Tesli
June M.	Tester
Nicolette	Teufel‐Shone
Heather D.	Tevendale
Garry A.	Tew
Megan	Teychenne
Megan	Teychenne
Caterina	Tezze
Neeta	Thakur
Kalpana	Thapa Bajgain
C.	Thayer
Mariza Miranda	Theme Filha
Tim N.	Theologis
Linda	Theron
Jolin	Thijs
Vincent	Thijs
Janani	Thillainadesan
Kaeshaelya	Thiruchelvum
David	Thomas
Emma	Thomas
Janet	Thomas
Judith	Thomas
Kate	Thomas
Kathleen	Thomas
Kristina	Thomas
Kristina	Thomas
Mathew	Thomas
Rae	Thomas
Stephen	Thomas
Victoria	Thomas
Andrew	Thompson
Carl	Thompson
Jemima	Thompson
Jill	Thompson
Sarah	Thomsen
David R.	Thomson
Gill	Thomson
Peter James	Thomson
Johan	Thor
Sally	Thorne
Clifton P.	Thornton
Hazel	Thornton
Rune	Thorsen
Eric	Thouvenot
James F.	Thrasher
Jochen Rene	Thyrian
Cindy Yue	Tian
Bea	Tiemens
Stephanie	Tierney
Caroline	Tietbohl
Kirsi	Tiitinen Mekhail
Binyam	Tilahun
Bram	Tilburgs
Benedikt	Till
Aura	Timen
Anne	Timm
Danielle	Timmermans
Danielle	Timmermans
Yuriy	Timofeyev
Jonathan	Timpka
Tin	Tin Su
Sunny priyatham	Tirupathi
Carol	Tishelman
Malin	Tistad
Tyler J.	Titcomb
Olaoye	Titilayo
Ingrid	Tjoflat
Georgia	Tobiano
Christine	Tocchi
Adam	Todd
Elka	Todorova
Nicole	Toepfner
Leanne	Togher
Stefania	Tognin
Fumiharu	Togo
Sengwee	Toh
Anniina	Tohmola
Eylem	Toker
Miroslava	Tokovska
Yasuharu	Tokuda
Wietse A.	Tol
Angela	Tolotti
Madalina	Toma
José	Tomás Mateos
Naoki	Tomita
Justine	Tomlinson
A.	Tong
Allison	Tong
Huei Jinn	Tong
Martina	Toni
Sofia	Toniolo
Seda	Topçu
Alexis	Topjian
Stephanie	Topp
Annie	Topping
Michelle	Torok
Els	Torreele
Irene	Torres
Thiago S.	Torres
Alezandra	Torres‐Cataño
Francesca J.	Torriani
Umar	Toseeb
Thomas	Tourdias
Ann‐Marie	Towers
Ellen	Townsend
Liam	Townsend
Tiffany G.	Townsend
Francine	Toye
Safiye	Tozdan
Michael	Toze
Manuel	Trachsel
C.	Tracy
Derek	Tracy
Derek K.	Tracy
Marguerite	Tracy
Michael W.	Traeger
Denise D.	Tran
Phuong Bich	Tran
Mette	Tranberg
Joanne	Travaglia
Victoria	Traynor
C.	Treanor
John	Tredinnick‐Rowe
Horst	Treiblmaier
Katherine	Treiman
Marie‐Claude	Tremblay
Steve	Trenoweth
Lyndal	Trevena
Evangelos	Triantaphyllou
Andrea	Triccoa
Heather	Trickey
Ines A.	Trindade
Jonathan	Tritter
Roberto	Tron
Wendy	Troxel
Isabela	Troya
Emily A.	Troyer
Mathieu	Trudel
Inga	Truskauskaite‐Kuneviciene
Hsiu Ting	Tsai
Meng‐Han	Tsai
Eileen	Tsai Chambers
Lena	Tschiderer
Komla	Tsey
Vicki	Tsianakas
Argerie	Tsimicalis
Elena	Tsoy
Sian Hsiang‐Te	Tsuei
Alina	Tsyrulnik
Astrid van	Tubergen
Gerhard	Tucekv
Jose	Tuells
Irene	Tuffrey‐Wijne
Remco	Tuijt
David S.	Tulsky
James	Tulsky
Elena	Tulupova
Sri Lekha	Tummalapalli
Suat	Tuncay
Isaac	Túnez
Helena	Tuomainen
Philippe	Tuppin
Gülcan Bahçecioğlu	Turan
Erin	Turbitt
Eleanor	Turi
Fidan	Turk
Lyn	Turkstra
Aaron	Turner
Dan	Turner
Daniel	Turner
Katrina	Turner
Martin	Turner
Maria Grazia	Turri
Stephanie	Turrise
Perri R.	Tutelman
Emily L.	Tuthill
Leah	Tuzzio
Anne Therese	Tveter
Jacqueline	Twamley
Maureen	Twiddy
Diane E.	Twigg
Shilpa	Tyagi
Tanja	Tydén
Natasha	Tyler
Anna	Tynan
Marie	Tyrrell
D.	Ubbink
D. T.	Ubbink
Taslim	Uddin
Michael	Udedi
Anna	Ugalde
Marte‐Mari	Uhlen‐Strand
Till	Uhlig
Laura	Uhlmann
Shahid	Ullah
Connie M.	Ulrich
Nosheen	Umar
Deborah	Ummel
Janelle	Unger
Karla	Unger‐Saldaña
Regine	Unkels
Constance R.	Uphold
Ross	Upshur
Jane	Upton
Tomohiko	Urano
Christine	Urquhart
Wayne	Usher
Juliet	Usher‐Smith
Timothy	Usherwood
Silvia	Ussai
Jane M.	Ussher
Michael	Ussher
Rebecca	Utz
Edward	V. Nunes
Lonneke	V. Van De Poll‐Franse
Stefanie J.	Vaccher
Bert	Vaes
Monia	Vagni
Laura	Vagnoli
Birgit	Vahlberg
Ishfaq	Vaja
Maryam	Valapour
Jose María	Valderas
Emanuelle Pessa	Valente
Michael A.	Valente
Luca	Valenti
Katherine	Valentine
Patricia	Valery
Maritta	Välimäki
Lisa M.	Vallely
Thomas S.	Valley
Michael	Vallis
Theo	van Achterberg
Heleen	van Agt
Chantal	Van Audenhove
Chantal	Van Audenhove
Marloes	van Bokhoven
Jackie	van Dael
Jackie	van Dael
Hester	van de Bovenkamp
Elise	van de Putte
J. Merlijn	van den Berg
Carissa	Van den Berk‐Clark
Maria	van den Muijsenbergh
Anna	van der Gaag
Peter	Van der Graaf
Rieke	van der Graaf
Marjolein	Van der Marck
Paige	van der Pligt
Lotte	van der Stap
Jenny	van der Steen
Sabina	van der Veen
Veronika	van der Wardt
Trudy	van der Weijden
Trudy	van der Weijden
Jerôme	van Dongen
Sandra	van Dulmen
Kathryn	Van Eck
Dwayne	Van Eerd
Nicole	van Erp
Job	Van Exel
Lisette	Van Gemert‐Pijnen
Kimberley	Van Haitsma
Caroline M.	van Heugten
Robin	van Kessel
Karen M.	van Leeuwen
Emile	van Lin
Tim	Van Mieghem
Hedy Aline	Van Oers
Kimberly A.	Van Orden
Sandra	van Os
Hendrik	Van Poppel
Romy	Van Rickstal
Lotte	van Rijn
Manya	van Ryneveld
Douwe	Van Sinderen
Inge	van Strien‐Knippenberg
Julia	van Tol‐Geerdink
Georgia	Van Toorn
David E.	Vance
Michelle M.	Vance
Virginia	Vandall‐Walker
Pieter	Vandekerckhove
Robert	Vander Stichele
Shelley	Vanderhout
Jennifer	Vanderlaan
Ashley	Vandermorris
Maaike	Vandermosten
Sien	Vandesande
Tushna	Vandrevala
Tushna	Vandrevala
Amanda	Vandyk
Sabine	Vaneerdenbrugh
Michael	VanNostrand
Meredith	Vanstone
Meredith	Vanstone
Varshini	Varadaraj
Colleen	Varcoe
Ingrid	Vargas
Lina María	Vargas‐Escobar
Ana Beatriz	Vargas‐Santos
Julian	Varghese
Boerwinkle	Varina
Aleksi	Varinen
Maria	Varkanitsa
Semra Navruz	Varlı
Ravi Prasad	Varma
Anubodh S.	Varshney
Jorge	Vásconez
Ivaylo	Vassilev
Jason	Vassy
Lidewij	Vat
Elena	Vaughan
Priya	Vaughan
Peter G.	Vaughan‐Shaw
Lisa M.	Vaughn
Verónica	Vázquez‐Velázquez
Andres	Vecino‐Ortiz
Christopher A.	Veeh
Haske van	Veenendaal
Marja	Veenstra
Prabhakar	Veginadu
Roque Anthony	Velasco
Esther	Veldhoen
Cindy B.	Veldhuis
J.	Veldwijk
Diana	Velikonja
Shirin	Vellani
Alisa	Velonis
Kartik K.	Venkatesh
Jane	Vennik
Peter	Ventevogel
Maria Amelia	Veras
Michèle	Verdonck
Manisha	Verma
Stefanie	Vermaak
Karin	Vermeulen
Neeltje	Vermunt
Sebastian	Vernal
M.	Vernooij‐Dassen
Nicola	Veronese
Olaf	Verschuren
Judith	Versloot
Sigrid C. J. M.	Vervoort
Amanda	Vest
Mario Vianna	Vettore
Jennifer	Viberg Johansson
Isabel	Vicario‐Molina
Christina	Victor
Everard G. B.	Vijverberg
Petter	Viksveen
Mireya	Vilar‐Compte
Melissa	Vilaro
Carlos	Vílchez‐Román
Victor Eduardo	Villalobos‐Daniel
David	Villarreal‐Zegarra
Karen	Vinall‐Collier
Rasita	Vinay
Peter	Vincent‐Jones
Cecilia	Vindrola‐Padros
Russell	Viner
Clara	Virbel‐Fleischman
Claudia	Virdun
Daniel	Virella
Wiseman	Virginia
Anita	Visser
John	Vissing
Giulia	Vivaldi
Cristofol	Vives‐Bauza
Carmen	Vives‐Cases
Katie	Vlaardingerbroek
Druel	Vladimir
Thea	Vliet Vlieland
Nicoline B. M.	Voet
Jonathan	Vogelgsang
Susanne	Vogl
Henrik	Vogt
Katharina Sophie	Vogt
Shetal	Vohra‐Gupta
Angelo	Volandes
Špela	Volčanšek
Ana	Volkmer
Christian	von Wagner
Sebastian	von Peter
Laura A.	Vonnahme
Benjamin Van	Voorhees
Paula	Voorheis
Sabine	Voorst
Martina	Vortel
Rimke	Vos
R. Wesley	Vosburg
Ana‐Maria	Vranceanu
Charikleia S.	Vrettou
Hubertus	Vrijhoef
Cecile	Vuillermoz
Charles	W. Helsper
Vivian	W. Q. Lou
Katherine	W. Scangos
Jan	W. van der Scheer
Justine	W. Welsh
Alex	W. K. Wong
Johannes	Wach
Luca J.	Wachtendorf
Julia	Wade
Margda	Waern
N.	Wah Cheung
Shabab	Wahid
Eka Julianta	Wahjoepramono
Astrid	Wahl
Sina	Waibel
Gavisha	Waidyaratne
Tom	Wainwright
Felicity	Waite
Polly	Waite
Benna	Waites
Fathima	Wakeel
Claire E.	Wakefield
Rie	Wakimizu
Lauren S.	Wakschlag
Tobias	Walbert
Sarah Cusworth	Walker
Ashby F.	Walker
Christine	Walker
Dawn‐Marie	Walker
Elizabeth Reisinger	Walker
Meghan J.	Walker
Melanie	Walker
Rob	Walker
Tammi	Walker
Verónica García	Walker
Dariusz	Walkowiak
Louise	Wallace
Sarah	Wallace
Margaret	Wallen
Amy	Waller
Daniel	Waller
Nina	Wallerstein
Andreas	Wallin
Madeleine	Walpert
Ramesh	Walpola
Jane C.	Walsh
Catherine	Walshe
Margaret	Walshe
Fiona	Walter
Kate	Walters
Helen	Walthall
Courtney C.	Walton
Angela	Wan
Ming	Wan
Thomas	Wan
Wen	Wan
Anne Pamela Frances	Wand
Aiping	Wang
Audrey	Wang
Beiyu	Wang
Cixin	Wang
Dahua	Wang
Debin	Wang
Honghong	Wang
Honghong	Wang
Jian	Wang
Jian‐Jun	Wang
Jingze	Wang
Kailu	Wang
Kefang	Wang
Kyu‐Chang	Wang
Lei	Wang
Li	Wang
Liang‐Jen	Wang
Lihua	Wang
Qian	Wang
Quan	Wang
Rui‐Wen	Wang
Shousen	Wang
Siquan	Wang
Wenhua	Wang
Xiaomin	Wang
Yao	Wang
Josefin	Wångdahl
Julian	Wangler
Jukkrit	Wangruth
Susitha	Wanigaratne
Ahmed	Waqas
Louise	Warburton
David D.	Ward
Helen	Ward
Jeremy K.	Ward
Marie	Ward
Michael J.	Ward
Michael J.	Ward
Hannah R.	Wardill
Liane	Wardlow
Justin	Waring
Stuart	Wark
J. Catja	Warmelink
Grace	Warner
Terri	Warner
Cynthia	Warren
Fiona C.	Warren
Leigh	Warren
Narelle	Warren
Petra	Warschburger
Damian	Warzecha
Karla T.	Washington
Marina B.	Wasilewski
Rebecca	Wassall
Danuta	wasserman
Brittany L.	Waterman
Jennifer	Watermeyer
Erika A.	Waters
Niamh	Waters
C. Nadine	Wathen
Bernadette	Watson
Daniella	Watson
Eila K.	Watson
James	Watson
James	Watson
Joanne	Watson
Kaitlyn E.	Watson
Linda	Watson
Lorraine	Watson
Paula	Watson
Verity	Watson
Ian S.	Watt
Jennifer A.	Watt
Kerrianne	Watt
Apichai	Wattanapisit
Inger	Wätterbjörk
Meaghann S.	Weaver
Edward J. D.	Webb
Marianne	Webb
Rebecca	Webb
Martin	Webber
Sandra C.	Webber
Silvana	Weber
Angela C.	Webster
Rob	Webster
Marina	Weckend
Annalise	Weckesser
Liang En	Wee
Salima Van	Weely
Himali	Weerahandi
Swarna	Weerasinghe
Maria	Węgrzynowska
George L.	Wehby
Immo	Weichert
Wendy	Weidner
Catherine	Weil‐Olivier
Barbette	Weimer‐Elder
Kate	Weiner
Saul	Weiner
Marc J.	Weintraub
Heather	Weir
Kristie	Weir
Heide	Weishaar
Marianne	Weiss
Ruth Striegel	Weissman
Anthonie J. van der	Wekken
Carly	Welch
Lindsay	Welch
Michael B.	Wells
Yvonne	Wells
Marie‐Laure	Welter
Madison	Wempe
Chuan	Wen
Yvonne	Wengstrom
Michel	Wensing
Michel	Wensing
Dianne	Wepa
Thomas A.	Werfel
Ulla	Werlauff
Nicole E.	Werner
Perla	Werner
Alexandra	Werntz
Amy E.	West
Emily	West
Gerben	Westerhof
Max J.	Western
Nicholas J.	Westers
Heleen	Westland
Simone	Weyers
Rebecca	Whear
Alison	Wheatley
Amanda	Wheeler
Jeremy	Whelan
Kevin	Whelan
Pauline	WHelan
Paul K.	Whelton
Katriina	Whitaker
Carole L.	White
Carolynne	White
Kate	White
Peter	White
Simon	White
Phyllis	Whitehead
Rick	Whitehead
Andrew	Whitehouse
William	Whitehouse
Tim	Whitfield
Carly	Whitmore
Simon	Whitney
Maxine A.	Whittaker
Sarah W.	Whitton
Christine M.	Wickens
Nilmini	Wickramasinghe
Paul	Wicks
Jessica	Widdifield
Ellen	Wiebe
Sietse	Wieringa
Nora	Wiium
Anna	Wikman
Jane	Wilbur
Kyle	Wilby
Cervantee	Wild
Josephine M.	Wildman
Louise	Wiles
Kay	Wilhelm
Radka	Wilhelmová
Sarah	Wilkes‐Gillan
Jo	Wilkie
David	Wilkinson
Shelley A.	Wilkinson
Thomas J.	Wilkinson
Thomas J.	Wilkinson
Agnes	Willemen
Suzanne M.	Willey
Cathleen	Willging
Scott	William
Venice Ng	Williams
Andrew	Williams
Anne Sved	Williams
Arthur Robin	Williams
Cynthia	Williams
Deni	Williams
Faustine	Williams
J. M.	Williams
Jane	Williams
Lauren	Williams
Nathaniel J.	Williams
Nefyn	Williams
Oli	Williams
Pauline	Williams
Randi M.	Williams
Ryan	Williams
Simon	Williams
Summer	Williams
Venice Ng	Williams
Veronika	Williams
Victoria	Williamson
Paula	Williamson
Timothy J.	Williamson
Karen	Willis
Paul	Willis
Lindy	Willmott
Celia	Wills
Wendy	Wills
Janina	Wilmskoetter
Maciej	Wilski
Brenda	Wilson
Ceri	Wilson
David C.	Wilson
Eleanor	Wilson
Ewan	Wilson
Ira	Wilson
Jefferson	Wilson
Natalia	Wilson
Rhonda	Wilson
Sue	Wilson
Christine Brown	Wilson
Anders	Wimo
Sula	Windgassen
Joseph W.	Windsor
Rachel	Winer
Gemma	Winsor
Paul	Winston
Tania M.	Winzenberg
Ruth	Wise
Catherine	Wiseman‐Hakes
Kirsten	Wisner
Maartje de	Wit
Nishadi	Withanage
Kaila G. K.	Witkowski
Stefanie	Witt
Hans‐Ulrich	Wittchen
Holly	Witteman
Walter	Wittich
Anja	Wittkowski
Daniela	Wittmann
Andre P.	Wolff
Jennifer	Wolff
Jennifer	Wolff
Courtney Benjamin	Wolk
Dieter	Wolke
Alex	Wolkow
Steven	Woloshin
René	Wolters
Emma	Wolverson
Dana	Wong
Eliza Lai‐yi	Wong
Geoff	Wong
Jennifer	Wong
Joshua	Wong
Ling Ling Beatrix	Wong
Sabrina	Wong
Ambroise	Wonkam
Karen	Woo
Fiona	Wood
James	Woodall
Charlotte	Woodcock
Joanne	Woodford
Roberta	Woodgate
Erica	Woodin
David	Woodley
Stephanie J.	Woodley
Chris	Woodrow
Matthew	Woodruff
Bob	Woods
Catherine	Woods
Lee	Woods
Abi	Woodward
Sue	Woodward
Anthony	Woolf
Steven H.	Woolf
Kerry	Woolfall
Sue	Woolfenden
Tom	Workman
Eveline	Wouters
Andrea	Wray
Selina	Wray
Laura	Wray‐Lake
Bruce	Wright
Judith	Wright
Kath	Wright
Michael	Wright
Feifeng	Wu
Leihong	Wu
Peng	Wu
Pensee	Wu
Qunhong	Wu
Xi Vivien	Wu
Yijin	Wu
Katrina	Wyatt
Marianne	Wyder
Eleanor	Wyke
Karen	Wynter
Ioanna	Xenophontes
Di	Xiao
Hong	Xiao
Lily D.	Xiao
Bo	Xie
Chenjie	Xu
Richard	Xu
Tina	Xu
Baowen	Xue
Rui	Xue
Carol	Y. Ochoa‐Dominguez
K. Robin	Yabroff
Vijayshree	Yadav
David B.	Yaden
Asli Cennet	Yalim
Sosei	Yamaguchi
Tetsuya	Yamamoto
Noriko	Yamamoto‐Mitani
Svetlana	Yampolskaya
Tatiane	Yanes
Dong Won	Yang
Fu‐Chi	Yang
Grace Meijuan	Yang
Qian	Yang
Xiaoyu	Yang
Zhihao	Yang
Elizabeth	Yano
Xueli	Yao
Yao	Yao
Zeynep Öztürk	Yaprak
Erkan	Yarımkaya
Ashley C.	Yaugher
Feng	Ye
Samantha	Yeager
Philippa	Yeeles
Maria	Yefimova
Cansu Yilmaz	Yegit
Shu‐Chuan Jennifer	Yeh
Ying‐Chih	Yeh
Renata	Yen
E.	Yeoh
Müjdat	Yeşildal
Huso	Yi
Jia	Yi
Gülhan	Yiğitalp
Dr. Tegbar	Yigzaw
Volkan	Yilmaz
Ece	Yilmaz Kara
Shonna	Yin
Terry Cheuk‐Fung	Yip
Li	Yi‐Ting
Wai Han	Yiu
Galit	Yogev‐Seligmann
Dae‐Hyun	Yoo
Jee‐Hye	Yoo
Byungun	Yoon
Sungwon	Yoon
Ahtisham	Younas
Adrienne	Young
Adrienne M.	Young
Bridget	Young
James J.	Young
Min	Young Kim
James J.	Young
Zohaib	Yousaf
Mansour	Youseffi
Reza	Yousefi Nooraie
Valmae	Ypinazar
Alexander	Yu
Hongjie	Yu
Jane Jie	Yu
Ping	Yu
Siyang	Yuan
He	Yuanyuan
Kris	Yuet‐Wan Lok
Mustafa	Yüksel
Chaiyaporn	Yuksen
Young Ho	Yun
Azlina	Yusuf
Sara	Zabeen
Faith	Zabek
Kori S.	Zachrison
Haroon	Zafar
Francesco	Zaghini
Whitney E.	Zahnd
Victor	Zaia
Alexandra	Zaleta
Ellen	Zambo Anderson
Laura D.	Zambrano
Giovanna	Zambruno
Mauro	Zampolini
Eduardo	Zancul
Faika	Zanjani
Tobia	Zanotto
Paola	Zaratin
Cristina	Zarbo
Mikołaj	Zarzycki
Douglas	Zatzick
Isabel	Zbukvic
Anna	Zdolska‐Wawrzkiewicz
Emmanuelle	Zech
Amy J.	Zeidan
Elisabeth L.	Zeilinger
John M.	Zelenski
Steven	Zeliadt
Aurel	Zelko
Erika	Zelko
Menno van	Zelm
Glenn	Zemel
Yongyi	Zeng
Natasa	Zenic
Ian	Zenlea
Rachel	Zeuner
Bo	Zhang
Caiyi	Zhang
Haohan	Zhang
Nanhua	Zhang
Shuai	Zhang
Shujing	Zhang
Yanchen	Zhang
Yingzi	Zhang
Ivy Yan	Zhao
Mirabella	Zhao
Shi	Zhao
Yali	Zhao
Campion	Zharima
Bang	Zheng
David X.	Zheng
Han	Zheng
Shuting	Zheng
Sujun	Zheng
Xiaomin	Zhong
Yaping	Zhong
Mengping	Zhou
Xiaoyu	Zhou
Dawei	Zhu
Donna S.	Zhukovsky
Nida	Ziauddeen
Stefanie	Ziehfreund
Tjalf	Ziemssen
Alyssa	Ziman
Julia‐Marie	Zimmer
Melanie	Zimmer‐Gembeck
Emily B.	Zimmerman
Hanna Gwendolyn	Zimmermann
Jens O.	Zinn
Yaara	Zisman‐Ilani
Julie	Zissimopoulos
Nick	Zonneveld
Szilvia	Zörgő
Alina	Zorina Stroe
Jan	Zottmann
Huachun	Zou
Eliana Miura	Zucchi
Jessie Nallely	Zurita‐Cruz
Sandra	Zwakalen
Jennifer D.	Zwicker
Anita van	Zwieten

